# Photo-Assisted Flexible Energy Storage Devices: Progress, Challenges, and Future Prospects

**DOI:** 10.1007/s40820-025-01964-1

**Published:** 2026-01-12

**Authors:** Xupu Jiang, Ting Ding, Rui Wang, Wujun Ma, Chuntao Lan, Min Li, Meifang Zhu

**Affiliations:** 1 College of Textile and Garment, Nantong University, Nantong, 226019 People’s Republic of China; 2 State Key Laboratory of Advanced Fiber Materials, Donghua University, Shanghai, 201620 People’s Republic of China

**Keywords:** Photo-assisted energy storage, Flexible devices, Photoelectrochemical mechanisms, Electrodes

## Abstract

Photo-assisted flexible energy storage devices, combining photoelectric conversion and electrochemical energy storage, emerge as an innovative solution for sustainable energy systems. This review comprehensively summarizes recent advances in photo-assisted flexible energy storage technology, covering material design, working mechanisms, and practical applications. We systematically examine diverse electrode materials, such as metal oxides, metal sulfides, organic photosensitive materials, and composites, emphasizing their roles in boosting device performance. Special focus is placed on emerging technologies—including heterostructure engineering, surface modification, and intelligent control systems—that have notably enhanced energy conversion efficiency and storage capacity. The review also discusses current challenges, such as material stability, conversion efficiency, and standardization, and proposes strategic directions for future development. Recent breakthroughs in photo-assisted supercapacitors, lithium-based batteries, zinc-based batteries, and other innovative storage systems are critically assessed, offering key insights into their practical application potential in wearable electronics, self-powered sensors, and beyond. This comprehensive analysis establishes a framework for understanding the current status of photo-assisted flexible energy storage technology and guides future research toward high-performance, sustainable energy storage solutions.

## Highlights

- This review provides a comprehensive integration of photoconversion and electrochemical storage mechanisms for flexible wearable applications.
- It systematically classifies and compares various flexible light-assisted energy storage systems—from supercapacitors to diverse metal batteries—within a unified framework.
- The review highlights advanced material design strategies and performance enhancement techniques specifically tailored for light-responsive energy storage, including heterojunctions, doping, and nanostructures.

## Graphical abstract

**Figure:**
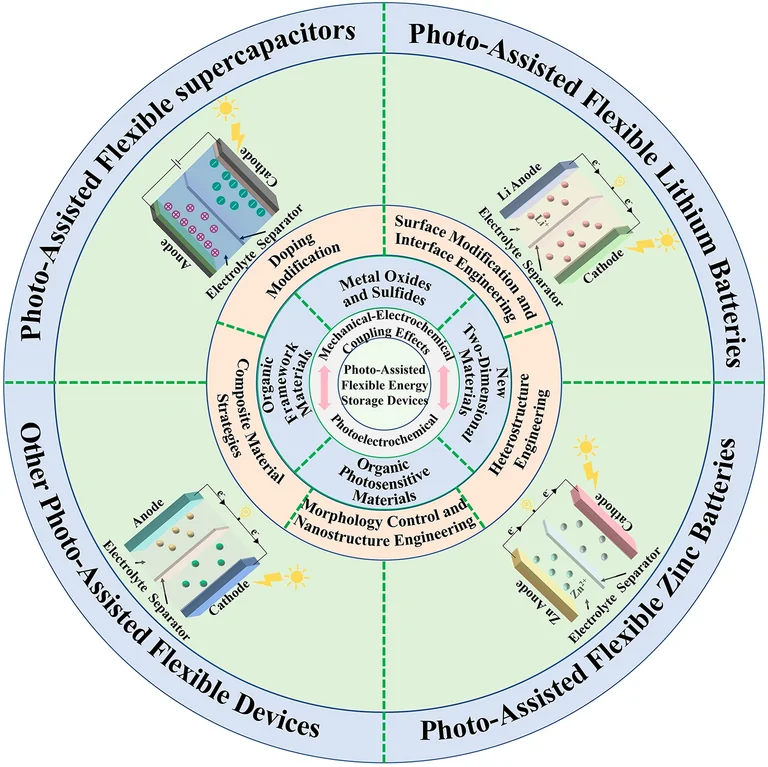

## Introduction

Biological systems, honed over millennia, offer profound inspiration for light energy conversion, from the intricate photosynthetic machinery in plants to the captivating glow of bioluminescence [1]. This natural mastery motivates the development of innovative energy storage strategies that emulate these efficient light utilization patterns. The rapid proliferation of wearable and portable electronic devices has driven an urgent demand for advanced energy storage systems. These systems must not only exhibit high energy/power densities but also seamlessly integrate mechanical flexibility and photoresponsive functionality [2, 3]. Unlike conventional approaches that often segregate photovoltaic energy capture and electrochemical energy storage into distinct, rigid components, photo-assisted flexible energy storage devices represent a transformative paradigm. These integrated systems are engineered to simultaneously harvest ambient light energy and store it electrochemically. Their architectures can undergo significant mechanical deformation, including bending, stretching, and folding [4–7].

Photo-assisted flexible energy storage devices are a distinct class of systems. Their defining feature is the synergistic integration of mechanical adaptability and light responsiveness. Mechanical adaptability is critical for operation in dynamic, non-planar environments, while light responsiveness enables incident photon irradiation to directly enhance energy storage performance via photo-induced charge generation, carrier modulation, or thermal effects at the electrode–electrolyte interface. This process typically entails the strategic integration of photoactive electrode materials (e.g., semiconductors, dyes, quantum dots) with compliant, flexible substrates (e.g., polymers, textiles, thin metals). This integration enables seamless coupling of energy-harvesting and storage functionalities in mechanically adaptive architectures [8–10]. The concept of light-assisted energy storage originated from early innovations in 2004, which led to the development of the first dual-electrode light-driven capacitor. This technology combines dye-sensitized nanocrystal films with activated carbon layers, achieving significant capacitance values and cycle stability. Subsequently, various light-assisted energy storage devices were developed [11]. Ding et al. integrated a photoelectrode and a working electrode to form a nano-porous Cu@Cu_2_O (NPC@Cu_2_O) hybrid array electrode, thereby preparing a light-assisted rechargeable supercapacitor. Under illumination, the supercapacitor achieved a specific capacitance of 782 F g^−1^, representing a 37.9% increase compared to the dark state [12]. Wu et al. introduced the first three-electrode photo-assisted Li–O_2_ battery system, effectively addressing the critical overpotential challenges in non-aqueous lithium-oxygen batteries through integrated photoelectric conversion and energy storage components [13]. Following these foundational works, the field expanded to encompass diverse battery chemistries including Li–I solar flow batteries [14], Li–S systems [15], rechargeable Zn-air batteries [16, 17], and metal-CO_2_ configurations [18–20], each leveraging photoassistance to address specific electrochemical limitations in rigid device formats.

However, transitioning from established rigid configurations to flexible photo-assisted systems introduces unprecedented engineering and fundamental scientific challenges. These challenges, which define the emerging field and distinguish it from rigid predecessors, extend beyond the sole focus on optimizing static-state photoelectrochemistry [21, 22]. Crucially, these challenges involve: First, maintaining robust photoelectrochemical performance under dynamic mechanical deformation is critical. Under such conditions, strain, bending, and torsion can profoundly alter the electronic band structure of photoactive materials, induce microcracks that disrupt charge transport pathways, modify optical properties (thereby affecting light absorption), and alter electrode–electrolyte interface kinetics [23]. Second, ensuring durable interface stability between disparate materials under cyclic mechanical stress is essential [24], as the interfaces between rigid or brittle photoactive layers, flexible current collectors, and polymeric substrates are particularly vulnerable to delamination, fatigue, and wear when subjected to repeated bending or stretching, leading to increased internal resistance and device failure [25]. Third, achieving system-level integration to meet the multifaceted demands of wearable applications is paramount. This integration must encompass not only flexibility and stretchability but also conformability to curvilinear surfaces, lightweight design, long-term operational stability under real-world mechanical stimuli, and often, biocompatibility [26]. Consequently, despite their promise, current flexible devices still face critical limitations arising from these challenges. These include significantly reduced photon-to-electron conversion efficiencies under mechanical stress, inadequate long-term durability during extensive deformation cycles, and often insufficient energy and power densities to meet the demands of practical wearable applications [27]. Addressing these fundamental challenges requires systematic investigation of advanced materials, innovative device architectures, and novel integration strategies, which form the focus of this comprehensive review.

Unlike previous reviews that primarily focus on rigid photo-assisted energy storage systems, this review specifically addresses the emerging field of flexible photo-assisted energy storage devices, which necessitates fundamentally different design principles and performance optimization strategies. The mechanical deformability constraints demand novel approaches to material selection, device architecture, and interface engineering that extend far beyond conventional rigid system considerations. The design criteria for light-assisted flexible energy storage systems and light-assisted rigid energy storage systems are compared in Table 1. The advantages of light-assisted flexible energy storage devices over heat-assisted and electricity-assisted devices are shown in Table 2. This review provides a comprehensive analysis by systematically examining advanced material design strategies, fundamental working mechanisms under mechanical deformation, and innovative device architectures with emphasis on wearable applications. Particular focus is placed on critical challenges and breakthrough solutions for maintaining robust photoelectrochemical performance under mechanical stress, including advanced interface engineering, morphology control, and composite design strategies. Through this systematic investigation of progress, current limitations, and emerging opportunities, this review aims to guide the rational development of next-generation flexible photo-assisted energy storage technologies that can meet the demanding requirements of wearable and conformable energy systems.

**Table 1:** Comparison of design criteria for light-assisted flexible energy storage systems and light-assisted rigid energy storage systems

Design guidelines	Light-assisted rigid energy storage systems		Light-assisted flexible energy storage systems
Core design objectives	Pursuing high photoelectric conversion efficiency, high energy density, and long-term static stability	Balancing optoelectronic performance and mechanical flexibility to ensure consistent performance under deformation and long-term reliability	
Base material	Hard substrates (such as glass, ceramics, rigid metal substrates) provide structural support but cannot be deformed	Flexible substrates (such as carbon cloth, polyimide film, polyurethane foam) that combine conductivity and resistance to bending	
Light absorbing layer material	Traditional semiconductors (such as TiO_2_, CdTe) focus on light absorption coefficient and carrier mobility	Flexible light-responsive materials (such as conductive polymers pTTh, two-dimensional materials MoS_2_, MXene) that combine flexibility and photoactivity	
Electrode structure	Rigid layered stacking (such as flat electrode + light-absorbing layer + electrolyte), with a fixed structure and rigid interface connection	3D porous/wrinkled structures (such as nanotube arrays and flower-like nanostructures) reserve deformation space and reduce stress concentration	
Electrolyte type	Liquid electrolytes have high ion conduction efficiency but require strict sealing	Gel/solid electrolyte, which combines ionic conductivity and elasticity, prevents leakage, and adapts to deformation	
Optoelectronic performance indicators	Focus: Photovoltaic conversion efficiency, energy density, static cycle stability	Focus: Photovoltaic efficiency retention under deformation, light-assisted charge–discharge rate, performance degradation rate after bending cycles	
Mechanical performance requirements	No flexible requirements, resistant to static stress	Must withstand multidimensional deformation: bending angle, bending cycle count, compression strain	
Package design	Rigid sealed enclosure (such as metal/glass encapsulation), with emphasis on corrosion resistance and long-term sealing	Flexible packaging materials (such as medical tape and waterproof breathable film) must balance sealing properties with deformation adaptability	
Typical application scenarios	Stationary energy storage (such as photovoltaic grid-connected storage), static solar charging equipment	Wearable electronics (such as smart bracelets and flexible watches), portable devices (such as foldable solar power banks), and flexible sensor power supplies	
Key challenges	Large volume, low integration, difficult to adapt to mobile scenarios; brittle materials can easily lead to structural failure under thermal cycling	Material compatibility (such as the bonding strength between the flexible substrate and the light-absorbing layer); increased charge transport resistance under deformation; performance degradation caused by long-term bending	
Key points for performance optimization	Optimize the thickness of the light absorption layer and the contact area of the electrode interface to improve the carrier separation efficiency	Design stress-dispersing structures (such as porous electrodes) and develop self-healing electrolytes to reduce deformation damage to the photoelectric interface

**Table 2:** Advantages of light-assisted flexible energy storage devices over heat-assisted and electrical-assisted devices

Comparison dimensions	Light-assisted flexible energy storage device	Heat-assisted energy storage device	Electrical-assisted energy storage device
Energy source	Light energy directly participates in energy storage reactions, significantly improving efficiency	Dependence on fossil fuels (such as natural gas and coal) or thermal energy converted from electricity results in carbon emissions and is subject to fuel supply and transportation constraints	Depends on external power input, but requires frequent charging and incurs energy loss
Energy conversion efficiency	Short energy conversion path (direct storage of light energy → electrical energy), low intermediate loss	It requires multiple conversions from chemical energy to thermal energy to electrical energy, resulting in significant heat loss	There are transmission line losses and energy conversion losses during the charging process, resulting in low actual utilization rates
Flexibility and adaptability	Based on flexible electrode materials (such as nanofibers and composite films) and gel electrolytes, it can withstand bending, folding, and other deformations, making it suitable for wearable and curved devices	Heating modules and energy storage units are mostly rigid structures, and deformation can easily cause damage to components	Power supply lines and connectors are prone to poor contact during flexible deformation, making them difficult to adapt to flexible electronic scenarios
Application scenarios	Wearable electronics (such as health monitoring devices), IoT nodes, flexible displays, and portable energy systems	Mainly used in fixed scenarios (such as industrial energy storage and centralized heating energy storage), with limited outdoor and mobile applications	Dependent on the power grid, mainly used in indoor settings with stable power supply (such as consumer electronics and home energy storage)

## Working Principles of Photo-assisted Flexible Energy Storage Devices

Photo-assisted flexible energy storage devices achieve their unique functionality by integrating photoresponsive mechanisms with mechanically deformable architectures. This integration enables simultaneous light harvesting and energy storage even when the device is subjected to various deformation states such as bending, stretching, or twisting [28]. Unlike conventional energy storage systems that operate independently of illumination, or photovoltaic devices that solely convert light to electricity without inherent storage, these flexible systems leverage light-enhanced electrochemical processes [29]. This photo-assistance can significantly improve charging efficiency, reduce electrochemical overpotentials for reactions, and even enable self-charging capabilities, all while maintaining operational integrity during mechanical manipulation [30]. The operational principles are primarily governed by photoelectrochemical effects and photothermal effects, among which photoelectrochemical effects play a decisive role, while photothermal effects often coexist with photoelectrochemical effects, playing an auxiliary synergistic role, both of which must be understood and engineered to function reliably under the distinct mechanical deformation constraints and material interactions inherent to flexible systems.

### Mechanisms in Flexible Systems: Interplay of Light, Electrochemistry, and Strain

**Fig. 1:**
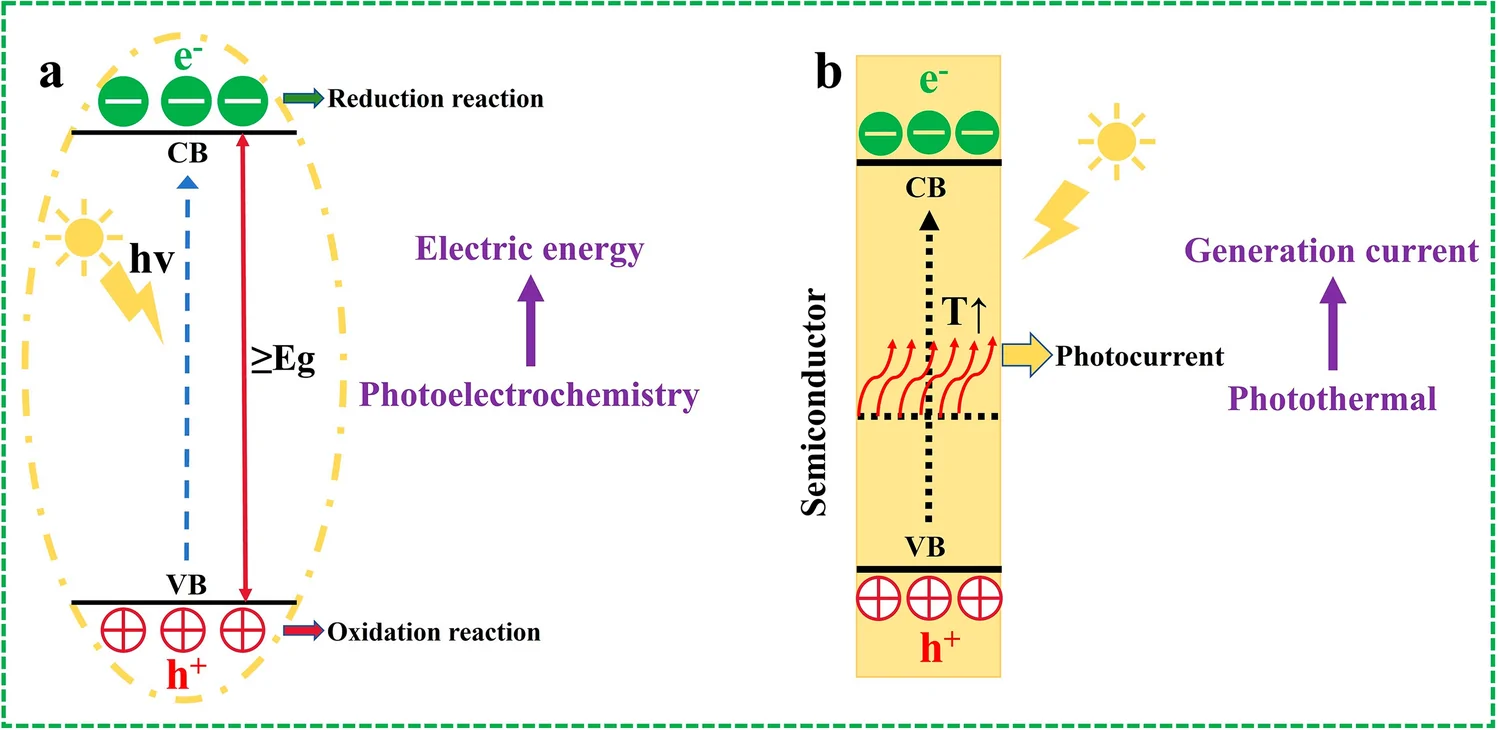
**a** Photoelectrochemical schematic diagram; **b** schematic diagram of photothermal effect

In flexible photo-assisted devices, the core photoelectrochemical process involves the absorption of photons by semiconductor materials (or other photoactive components like dyes or quantum dots) that are integrated with, or constitute part of, deformable electrodes [31]. This photon absorption, occurring when incident photon energy is equal to or exceeds the material’s bandgap energy (*E*_g_) or an equivalent electronic transition energy, generates electron–hole pairs (excitons). These charge carriers are then separated, typically by internal electric fields or at interfaces, and driven to participate in electrochemical redox reactions at the electrode–electrolyte interfaces, thereby converting light energy into stored chemical energy (Fig. 1a).

However, the introduction of mechanical flexibility and deformability adds significant complexity to these fundamental photoelectrochemical processes. Firstly, strain effects on electronic properties are notable, as mechanical strain from bending or stretching can directly alter the crystal lattice of semiconductor materials, leading to changes in their bandgap energy (piezo-phototronic effect), effective mass of charge carriers, and carrier mobility; tensile strain might narrow the bandgap and shift the absorption spectrum, while compressive strain could have an opposite effect, directly impacting light absorption efficiency and carrier energy [32]. For instance, the bandgap energy of unstrained Cs_2_SnI_6_ is approximately 1.257 eV. Under a compression strain of − 4%, the bandgap energy increases to 1.316 eV, while under a tensile strain of 4%, it decreases to 1.211 eV. This effect is primarily attributed to the displacement of the lowest energy level of the conduction band under compression and tensile strains. It was also found that both hole and electron carrier mobility decrease under tensile strain, while under − 4% compressive strain, electron carrier mobility increases by 16.3% and hole carrier mobility increases by 9.1% [33]. Secondly, ensuring the integrity of charge transport pathways under deformation is paramount because mechanical stress can induce micro-cracks in brittle photoactive layers, disrupt percolation networks in composite electrodes, or cause delamination from flexible current collectors, all of which increase internal resistance and degrade performance; mitigation strategies include intrinsically stretchable conductors, buckled/wrinkled electrode morphologies, or embedding active materials in elastomeric matrices [34]. Thirdly, the stability of electrode–electrolyte interfaces, where charge transfer occurs, can be compromised by mechanical deformation, as bending may cause localized detachment and stretching can alter electrode porosity, affecting electrolyte penetration, ionic diffusion, and interfacial integrity due to shear stresses [35]. Lastly, maintaining optical transparency and light penetration through various layers is challenging, as deformation can induce crazing, wrinkling, or delamination, leading to light scattering or opaque regions that reduce photon flux to the photoactive material.

Consequently, the efficiency of photoelectrochemical processes in flexible configurations depends not only on traditional factors (e.g., optimal bandgap alignment for redox potentials, efficient charge carrier separation and transport, fast interfacial charge transfer kinetics) but critically on how these factors are preserved or intentionally modulated under mechanical deformation. The integration of photoactive materials with flexible substrates thus introduces unique challenges for charge transport, charge collection, and interfacial stability that necessitate robust interface engineering, novel material composites, and architecturally resilient device designs to maintain photoelectrochemical activity across various deformation states and ensure long-term mechanical and electrochemical durability.

The photothermal effect often plays an auxiliary synergistic role. The photothermal effect in flexible photo-assisted devices arises from the conversion of absorbed photon energy into localized thermal energy [36]. This occurs primarily through non-radiative recombination processes of photogenerated carriers within the photoactive material or through direct absorption by light-absorbing (but not necessarily photoelectrochemically active) components [37]. This localized temperature increase can provide additional activation energy for electrochemical reactions, thereby improving reaction kinetics, reducing overpotentials, and enhancing ionic conductivity within the electrolyte, especially in polymer-based or solid-state electrolytes commonly used in flexible devices (Fig. 1b) [38].

Therefore, while the photothermal effect can be harnessed to boost performance, careful thermal management and design considerations are critical in flexible systems. This includes selecting materials with appropriate thermal conductivities and expansion coefficients, designing structures that can accommodate thermal stresses, and ensuring that localized heating does not lead to the degradation of flexible components or compromise mechanical integrity during deformation. The synergistic combination of precisely controlled photoelectrochemical and photothermal effects within mechanically adaptive architectures remains a promising avenue for enhancing the performance of flexible photo-assisted devices under ambient light conditions, provided these mechanical and thermal interplay challenges are adequately addressed.

In light-assisted flexible energy storage devices, photochemical reactions serve as the core driving mechanism. Photoreactive materials absorb photons to generate photogenerated carriers, which are then separated and transported to directly participate in electrode redox reactions or charge storage processes. This “light-to-electricity” direct coupling provides an efficient pathway to enhance the device's energy storage capacity and conversion efficiency, making it the core driving force for achieving light-assisted functionality; meanwhile, the photothermal effect plays a supplementary coordinating role. By converting light energy into thermal energy, it indirectly optimizes the diffusion rate of electrolyte ions and the reactivity of electrode reactions. Additionally, it moderately alleviates stress concentration during deformation of the flexible substrate, thereby protecting the core carrier transport channels of the photochemical process. However, it is important to note that the photothermal effect cannot independently achieve efficient energy storage, and excessive heating may lead to material degradation or accelerated carrier recombination, thereby weakening the dominant photochemical effect. Although both effects synergistically participate in device performance regulation, the dominant role of photochemical processes is clear, while the photothermal effect serves as a supplementary mechanism. Through precise regulation, the overall efficiency can be further enhanced.

### Mechanical–Electrochemical Coupling Effects: A Defining Feature of Flexible Systems

A distinctive and foundational feature of flexible photo-assisted energy storage devices is the profound coupling between mechanical deformation and their electrochemical and photoresponsive performance. This is not merely a parasitic effect to be mitigated but a core aspect that must be understood and potentially engineered. Bending, stretching, twisting, or compressing the device can directly and significantly influence:

1. Photon Absorption Efficiency: Mechanical deformation can induce changes in surface morphology. For example, stretching can flatten wrinkled surfaces, while compression or bending can create folds or buckles. These topographical changes can alter the angle of incident light, induce light scattering effects, or even change the effective optical path length within the photoactive material, thereby influencing the overall photon harvesting efficiency [39]. In some architected designs, controlled wrinkling can even be used to enhance light trapping [40].
2. Charge Transport Pathways: As discussed earlier, strain can open or close micro-cracks, alter the contact between conductive particles in a composite electrode, or change the tortuosity of ion diffusion paths within the electrode and electrolyte. For instance, tensile strain might align conductive nanofillers in an elastomer matrix, potentially improving conductivity along the strain direction but reducing it perpendicularly [41].
3. Electrolyte Distribution and Ion Transport: Mechanical compression can squeeze electrolyte out of porous electrode structures, while stretching might increase void volume. These changes directly impact the wetted surface area of the electrode, ionic conductivity within the pores, and the overall concentration gradients of reactive species, thereby affecting reaction rates and device impedance [42, 43].
4. Electrode Kinetics and Interfacial Capacitance: Strain can modify the active surface area of electrodes and alter the energetics of adsorption/desorption processes or charge transfer reactions at the electrode–electrolyte interface, thereby affecting both Faradaic reaction rates and capacitive charge storage [44, 45].

Understanding these intricate mechanical–electrochemical and mechanical–photo-optical interactions is crucial for designing robust and reliable flexible devices for applications like wearable electronics, where devices are constantly subjected to dynamic and unpredictable mechanical stimuli. The preservation of, or even the designed modulation of, photoelectrochemical functionality under mechanical stress requires specialized electrode and device designs. This includes, but is not limited to, strategies such as employing intrinsically stretchable materials, designing electrodes with pre-defined buckled, kirigami, or serpentine structures that can accommodate large strains without damaging the active components, or embedding photoactive particles within highly elastic and ionically conductive matrices. This inherent integration challenge, and the opportunity it presents for novel device functionalities, fundamentally distinguishes flexible photo-assisted systems from their rigid counterparts and necessitates innovative and multidisciplinary approaches to material selection, interface engineering, and device architecture design.

## Electrode Materials for Flexible Photo-Assisted Energy Storage

This section presents a comprehensive analysis of electrode materials utilized in photo-assisted energy storage devices, with a specific emphasis on their suitability and adaptation for flexible applications. It is followed by a detailed examination of optimization strategies designed to enhance their overall photoelectric performance, particularly under conditions of mechanical stress that are inherent to flexible systems. The light response characteristics of the relevant materials are shown in Table 3. The mechanical properties of the relevant materials are shown in Table 4. The systematic investigation encompasses various material categories, their fundamental properties, and specific modifications aimed at improving device efficiency and mechanical resilience. Through careful consideration of structure–property relationships, mechanical integrity, and performance optimization methods, this analysis provides crucial insights into the development of advanced electrode materials for flexible photo-assisted energy storage.

**Table 3:** Light response characteristics of relevant photocatalysts

Materials	Bandgap (eV)	Light absorption range (nm)	Application	R_ct_ (Ω)	Photocurrent density	Stability	References
GdVO_4_	2.2	390–770	SC	4.2			[46]
Ce-doped V_2_O_5_	2.23	420–480	SC	0.48		95.2% (5000 cycles)	[47]
GO-NiO	2.77	200–700	SC			98% (6000 cycles)	[48]
Cu_2_O/MX_n_	1.868	300–800	SC			91.01% (5000 cycles)	[49]
Fe_5%_NiO	3.21	375–425	SC				[50]
MoS_2_/Graphene	1.8	200–700	Li–O_2_ battery				[51]
Ag@ZnO	3.11	370–550	Li–O_2_ battery		~ 0.042 mA cm^−2^	93.73% (CE)	[52]
NiFe-TiO_2_	2.73	200–800	Zn-air battery	6		60 cycles (30h)	[53]
CoS_2_/CuS@CNT-C_3_N_4_		420–800	Zn-air battery		~ 0.016 mA cm^−2^	3800 cycles (643h)	[54]
Co_3_O_4_	2.20 (Direct) 1.35 (Indirect)	400–755	Zn-air battery	11.1			[55]
Bi_2_O_3_/TiO_2_	3.00	250–452		~ 3250	0.56 mA cm^−2^		[56]
Cu_2_O/WO_3_/TiO_2_	2.35	415–515	SC	208.10	1.40 mA cm^−2^	95.17% (1000 cycles)	[57]
MoS_2_/TiO_2_	3.19			~ 1800			[58]
CTH/MoS_2_-TAA	1.90 (3D MoS_2_)	350–750	SC	0.1		97.5% (10,000 cycles)	[59]
6% Fe-doped CeO_2_	2.43		SC			99.36% (100 cycles)	[60]
Al–MnO_2_	1.40		Zn-ion battery	75.7	30 mA	96% (1000 cycles)	[61]
CsPbI_1.8_Br_1.2_	1.66		Battery	0.62			[62]

CE means cycle efficiency

**Table 4:** Mechanical properties of related electrode materials

Electrode material	Application	Young's modulus	Tensile strength (MPa)	Final strain (elongation)	Max bending (angle/radius)	Capacitance	Energy density	Bending cycle performance	References
CC/MoS_2_@C	SC				175°	108.3 mF cm^−2^		113% (5000 bends)	[63]
V_2_O_5_-rGO	SC	1.7 GPa	6.1	3%	145°	511.7 mF cm^−2^	89 μW h cm^−2^		[64]
PANI-PPG	SC	134.9 ± 34.3 kPa		608.2%	Any angle	95.8 mF cm^−2^	8.5 μWh cm^−2^	> 90% (1000 bends)	[65]
MXene-cellulose paper-MXene	SC				0.6 cm	77.25 mF cm^−2^			[65]
EG-PCPPH//CCPH-PPy	SC		22.3	286%	180°		397.99 μWh cm^−3^	98% (1000 bends)	[66]
MXene/Au NPs	SC				180°	332 F g^−1^	1.35 μWh cm^−2^		[67]
LCO/PVDF/CNT	Battery	1.3 GPa			3 mm				[68]
LCO/graphite	Li-ion battery	70 MPa			180°	154.5 mAh g^−1^	242.21 Wh L^−1^	97.28% (5000 bends)	[69]
PDHBQ S-SWCNTs	Li-ion battery	2.8 GPa	20.1	2.5%	2.1 cm	182 mAh g^−1^		88% (2000 bends)	[70]
PMTA/SWCNT	Li-ion battery	10.6 MPa	0.44	9.33%	0.67 cm	163 mAh g^−1^		80% (1000 bends)	[71]
GCF/Cu/Li	Lithium Metal Full Battery			2.5%	< 2 mm		300 Wh kg^−1^	> 95% (500 bends)	[72]
MnO_2_ NWs/SWNT/FLGs	Zn-ion battery		25	~ 4.9%	180°/1 mm	374 mAh g^−1^	651.5 Wh kg^−1^	91.71% (75 bends)	[73]

### Metal Oxides and Sulfides for Flexible Systems

#### Metal Oxides in Flexible Photo-Assisted Devices

Metal oxides represent a fundamental class of photoactive materials for flexible photo-assisted energy storage devices, offering tunable optoelectronic properties, chemical stability, and diverse structural configurations suitable for mechanical adaptation [74]. However, the inherent brittleness of crystalline metal oxides presents significant challenges for flexible applications, necessitating innovative material engineering strategies including nanostructure design, composite formation, and strain-tolerant integration with flexible substrates to maintain both photoelectrochemical functionality and mechanical resilience under deformation.

1. Single Metal Oxide Systems and Flexible Integration Strategies

Titanium dioxide (TiO_2_) has emerged as a cornerstone photoactive material for flexible energy storage applications due to its exceptional chemical stability, non-toxicity, photocorrosion resistance, and compatibility with flexible substrate integration [75, 76]. For flexible implementations, TiO_2_ nanostructures are strategically integrated with flexible conductive substrates including carbon cloth, carbon fiber, and polymer films to create mechanically adaptable photoelectrodes that maintain performance under deformation. However, practical applications remain constrained by limited visible light absorption and photoconversion efficiency attributed to wide bandgap characteristics, requiring innovative approaches to enhance solar energy utilization while preserving mechanical flexibility.

**Fig. 2:**
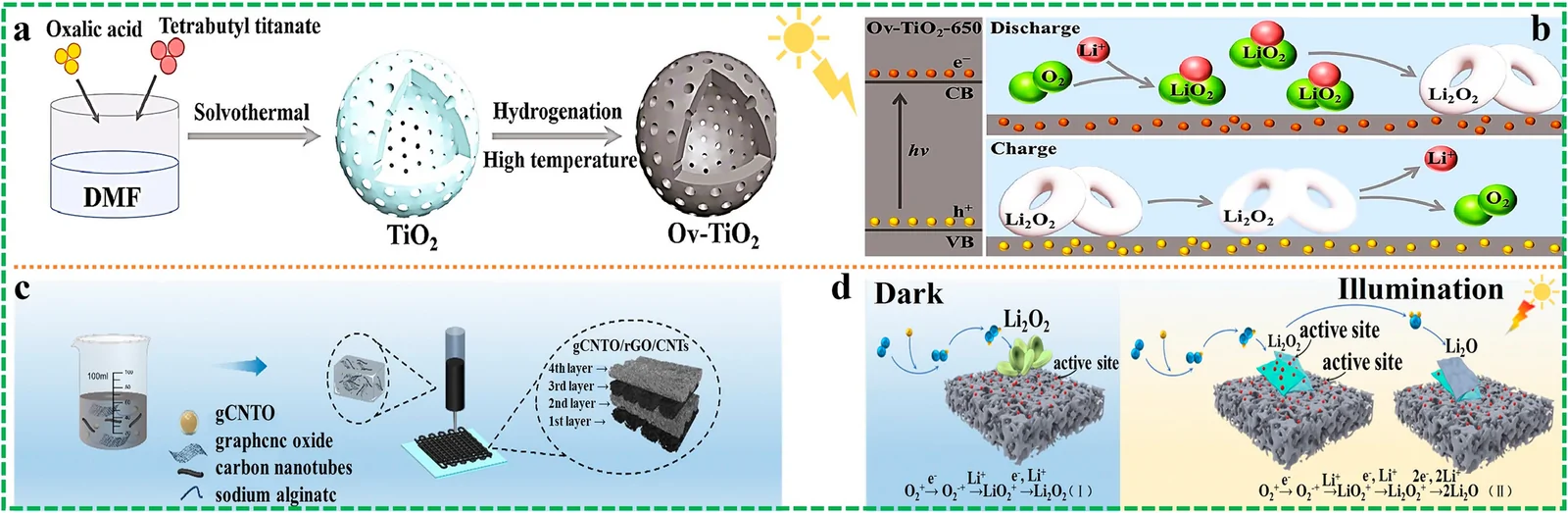
**a** Schematic illustration of the preparation process of Ov–TiO_2_–T (T = 550, 600, 650 and 700). **b** Schematic diagrams of the reaction mechanisms for the photo-assisted Li–O_2_ batteries with Ov–TiO_2_-650. Reproduced with permission [78]. Copyright 2022, Elsevier. **c** Schematic illustrating the process of making ink and 3D printed air cathode for Li–O_2_ batteries. **d** Schematic diagram of the discharge mechanisms for photo-assisted Li–O_2_ batteries with the gCNTO/rGO/CNTs photocathode. Reproduced with permission [79]. Copyright 2024, Royal Society of Chemistry

Advanced oxygen vacancy engineering in TiO_2_ systems demonstrates significant potential for flexible photo-assisted lithium-ion battery applications through enhanced solar responsiveness and photocatalytic activity [77, 78]. The introduction of oxygen vacancies substantially enhances TiO_2_’s photoelectrochemical properties, although excessive vacancy concentrations can potentially serve as recombination centers for photogenerated electron–hole pairs, requiring precise control for optimal performance. Oxygen-vacant TiO_2_ hollow nanospheres (O_v_-TiO_2_) coated onto flexible carbon paper substrates create mechanically adaptable cathode architectures where oxygen vacancies serve as charge separation centers under illumination (Fig. 2a) [78]. The photoelectrochemical mechanism in flexible O_v_-TiO_2_ systems involves strategic charge carrier utilization during electrochemical processes. Under illumination, oxygen vacancies effectively enhance electron–hole separation, where photogenerated electrons promote the reduction of O_2_ to Li_2_O during the oxygen reduction reaction (ORR), while photogenerated holes facilitate the decomposition of Li_2_O during the oxygen evolution reaction (OER) (Fig. 2b). This dual-pathway mechanism maintains efficiency even under mechanical deformation, with the optimized O_v_-TiO_2_-650 flexible photoelectrode demonstrating reduced overpotentials (0.70 V) and excellent rate capabilities while preserving structural integrity under repeated flexing cycles.

Three-dimensional hierarchically porous architectures incorporating g-C_3_N_4_@TiO_2_ catalyst composites with reduced graphene oxide and carbon nanotubes (gCNTO/rGO/CNTs) represent breakthrough flexible photoelectrode designs that address both mechanical adaptability and electrochemical performance requirements (Fig. 2c) [79]. The mechanism advantage of these structured flexible cathodes lies in the fact that, even when covered by an amorphous Li_2_O_2_ film during operation, they can still maintain active sites for the conversion of Li_2_O_2_ to Li_2_O under illumination, thereby sustaining their photocatalytic activity under mechanical stress (Fig. 2d). Performance analysis reveals remarkable enhancement mechanisms, with flexible cathodes delivering discharge areal capacities of 29.73 mAh cm^−2^ under illumination compared to 18.16 mAh cm^−2^ in dark conditions, translating to energy densities of 515.12 Wh kg^−1^ under illumination versus 298.72 Wh kg^−1^ in darkness. The hierarchical porous structure accommodates mechanical stress while preserving photoelectrochemical interfaces through enhanced charge separation and transport efficiency, establishing viable pathways for high-performance flexible lithium-ion battery implementation.

**Fig. 3:**
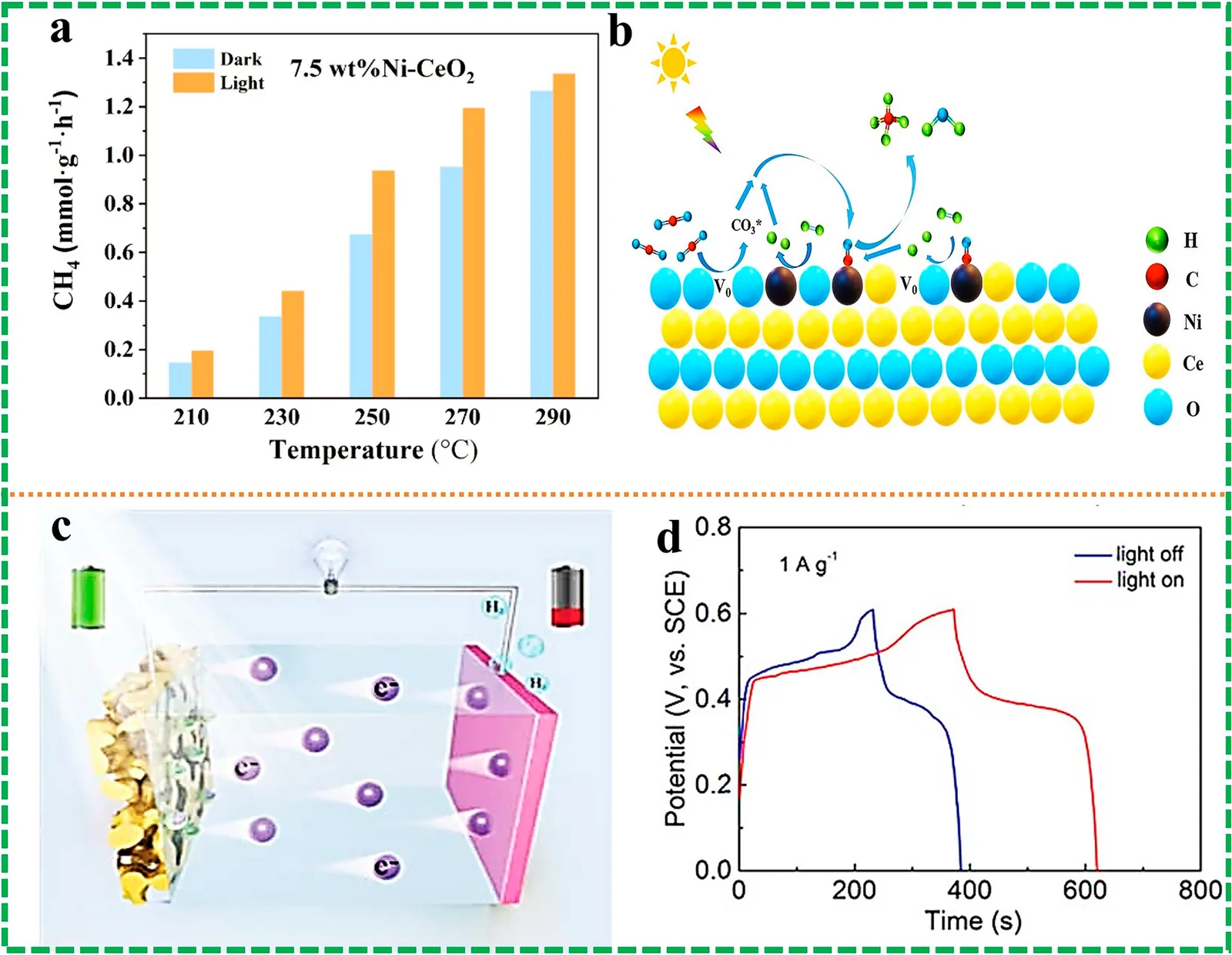
**a** Performance of 7.5 wt.% Ni–CeO_2_. **b** Proposed mechanism of the CO_2_ methanation reaction. Reproduced with permission [82]. Copyright 2023, Elsevier. **c** Schematic representation of the electrode reactions in SC with/without light illumination. **d** Galvanostatic charge–discharge profiles at 1 A g^−1^ under dark condition and light illumination. Reproduced with permission [12]. Copyright 2019, Royal Society of Chemistry

Cerium dioxide (CeO_2_) exhibits distinctive photoelectrochemical properties particularly suitable for flexible applications, including visible light absorption capabilities and valence state flexibility through Ce^3+^ and Ce^4+^ transitions under illumination [80, 81]. This valence state flexibility enables visible light absorption and provides fundamental mechanisms for photoelectric conversion, effectively utilizing solar energy resources while maintaining functionality under mechanical stress. Strategic nickel doping fundamentally alters CeO_2_’s physicochemical properties through several interconnected mechanisms. Ni dopant atoms increase surface oxygen vacancies, modify the band structure, enhance CO_2_ activation, and optimize both optical characteristics and electronic configuration [82]. The optimized 7.5 wt.% Ni–CeO_2_ system demonstrates superior visible-light-driven performance, achieving 39% enhancement in catalytic activity from 0.67 to 0.93 mmol g^−1^ h^−1^ at 250 °C (Fig. 3a). Mechanistic investigations reveal that Ni doping increases oxygen vacancy concentration, reduces bandgap width, enhances electron density, and accelerates intermediate conversion processes. Furthermore, visible-light-induced oxygen vacancy regeneration provides additional active sites, creating synergistic promotional effects (Fig. 3b). This mechanistic understanding establishes design principles for flexible photoelectrochemical systems requiring enhanced visible light utilization.

Cuprous oxide (Cu_2_O) has emerged as a promising photoelectric material due to its remarkable visible light responsiveness and superior carrier mobility characteristics. However, its practical implementation in photo-assisted energy storage devices has been constrained by high carrier recombination rates and limited surface reactive sites, resulting in suboptimal photoelectric conversion efficiency [83]. Addressing these limitations, Liu et al. [12] have developed an innovative integrated system combining photoelectrode and working electrode functionalities through the fabrication of nanoporous Cu@Cu_2_O (NPC@Cu_2_O) hybrid array electrodes, successfully demonstrating enhanced photo-assisted SC performance (Fig. 3c). This advanced nano-porous/array hybrid architecture not only ensures efficient operation under illumination but significantly improves solar energy utilization efficiency. Under experimental conditions at a current density of 1 A g^−1^, the system achieves an impressive specific capacitance of 782 F g^−1^ under illumination, representing a 37.9% enhancement compared to dark conditions (Fig. 3d), while maintaining excellent cycling stability under illumination. The mechanism involves photogenerated holes at the Cu_2_O surface activating additional reactive sites, facilitating proton insertion into the Cu_2_O surface structure, thereby establishing a novel pathway for direct solar energy storage. This architectural design and mechanistic understanding provide a promising approach for integrating solar energy conversion and storage capabilities in next-generation energy devices.

Single-metal oxide photonic-assisted flexible energy storage systems utilize core photoresponsive materials such as TiO_2_ and Cu_2_O, leveraging their semiconductor properties and compatibility with flexible substrates to demonstrate unique value in the field of flexible energy storage. These materials exhibit excellent light absorption properties and have mature preparation processes, making them easy to integrate with flexible substrates such as carbon cloth and titanium foil to meet the requirements of flexible devices. Their light-assisted mechanism primarily relies on the separation and transport of photo-generated charge carriers (electron–hole pairs), enhancing energy storage performance by promoting redox reactions at the electrode surface (e.g., ORR/OER, ion intercalation/deintercalation). However, this system has inherent limitations: The wide bandgap of single metal oxides results in low spectral utilization efficiency, high carrier recombination rates limit photoconversion efficiency, and the mechanical brittleness of single oxides makes them prone to structural damage after repeated bending. To address these issues, research often employs strategies such as doping modification, morphology control, or composite formation with conductive polymers. Overall, single metal oxide systems offer a low-cost, scalable solution for light-assisted flexible energy storage. However, further improvements in performance require a better balance between light absorption range and mechanical stability. In the future, multi-dimensional regulation is expected to enable wider application in fields such as wearable devices.

1. Multielement Metal Oxide Systems for Enhanced Flexible Performance

Given the inherent limitations of single metal oxide systems, including high costs, low conductivity, and mechanical brittleness, the development of multielement mixed metal oxide electrodes represents a strategic advancement in flexible photo-assisted energy storage devices. Multielement metal oxide compositions combining multiple distinct transition metals demonstrate synergistic effects that significantly enhance material conductivity, specific capacitance, electrochemical stability, mechanical durability, and cycling performance essential for flexible energy storage implementations. These improvements arise from complementary electronic structures, enhanced charge carrier mobility, optimized band alignments, and improved mechanical properties that collectively enable superior performance in mechanically dynamic environments. Strategic multielement combinations including nickel-manganese oxide, manganese-cobalt oxide, manganese-iron oxide, copper-cobalt oxide, copper-nickel oxide, and the systems discussed below have demonstrated exceptional performance characteristics that prove particularly advantageous for flexible applications where maintaining electrochemical activity under mechanical stress is critical [84–90].

**Fig. 4:**
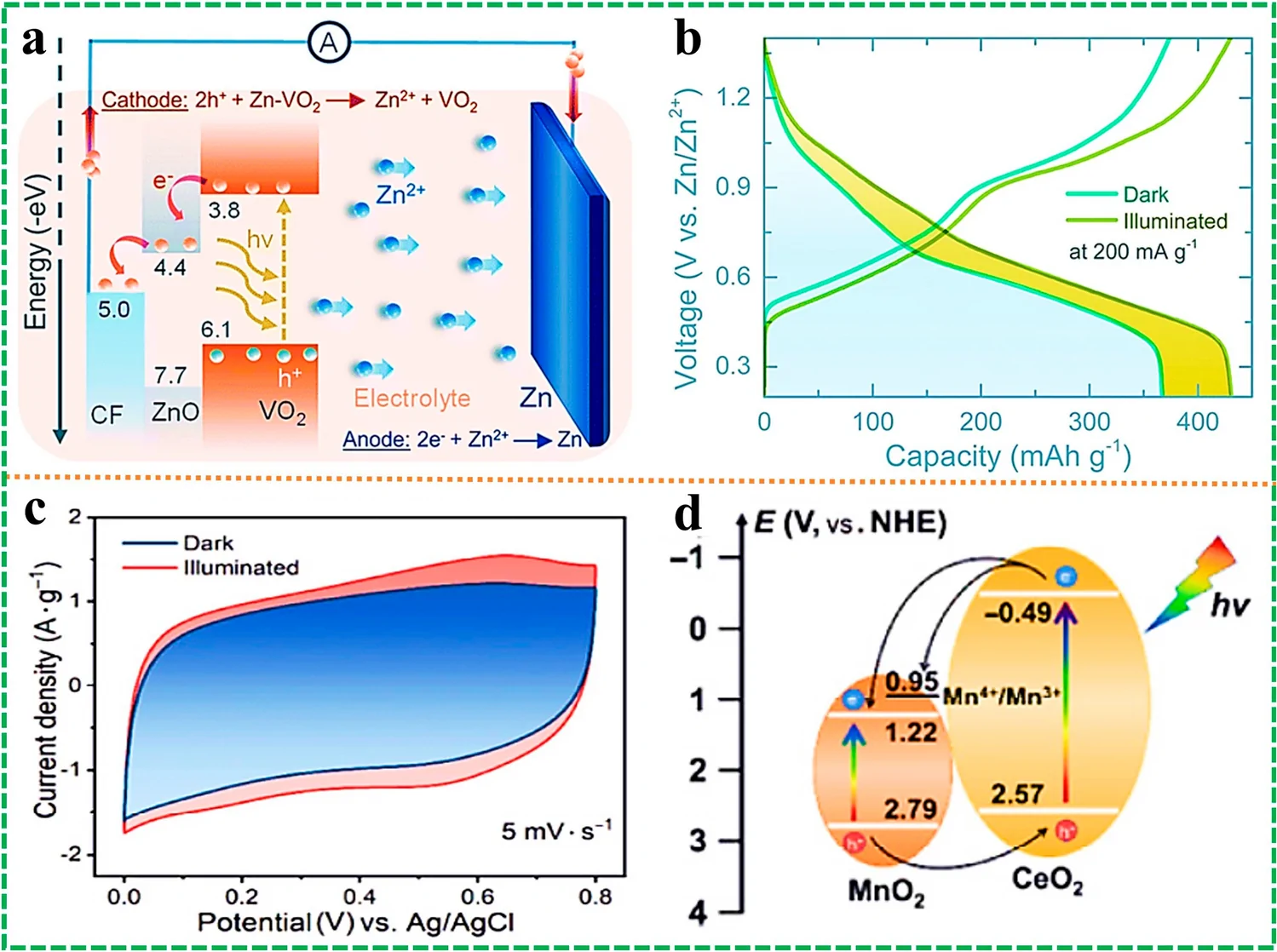
**a** Schematic illustration of photocharging mechanism of the proposed VO_2_/ZnO Zn-ion photo-battery. **b** GDC curves of the photo-batteries at 200 mA g^−1^ both in dark and illuminated conditions (~ 455 nm and intensity 12 mW cm^−2^) after one formation cycle. Reproduced with permission [91]. Copyright 2021, Royal Society of Chemistry. **c** CV curves of CeO_2_/MnO_2_-CFP electrodes in dark and under visible light illumination (400–780 nm) at the scan rate of 5 mV s^−1^. **d** Band structure diagram of the CeO_2_/MnO_2_ heterojunction. Reproduced with permission [81]. Copyright 2022, Tsinghua University Press

The VO_2_/ZnO binary metal oxide system demonstrates exceptional promise for flexible zinc-ion battery applications through innovative architectural designs that combine photoelectrochemical enhancement with mechanical adaptability [91]. Advanced flexible electrode architectures incorporate ZnO layers on carbon fiber substrates as hole-blocking and electron-transport layers, followed by direct VO_2_ growth to create strain-tolerant binary photoelectrode structures. The engineered VO_2_/ZnO flexible binary architecture exhibits a distinctive photo-charging mechanism that maintains efficiency under mechanical deformation, demonstrating the advantages of combining two metal oxides with complementary electronic properties. Under illumination, photoexcited electrons migrate from the valence band of VO_2_ to its conduction band, subsequently traversing the ZnO layer to reach the carbon fiber collector. Simultaneously, photogenerated holes are effectively blocked by the ZnO layer, preventing recombination and enhancing charge separation efficiency through the synergistic interaction between the two metal oxide components (Fig. 4a). This binary architectural design achieves remarkable improvements: 2.8-fold enhancement in photoelectric conversion efficiency (from 0.18% to 0.51%) compared to conventional mixed electrodes, delivering impressive specific capacities of 432 mAh g^−1^ under illumination versus 367 mAh g^−1^ in dark conditions (Fig. 4b). The binary system maintains 73% capacity retention after 500 cycles while reducing charging time by two-thirds, representing significant advancement in flexible zinc-ion photobattery technology. Critical challenges for flexible implementations include ensuring both ZnO and VO_2_ layer integrity under repeated deformation, maintaining the cooperative hole-blocking and electron-transport functionalities across various bending states, and preventing interface degradation between the two metal oxide components during mechanical cycling.

Advanced CeO_2_/MnO_2_ binary composite systems integrated onto flexible carbon fiber paper substrates demonstrate remarkable potential through type-II heterojunction formation mechanisms [81]. The strategic modification with minimal CeO_2_ nanoparticles significantly enhances photo-assisted charging capabilities through formation of type-II CeO_2_/MnO_2_ heterojunctions that facilitate effective photogenerated carrier separation and transfer between the two metal oxide components, resulting in approximately 22% improvement in photo-assisted charging capacity (Fig. 4c). The engineered binary band structure enables controlled photoelectron release while effectively inhibiting dark reactions with oxygen (Fig. 4d), allowing the material to retain approximately 56% of its photo-assisted capacitance even after 12 h in darkness. This retention mechanism demonstrates the binary system’s ability to store photoelectrons and gradually release them through cooperative interactions between CeO_2_ and MnO_2_, establishing fundamental design principles for flexible supercapacitors (SCs) with extended dark-state retention capabilities through binary metal oxide synergy.

**Fig. 5:**
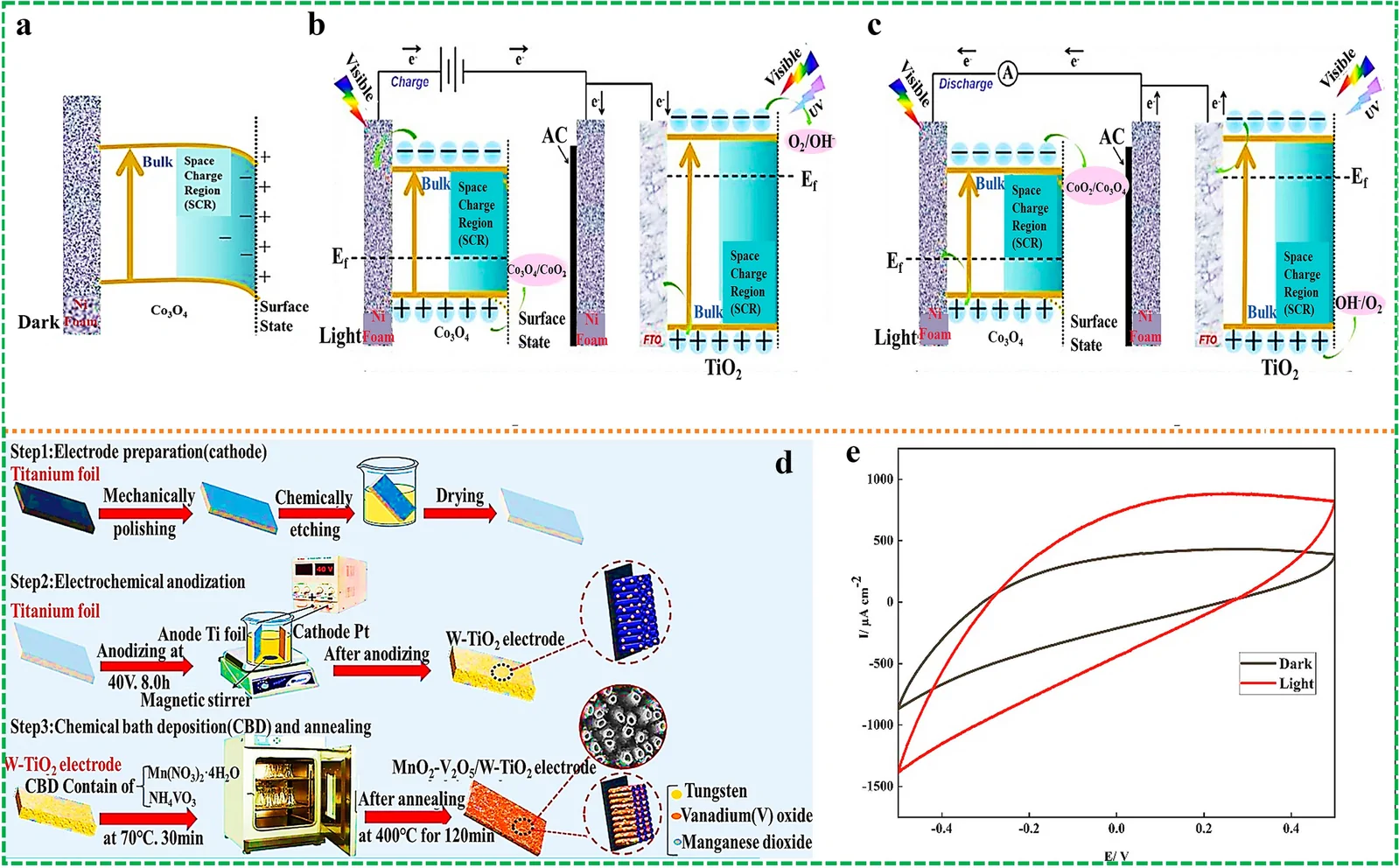
Schematic of **a** Co_3_O_4_ under dark. Mechanism of the whole TiO_2_ photo-assisted system under light for **b** charging process and **c** discharging process. Under light irradiation, the semiconductor Co_3_O_4_ generates electron–hole pairs, and then the band bending can be reduced due to photogenerated charge compensation. After continuous irradiation, excess photogenerated holes will be oxidized by Co_3_O_4_ to generate CoOOH and CoO_2_. Reproduced with permission [94]. Copyright 2022, Elsevier. **d** Schematic illustration for deposition of MnO_2_ and V_2_O_5_ on WTNTS and preparation of MnO_2_–V_2_O_5_ co-deposited electrode. **e** CVs of sample carried out under dark and light illumination. Reproduced with permission [95]. Copyright 2022, Elsevier

Cobalt oxide (Co_3_O_4_) [92, 93] integration with TiO_2_ in flexible asymmetric SC systems demonstrates sophisticated photoelectrochemical enhancement mechanisms through strategic coupling of two complementary metal oxides [94]. Flexible architectures incorporating Co_3_O_4_ nanospheres on flexible nickel foam substrates with TiO_2_ auxiliary photoelectrodes achieve specific capacitance improvements from 450 to 523 F g^−1^ (16.7% enhancement) while maintaining mechanical flexibility. Co_3_O_4_ is a p-type semiconductor, and downward band bending may occur at the electrode/electrolyte interface (Fig. 5a). The complex binary photoelectrochemical mechanism involves coordinated light absorption and charge utilization across different wavelength ranges, demonstrating the advantages of combining two metal oxides with complementary optical properties (Fig. 5b, c). During charging, TiO_2_ predominantly absorbs UV light, while Co_3_O_4_ captures visible light, generating photoinduced charges that promote the charging process. On the TiO_2_ film, photogenerated holes recombine with electrons from the power supply’s negative terminal, while photogenerated electrons participate in oxygen reduction reactions. During discharge, TiO_2_’s photogenerated holes engage in OH^−^ oxidation reactions, while photogenerated electrons are injected into the external circuit and recombine with photogenerated holes in Co_3_O_4_. Subsequently, photogenerated electrons in Co_3_O_4_ reduce CoO_2_ to Co_3_O_4_, completing the photoelectrochemical cycle. This mechanistic understanding validates the synergistic role of both metal oxides in enhancing both charging and discharge processes, significantly improving flexible energy storage performance through binary metal oxide cooperation.

Building upon the established advantages of multielement metal oxide systems, innovative flexible photoelectrode architectures have been developed utilizing chemical bath deposition (CBD) techniques to co-deposit manganese dioxide (MnO_2_) and vanadium pentoxide (V_2_O_5_) on flexible tungsten titanate nanotube films (WTNTS), creating mechanically adaptable photoelectrochemical cell-type SCs for direct solar energy storage (Fig. 5d) [95]. The MnO_2_–V_2_O_5_/WTNTS binary photoelectrode system operates through sophisticated photoelectrochemical mechanisms that maintain efficiency under mechanical deformation, where the WTNTS substrate functions as the photoactive center, absorbing solar energy to generate electron–hole pairs that are subsequently utilized by the binary metal oxide composite layer for enhanced energy storage. Under illumination, photogenerated holes migrate to and are stored within the capacitive MnO_2_–V_2_O_5_ composite layer, while photogenerated electrons participate in complementary electrochemical reactions that enhance overall charge storage capacity through synergistic effects created by complementary electrochemical activities: MnO_2_ provides pseudocapacitive behavior through surface redox reactions involving Mn^3+^/Mn^4+^ transitions, while V_2_O_5_ contributes through multiple oxidation states (V^3+^/V^4+^/V^5+^) that create overlapping potential windows and multiple electron transfer pathways. This mechanistic advantage results in remarkable performance enhancement, with the co-deposited flexible electrode demonstrating specific capacitance values 4.1 and 3.6 times higher than electrodes with individual MnO_2_ and V_2_O_5_ deposits, respectively, while area capacitance increases from 38 to 95 mF cm^−2^ (150% enhancement) under illumination (Fig. 5e). The optimized system exhibits excellent reversibility and cycling stability, maintaining 94% capacitance retention after 5000 cycles, with exceptional stability arising from the multielement metal oxide composition providing structural reinforcement and multiple electrochemical pathways.

The successful implementation of multielement metal oxides in flexible photo-assisted energy storage devices requires systematic consideration of several critical design principles: (1) Complementary electronic structure design ensuring that multielement metal oxide components provide synergistic rather than competing photoelectrochemical activities; (2) Strain-tolerant interface engineering that preserves charge carrier separation and transport pathways between metal oxide components under deformation; (3) Mechanical compatibility optimization requiring similar mechanical properties and thermal expansion coefficients to prevent interface failure under mechanical stress; (4) Electronic coupling enhancement ensuring efficient charge transfer between multielement metal oxide components and with flexible substrates; (5) Structural integrity maintenance under combined electrochemical and mechanical cycling of both metal oxide phases; and (6) Processing compatibility with flexible substrate requirements and large-scale manufacturing processes for multielement systems.

These principles guide rational design of next-generation flexible photo-assisted energy storage systems that effectively utilize multielement metal oxide photoelectrochemical synergies while meeting mechanical adaptability requirements essential for wearable and conformable energy applications. The successful demonstration of VO_2_/ZnO [91], CeO_2_/MnO_2_ [81], Co_3_O_4_/TiO_2_ [94], MnO_2_–V_2_O_5_ [95], and multielement systems establishes fundamental design paradigms for developing high-performance flexible photo-assisted energy storage devices and extends to other strategic multielement metal oxide combinations suitable for flexible energy storage applications.

#### Metal Sulfides in Flexible Photo-Assisted Devices

1. Single Metal Sulfide Systems and Flexible Integration Strategies

Metal sulfides represent a complementary class of photoactive materials to metal oxides for flexible photo-assisted energy storage devices, offering distinct advantages including tunable bandgap characteristics, exceptional chemical stability, superior photostability, and abundant active sites for electrochemical reactions [96]. However, their practical implementation in flexible photo-assisted energy storage faces multifaceted challenges that stem from both intrinsic material limitations and mechanical constraints essential for flexible device operation. The intrinsic limitations include low carrier mobility, which substantially impacts photoelectric conversion efficiency and reaction kinetics, and high recombination rates of photogenerated charges that diminish utilization efficiency. More critically for flexible applications, metal sulfides exhibit inherent brittleness that compromises mechanical adaptability, necessitating targeted optimization strategies encompassing structural modification, interface engineering, compositional tuning, and flexible substrate integration to achieve both photoelectrochemical performance and mechanical resilience under deformation.

Molybdenum disulfide (MoS_2_) has garnered significant attention as a promising photoactive material for flexible energy storage applications due to its exceptional photoelectric properties, chemical stability, and versatile catalytic performance for various electrochemical reactions, including hydrogen evolution reaction (HER), oxygen reduction reaction (ORR), and oxygen evolution reaction (OER) [97, 98]. The catalytic performance of MoS_2_ can be strategically enhanced through precise engineering of edge sites, defects, doping states, and phase structures, making it particularly suitable for photoelectrochemical applications requiring controlled reactivity and stability. However, traditional 2D MoS_2_ nanosheets face fundamental limitations for flexible photoelectrochemical applications. Only unsaturated sulfur atoms at the edges of 2D MoS_2_ nanosheets serve as effective catalytic centers, which inherently limits photogenerated carrier separation efficiency and overall catalytic performance [99]. Additionally, the mechanical brittleness of crystalline MoS_2_ structures presents significant challenges for maintaining photoelectrochemical activity under mechanical deformation, requiring innovative architectural approaches that preserve both catalytic functionality and structural integrity in flexible configurations.

**Fig. 6:**
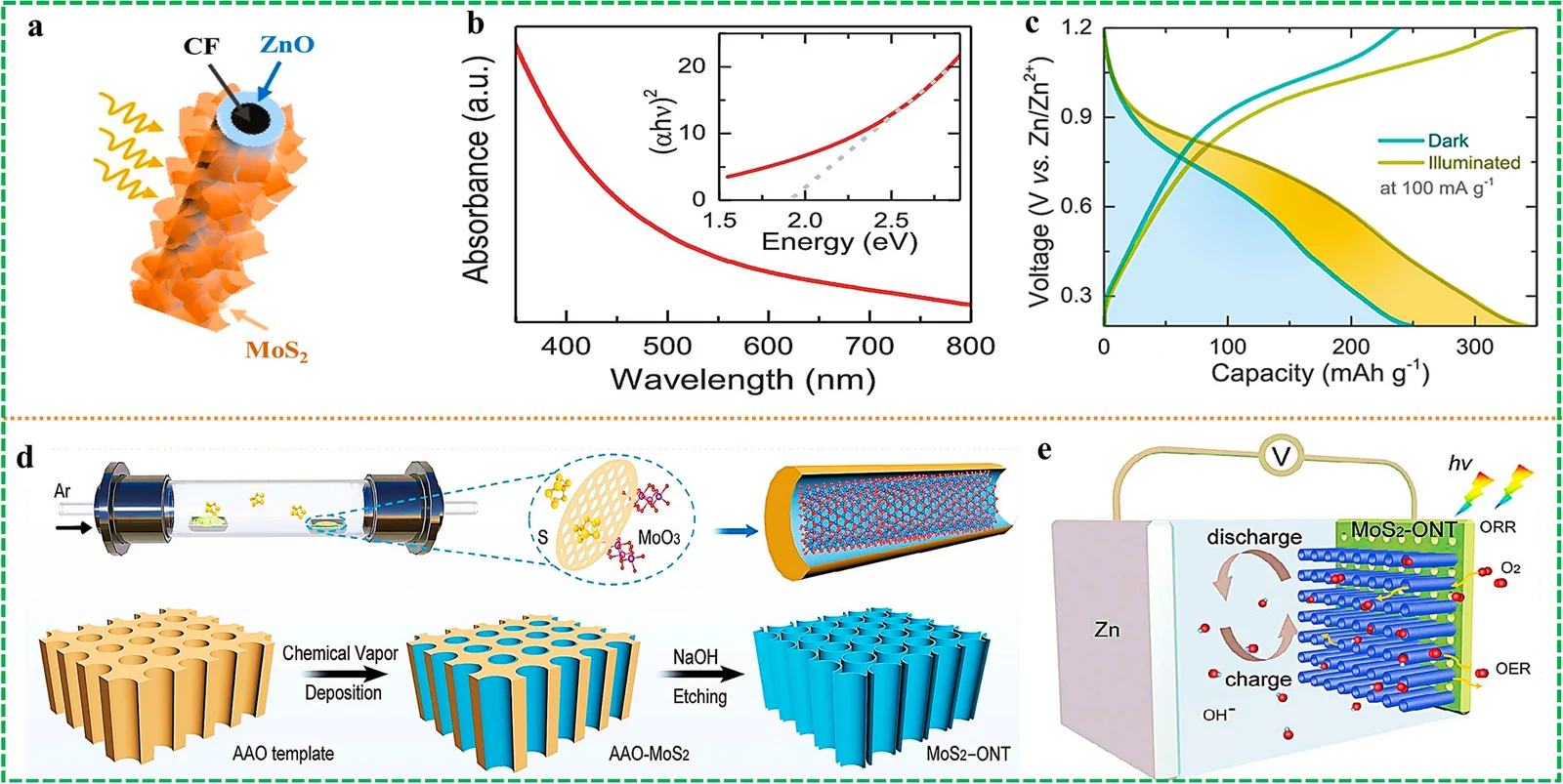
**a** Schematic illustration of MoS_2_ nanosheets grown on a ZnO-coated carbon fiber. **b** UV − Vis absorption spectrum and Tauc plot of the as-grown 2D MoS_2_ nanosheets. **c** Galvanostatic discharge − charge curves at 100 mA g^−1^ in dark and illuminated states. Reproduced with permission [100]. Copyright 2021, American Chemical Society. **d** Illustration of the preparation of MoS_2_-ONT. **e** Scheme of the photo-assisted Zn–air battery with MoS_2_-ONT cathode. Reproduced with permission [101]. Copyright 2023, Wiley-Blackwell

To address these limitations while enabling flexible implementation, Boruah et al. [100] developed innovative flexible photoelectrode architectures utilizing zinc oxide (ZnO) films deposited on carbon felt (CF) current collectors as electron-transport layers and hole-blocking layers, followed by MoS_2_ deposition to form mechanically adaptable photoelectrode cathodes (Fig. 6a). This flexible binary architecture leverages the complementary properties of both materials while accommodating mechanical deformation essential for flexible energy storage applications. MoS_2_’s optimal bandgap of 1.9 eV (Fig. 6b) exhibits exceptional matching with the solar spectrum, enabling efficient solar energy harvesting, while the flexible carbon felt substrate provides mechanical adaptability and electrical conductivity. The ZnO interlayer functions as both an electron-transport pathway and hole-blocking barrier, facilitating effective charge separation and preventing recombination losses that could compromise performance during mechanical deformation. Under illumination, this flexible architecture achieves remarkable photoelectric conversion efficiency of approximately 1.8% and solar energy conversion efficiency of 0.2%, with battery specific capacity increasing from 245 to 340 mAh g^−1^ (Fig. 6c). The system maintains 82% capacity retention after 200 cycles. The mechanistic advantage lies in the strategic band alignment between ZnO and MoS_2_, which creates favorable conditions for photogenerated electron extraction, while the flexible substrate architecture accommodates mechanical stress without compromising interfacial integrity.

A second innovative approach utilizes chemical vapor deposition (CVD) to create ordered one-dimensional MoS_2_ nanotube arrays (MoS_2_-ONT) within commercial anodic aluminum oxide (AAO) membranes (Fig. 6d) [101]. This confined flexible architecture represents a significant advancement in addressing both photoelectrochemical efficiency and mechanical adaptability challenges inherent to conventional MoS_2_ systems. The one-dimensional nanotube configuration provides enhanced mechanical flexibility compared to brittle 2D sheet structures while maintaining high surface area and catalytic activity essential for photoelectrochemical applications. The confined MoS_2_-ONT architecture effectively extends photogenerated carrier lifetime by creating internal field-like structures under photoexcitation, facilitating spatial charge separation. This confined geometry promotes efficient charge transport along the nanotube length while minimizing recombination losses that typically limit photoelectrochemical performance in conventional structures. The spatial confinement creates favorable conditions for maintaining photoelectrochemical activity. When integrated into photo-assisted zinc-air batteries (Fig. 6e), the MoS_2_-ONT-based system exhibits enhanced open-circuit voltage and successfully powers a 32-LED “JLU” circuit board in series configuration, demonstrating practical applicability for electronic devices. Under illumination, the photo-assisted zinc-air battery achieves impressive ORR kinetics of 70 mW cm^−2^, validating the effectiveness of the confined carrier separation strategy in energy storage applications.

This research demonstrates the universal applicability of confined carrier separation strategies in flexible photo-assisted metal-air batteries, opening new avenues for portable and wearable metal-air battery device integration. The success of both ZnO/MoS_2_ and MoS_2_-ONT approaches establishes fundamental design principles for flexible metal sulfide electrodes: (1) Strategic heterostructure formation that combines complementary materials for enhanced charge separation while maintaining mechanical flexibility; (2) Confined architectural design that promotes efficient charge transport and separation while accommodating mechanical stress; (3) Flexible substrate integration that provides mechanical support and electrical connectivity without compromising photoelectrochemical performance; and (4) Interface optimization that maintains charge transfer efficiency across mechanical deformation cycles.

1. Multielement Metal Sulfide Systems for Enhanced Flexible Performance

Compared with single metal sulfides, multi-metal sulfides exhibit higher redox activity and electron transfer efficiency due to the increased abundance of metal cations [102]. Metal cations provide more active sites and display variable chemical valences. At the same time, the conductivity of polysulfides is significantly higher than that of single metal sulfides, and compared with oxide composite materials, their conductivity is two orders of magnitude higher [103].

Based on this, Momeni et al. [104] grew titanium dioxide nanotubes (FTNs) on flexible titanium foil and loaded ternary metal sulfide Mn–Ni–Co–S (MNCS) with nanoflower structures via electrodeposition, forming self-supporting, binder-free composite electrodes (MNCS/FTNs). The TiO_2_ nanotubes provide a high specific surface area and ion diffusion channels, while the MNCS nanoflowers enhance visible light absorption and pseudocapacitive activity, synergistically improving light response and energy storage performance. The optimal photovoltaic electrode MNCST4 achieves an area-specific capacitance of 4,846 mF cm^−2^ at 1 mA cm^−2^, representing a 56% increase compared to dark conditions. The flexible symmetric supercapacitor assembled using MNCST4 (MNCST4@MNCS4/ITO) exhibits an areal capacitance of 941.6 mF cm^−2^ under illumination (617 mF cm^−2^ in dark conditions), with a capacitance retention rate of 90.6% after 10,000 cycles, and demonstrates excellent flexibility (stable performance at different bending angles). Additionally, the device can generate a photovoltage of 594 mV within 700 s under illumination without an external power source and successfully drive an LED to emit light, demonstrating its potential for application in flexible self-powered electronic devices.

For successful application of metal sulfides in flexible photo-assisted energy storage devices, it is essential to overcome mechanical fragility while ensuring that charge generation, separation, and transport efficiencies are maintained under mechanical deformation. This necessitates comprehensive optimization strategies encompassing: (1) Structural modification through nanostructuring approaches that enhance mechanical flexibility while preserving photoelectrochemical activity; (2) Interface engineering that maintains efficient charge transfer across material boundaries under mechanical stress; (3) Compositional tuning that optimizes both photoelectrochemical properties and mechanical characteristics; (4) Direct growth on flexible conductive substrates such as carbon cloth, carbon fiber paper, carbon fibers, and flexible metal foils that provide mechanical support and electrical connectivity; (5) Composite formation with flexible polymers that enhance mechanical durability while maintaining photoelectrochemical functionality; and (6) Device architecture design that accommodates strain through strategic structural configurations and material arrangements.

### New Two-Dimensional Materials: MXenes in Flexible Photo-Assisted Systems

Two-dimensional (2D) materials, characterized by their unique planar structures and inherent mechanical flexibility, have opened transformative avenues for advanced energy storage applications, particularly in flexible and wearable device configurations [105]. Among the diverse family of 2D materials, MXenes have emerged as exceptionally promising electrode materials for flexible photo-assisted energy storage devices due to their compelling combination of capacitive properties, metallic conductivity, optical transparency/absorption characteristics, tunable band structures, and excellent thermal conductivity [106–109]. Their distinctive 2D morphology enables superior interfacial contact with flexible substrates and demonstrates enhanced capability to accommodate mechanical strain compared to bulkier, more rigid conventional materials, making them ideally suited for flexible energy storage applications requiring both photoelectrochemical functionality and mechanical adaptability.

The intrinsic structural characteristics of MXenes [110], including their layered architecture, surface terminal groups, and metallic conductivity, provide multiple advantages for flexible photo-assisted energy storage: (1) Enhanced mechanical compliance through their 2D layered structure that can accommodate bending and stretching without significant performance degradation; (2) Efficient charge transport via metallic conductivity that maintains electrical connectivity under mechanical deformation; (3) Tunable photoelectrochemical properties through surface functionalization and intercalation chemistry; and (4) Superior volumetric energy storage due to high packing density and efficient ion intercalation mechanisms that remain functional across various mechanical states.

Ti_3_C_2_T_x_, where T_x_ denotes terminal functional groups (–O, –OH, and –F), represents one of the earliest reported and most extensively investigated MXene materials for flexible SC applications [111–114]. This material exhibits exceptional volumetric capacitance ranging from 1000 to 1500 F cm^−3^ in acidic electrolytes, attributed to its metallic conductivity and high chemical reactivity of surface titanium hydroxides/oxides [115, 116]. These properties, combined with the material’s inherent flexibility, make Ti_3_C_2_T_x_ particularly suitable for flexible photo-assisted energy storage applications where both high performance and mechanical adaptability are essential.

**Fig. 7:**
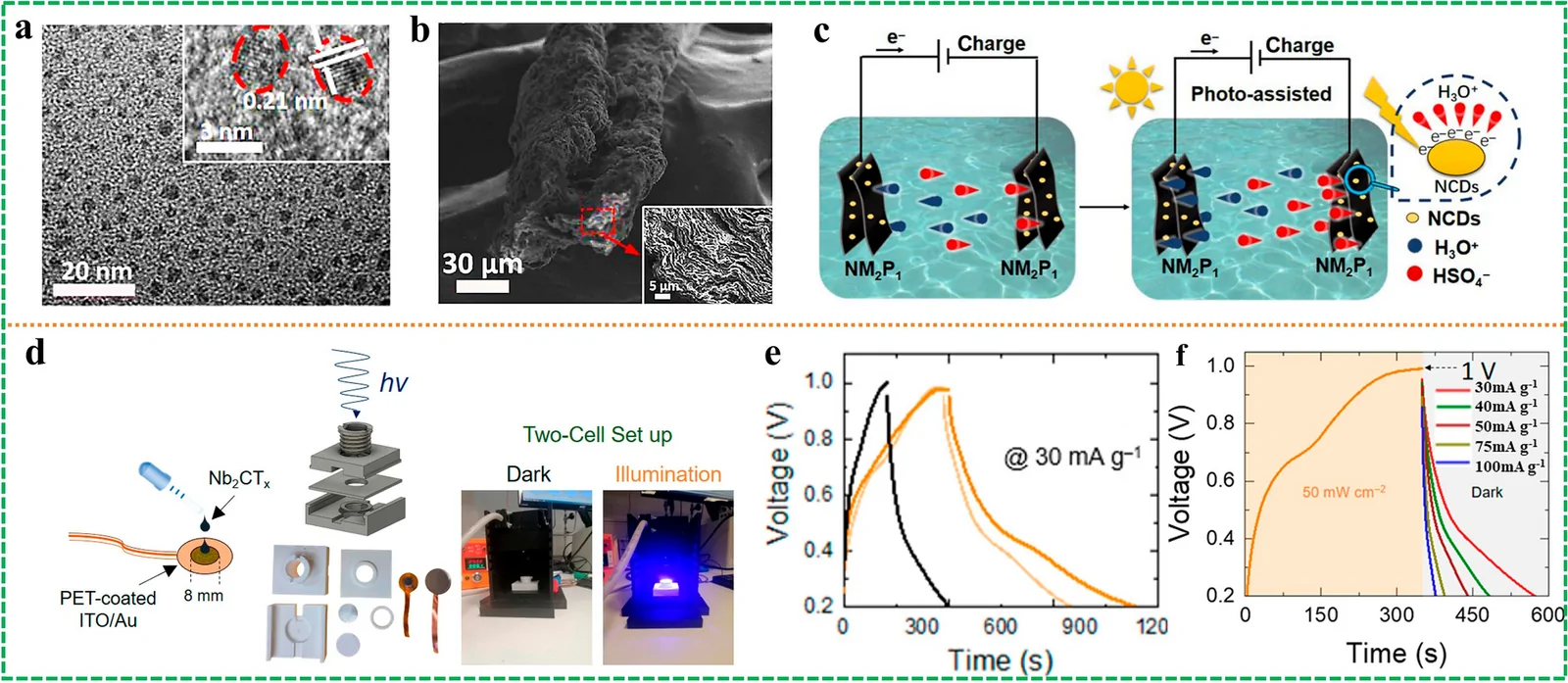
**a** Typical TEM and HRTEM (inset) images of NCDs. **b** SEM image of NM_2_P_1_ fiber and the corresponding section image (inset). **c** A schematic diagram of the working mechanism of the NM_2_P_1_ fiber as a photo-assisted charging SC. Reproduced with permission [117]. Copyright 2021, Tsinghua University Press. **d** Schematic illustration of the preparation of Nb_2_CT_x_-based photocathode and the two-electrode cell setup under dark and illumination conditions. **e** Comparative CD analyses of the Nb_2_CT_x_-based P-ZIC at 30 mA g^−1^ under dark, 25 mW cm^−2^ (light orange), and 50 mW cm^−2^ (dark orange) illumination (λ = 435 nm) conditions. **f** Photocharged (λ = 435 nm, 50 mW cm^−2^, at 0.02 mA cm^−2^) and dark-discharged (at different specific current rates) of the Nb_2_CT_x_-based P-ZIC. Reproduced with permission [118]. Copyright 2024, American Chemical Society

Building upon the established advantages of Ti_3_C_2_T_x_ for flexible applications, an innovative photo-assisted charging fiber SC (NM_2_P_1_) was developed using Ti_3_C_2_T_x_-based hybrid fibers strategically modified with nitrogen-doped carbon dots (NCDs) [117]. SEM analysis revealed the distinctive morphological characteristics of NCDs (Fig. 7a) and cross-sectional features (inset), while the NM_2_P_1_ fiber exhibited a rough, corrugated surface structure (Fig. 7b). The corrugated surface morphology provides increased surface area for electrochemical reactions. Under photo-assisted charging conditions, the flexible NM_2_P_1_ fibers achieved remarkable volumetric capacitance of 1445 F cm^−3^ at 10 A cm^−3^, representing a substantial 35.9% enhancement compared to dark charging conditions (1063 F cm^−3^), with maximum volumetric energy and power densities reaching 18.75 mWh cm^−3^ and 8382 mW cm^−3^, respectively. The sophisticated operational mechanism involves spontaneous intercalation of electrolyte cations (H_3_O^+^) into Ti_3_C_2_T_x_ interlayers during charging, facilitating electrochemical redox reactions at TiO or TiO_2_ surfaces (Fig. 7c). While illumination does not directly affect H_3_O^+^ intercalation dynamics, photoexcited NCDs generate abundant charge carriers, with photogenerated electrons transferring to Ti_3_C_2_T_x_ surfaces, electrostatically enhancing H_3_O^+^ intercalation efficiency. During discharge, H_3_O^+^ deintercalates from the electrode surface, combining with HSO_4_^−^ in the electrolyte to complete the electrochemical cycle. The photoelectrochemical enhancement mechanism operates through surface charge modification rather than structural changes, ensuring that the beneficial effects persist across repeated mechanical deformation cycles essential for flexible device operation.

Nb_2_CT_x_, a distinctive member of the MXene family, has attracted significant attention for flexible photo-assisted energy storage applications due to its exceptional electrical conductivity and high carrier mobility characteristics, enabling efficient charge–discharge dynamics [119]. These intrinsic properties facilitate rapid charge storage and release mechanisms, while simultaneously supporting catalytic functionalities either as a direct catalyst or catalyst support in photoelectrochemical processes, including HER and OER. In HER processes, the synergistic interaction between terminal groups on the Nb_2_CT_x_ surface and metal atoms effectively reduces reaction activation energy, enhancing reaction kinetics while maintaining catalytic activity. This catalytic functionality, combined with photoelectrochemical activity, provides multiple pathways for energy conversion and storage in flexible device configurations. The integration of Nb_2_CT_x_ into photoenhanced hybrid zinc-ion capacitors (PZIC) as a bifunctional photoactive electrode material demonstrates remarkable capability for both photoenhanced capacitive performance and efficient charge storage (Fig. 7d) [118]. Under experimental conditions with light intensity of 50 mW cm^−2^, the photo-driven capacitance of the Nb_2_CT_x_-based photo-zinc-ion capacitor (PZIC) increased by over 60% compared to operation at 25 mW cm^−2^ light intensity (approximately 18 F g^−1^). This resulted in photoenhanced specific capacitance of approximately 27 F g^−1^ (Fig. 7e), while maintaining output voltage of 1.0 V (Fig. 7f). The system demonstrated excellent cycling stability, retaining approximately 85% capacitance after 3000 cycles, establishing new benchmarks for photoenhanced energy storage systems.

The successful implementation of MXenes in fiber SCs directly demonstrates their applicability in flexible device. However, widespread implementation in flexible and wearable systems faces critical challenges that must be systematically addressed to realize their full potential. The primary challenges include: (1) High production costs that limit scalable manufacturing for commercial flexible devices; (2) Chemical stability concerns, especially under dynamic mechanical and environmental conditions typical of flexible applications; (3) Interface degradation during repeated mechanical deformation that can compromise long-term performance; and (4) Processing compatibility with flexible substrate materials and device fabrication techniques.

To address these constraints and unlock MXenes’ full potential for flexible applications, comprehensive optimization strategies are essential: (1) Cost-effective synthesis development through scalable, high-yield production methodologies that maintain material quality while reducing manufacturing costs; (2) Stability enhancement through surface engineering approaches, protective encapsulation strategies, and compositional modifications suitable for flexible device environments; (3) Interface optimization that maintains efficient charge transfer and mechanical integrity across deformation cycles; and (4) Flexible device architecture innovation that leverages MXenes’ unique properties while providing protection from environmental and mechanical degradation.

### Organic Photosensitive Materials for Flexible Photo-Assisted Energy Storage

Organic photosensitive materials have emerged as crucial components in flexible photo-assisted energy storage devices, offering distinctive advantages that complement inorganic photoactive materials in mechanically adaptable energy storage systems [120]. These materials serve dual strategic roles: as active electrode materials that bring inherent flexibility and processability to flexible device architectures, and as performance-enhancing additives in batteries and SCs that improve photoelectrochemical functionality while maintaining mechanical compliance [121]. The fundamental advantages of organic photosensitive materials for flexible applications include: (1) Intrinsic mechanical flexibility arising from their molecular structure and intermolecular interactions that can accommodate deformation without catastrophic failure; (2) Solution processability enabling low-temperature fabrication on flexible substrates through coating, printing, and other scalable manufacturing techniques; (3) Tunable optoelectronic properties through molecular engineering and chemical modification; and (4) Efficient photogenerated carrier generation under illumination that promotes redox reactions at electrode surfaces, ultimately enhancing photo-assisted charging efficiency and energy storage capacity while remaining compatible with deformable device architectures.

However, the implementation of organic photosensitive materials in flexible photo-assisted energy storage systems faces critical challenges that must be systematically addressed. The operational stability of certain organic photosensitive materials remains a primary concern, especially under the combined mechanical and photoelectrochemical stress experienced in flexible devices during repeated deformation cycles [122]. This necessitates comprehensive optimization strategies encompassing molecular engineering approaches, including introduction of stabilizing functional groups and structural modifications that improve not only chemical stability and intrinsic photoelectric properties but also mechanical robustness essential for constructing resilient flexible energy storage systems.

#### Carbon Dots in Flexible Photo-Assisted Systems

Carbon dots (CDs), innovative carbon nanoparticles smaller than 10 nm, represent a particularly promising class of organic photosensitive materials for flexible photo-assisted energy storage applications. CDs are characterized by abundant surface functional groups including hydroxyl (–OH), carbonyl (C=O), and carboxyl (–COOH) moieties that provide multiple advantages for flexible device integration [123]. Their exceptional photoactive properties significantly enhance photogenerated electron–hole pair separation efficiency and demonstrate superior charge transport characteristics, while their small size and surface functionality enable excellent dispersion in flexible matrices and compatibility with various flexible substrate materials [124].

**Fig. 8:**
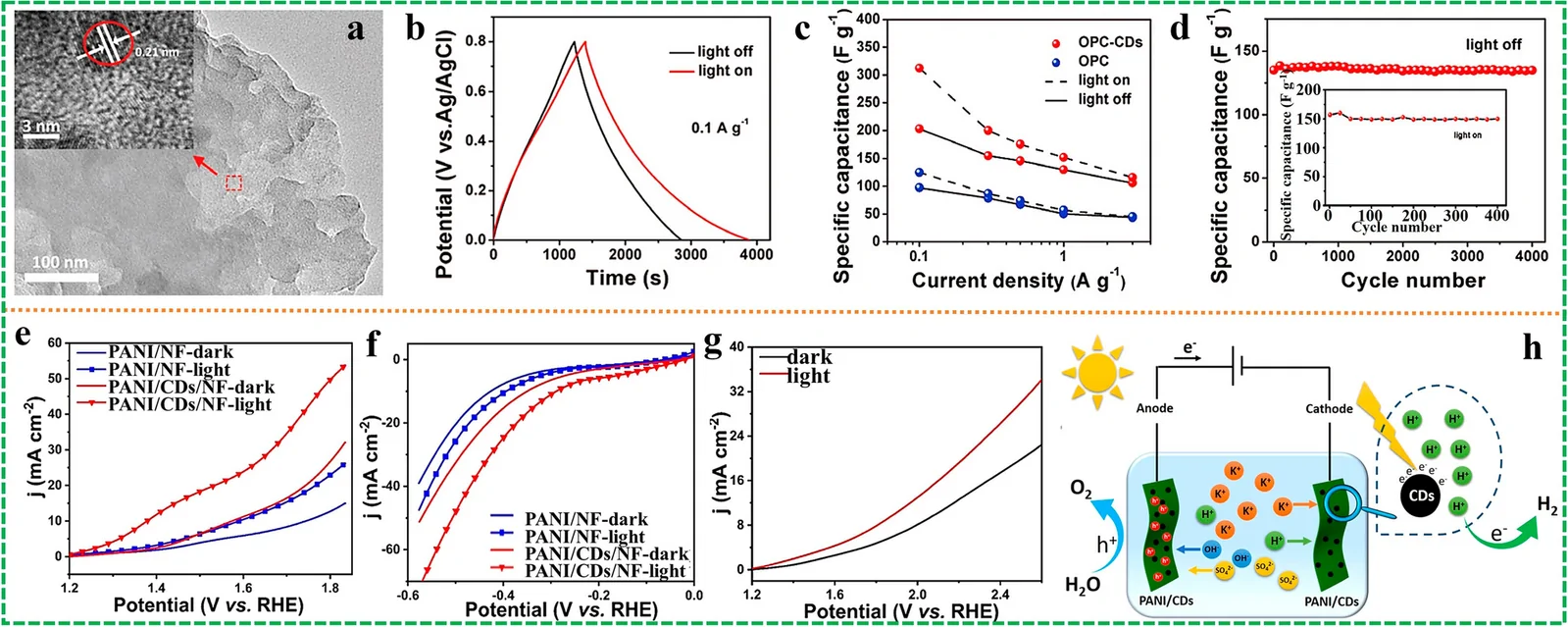
**a** TEM and HRTEM (inset) images of OPC-CDs-700 show that CD still distributes on OPC after high-temperature treatment. **b** GCD curves of OPC-CDs-700 at 0.1 A g^−1^ with/without light illumination. **c** Specific capacitance at different current densities of OPC-CDs-700 with/without light illumination indicates that OPC-CDS-700 exhibits higher photo-enhanced capacitance under illumination. **d** The cycling stability of the OPC-CDs-700 electrode by charge–discharge measurement at 1 A g^−1^ in dark condition and light illumination (inset) indicates that the OPC-CD-700 electrode exhibits good stability. Reproduced with permission [126]. Copyright 2020, Royal Society of Chemistry. **e** Comparison of OER LSV curves shows that CD is beneficial for accelerating reaction kinetics and improving the light enhancement capability of PANI/CD in OER. **f** Comparison of HER LSV curves shows that CD is beneficial for improving the light enhancement capability of PANI/CD in HER. **g** Comparison of LSV curves of PANI/ CDs/NF for full water splitting (scan rate, 5 mV s^−1^). **h** Mechanism diagram of P-EC overall water splitting shows that the addition of CD enhances the photoelectric effect of the catalyst. Reproduced with permission [127]. Copyright 2021, American Chemical Society

Recent advancements have successfully utilized CD-based nanocomposites as electrode materials in flexible SCs, enabling more efficient charge transport pathways and enhancing electrolyte–electrode interfacial interactions during charge–discharge processes under mechanical deformation. Consequently, these materials exhibit superior specific capacitance and rate performance that are maintained across various mechanical states, making them particularly suitable for flexible energy storage applications requiring both high performance and mechanical adaptability [125]. To explore the previously uninvestigated effects of CDs on electrical double-layer capacitor (EDLC) behavior under illumination in flexible configurations, Huang et al. [126] developed an innovative photo-assisted rechargeable SC (OPC-CDs-700) by integrating proanthocyanidins with CDs, creating a electrode material suitable for mechanically adaptable energy storage applications (Fig. 8a). Under illumination, the OPC-CDs-700 system not only demonstrates substantial photocurrent generation but also exhibits strategic changes in impedance characteristics. This performance enhancement can be attributed to CDs functioning as photoactive media that stabilize charges and enhance surface charge accumulation, thereby improving energy storage efficiency. The mechanistic advantage of CDs in applications involves multiple synergistic effects: Photogenerated electrons and holes are efficiently separated and transported through the CD network, while surface functional groups facilitate enhanced electrolyte interaction and charge storage. Furthermore, controlled partial CD decomposition during operation increases the OPC-CDs-700’s specific surface area, facilitating enhanced charge transfer. The OPC-CDs-700 system exhibits impressive specific capacitance of 312 F g^−1^ under illumination, corresponding to a substantial 54.4% enhancement relative to performance under dark conditions (Fig. 8b). Additionally, the system maintains high specific capacitance of 106 F g^−1^ at current density of 3 A g^−1^ (Fig. 8c), illustrating outstanding rate capability. The OPC-CDs-700 system exhibits exceptional cycling stability over 4000 cycles (Fig. 8d), maintaining electrochemical performance throughout extended operation. This research advances solar energy utilization technology while providing novel design strategies for photosensitive energy devices.

#### Conducting Polymers in Flexible Photo-Assisted Systems

Conducting polymers represent another crucial class of organic photosensitive materials for flexible photo-assisted energy storage, offering unique combinations of electrical conductivity, photoelectrochemical activity, and mechanical flexibility [128]. Polyaniline (PANI), a p-type semiconductor material, demonstrates exceptional photoelectric conversion characteristics under radiation conditions, generating significant photocurrent while maintaining these properties under mechanical deformation, making it an ideal candidate for flexible photo-assisted electrocatalytic devices [129, 130]. Additionally, PANI’s ability to provide protons during reaction processes facilitates both HER and OER while accommodating the mechanical stress typical of flexible device operation. The inherent flexibility of PANI chains, combined with their processability through solution-based techniques, enables integration into flexible device architectures without compromising photoelectrochemical functionality. This compatibility with flexible substrates and device configurations makes PANI particularly suitable for wearable and conformable energy storage applications [131].

Building upon PANI’s advantageous properties for flexible applications, Liu et al. [127] developed a metal-free bifunctional photo-assisted catalyst composed of PANI/CDs, capitalizing on PANI's advantages including low cost, straightforward preparation processes, abundant raw material availability, and excellent compatibility with flexible device fabrication. The integration of CDs with PANI creates a synergistic flexible composite that combines the photoelectrochemical advantages of both materials. Under neutral electrolyte conditions and illumination, the flexible PANI/CDs/NF electrode exhibits significant performance enhancements. Specifically, the overpotential is reduced by 150 mV for OER and by 65 mV for HER, achieving current densities of 30 and 20 mA cm^−2^, respectively (Fig. 8e, f). At 2.0 V, the illuminated current density reaches 13.27 mA cm^−2^, representing a substantial 62.8% enhancement compared to dark conditions (8.15 mA cm^−2^) (Fig. 8g). The enhanced performance mechanism in PANI/CDs systems (Fig. 8h) involves sophisticated photoelectrochemical processes. At the cathode, photogenerated electrons at the carbon dot surface combine with protons derived from the electrolyte and supplied by PANI, facilitating H_2_ generation through enhanced HER kinetics. Simultaneously, at the anode, illumination induces generation of additional photogenerated holes on the PANI surface, which actively participate in OER processes with improved efficiency. The integration of CDs significantly enhances the photoelectric effect of PANI/CDs systems through multiple mechanisms: (1) Enhanced light absorption through complementary optical properties of PANI and CDs; (2) Improved electron-transport efficiency via CD-mediated charge transport pathways; (3) Reduced electron–hole pair recombination rates through spatial charge separation; and (4) Accelerated HER and OER kinetics through synergistic catalytic effects. These mechanisms collectively establish the PANI/CDs system as an effective metal-free bifunctional photo-assisted electrocatalyst for flexible energy applications. This research establishes novel strategies for enhancing metal-free bifunctional photo-assisted electrocatalyst performance through solar energy utilization while maintaining compatibility with flexible device requirements. The successful demonstration of PANI/CDs systems provides valuable insights for developing next-generation flexible photo-assisted energy storage devices that combine high performance with mechanical adaptability.

The successful implementation of organic photosensitive materials in flexible photo-assisted energy storage devices requires systematic addressing of several critical challenges. The operational stability of organic photosensitive materials under combined mechanical and photoelectrochemical stress represents a primary concern that must be resolved for practical flexible device applications. Repeated mechanical deformation can induce structural changes, interfacial degradation, and performance loss that compromise long-term device functionality. Molecular engineering approaches are vital for overcoming these limitations and realizing the full potential of organic photosensitive materials in flexible applications. Strategic approaches include: (1) Stabilizing functional group introduction that enhances chemical stability and resistance to degradation under mechanical stress; (2) Structural modifications that improve both photoelectric properties and mechanical robustness; (3) Composite design optimization that combines organic photosensitive materials with flexible matrices to enhance overall system performance; (4) Interface engineering that maintains efficient charge transfer across deformation cycles; and (5) Processing technique development that enables scalable manufacture of stable, high-performance flexible devices.

### Organic Framework Materials for Flexible Photo-Assisted Energy Storage

Organic framework materials, including metal–organic frameworks (MOFs), covalent organic frameworks (COFs), and porous organic polymers, represent a highly promising class of materials for flexible photo-assisted energy storage devices [132–134]. These crystalline porous materials offer unique advantages through their tunable molecular architectures, customizable optical and electronic properties, and inherent potential for flexible film formation. By strategically designing organic framework structures with complementary photoactive and electroactive components, these materials can achieve enhanced light harvesting, efficient charge separation and transport, and improved mechanical flexibility suitable for deformable energy storage platforms [135]. The modular nature of organic frameworks allows for precise control over pore size, surface chemistry, and electronic band structures, making them ideal candidates for developing next-generation flexible photo-assisted batteries and SCs that can adapt to various mechanical configurations while maintaining superior electrochemical performance.

#### Metal–Organic Frameworks

**Fig. 9:**
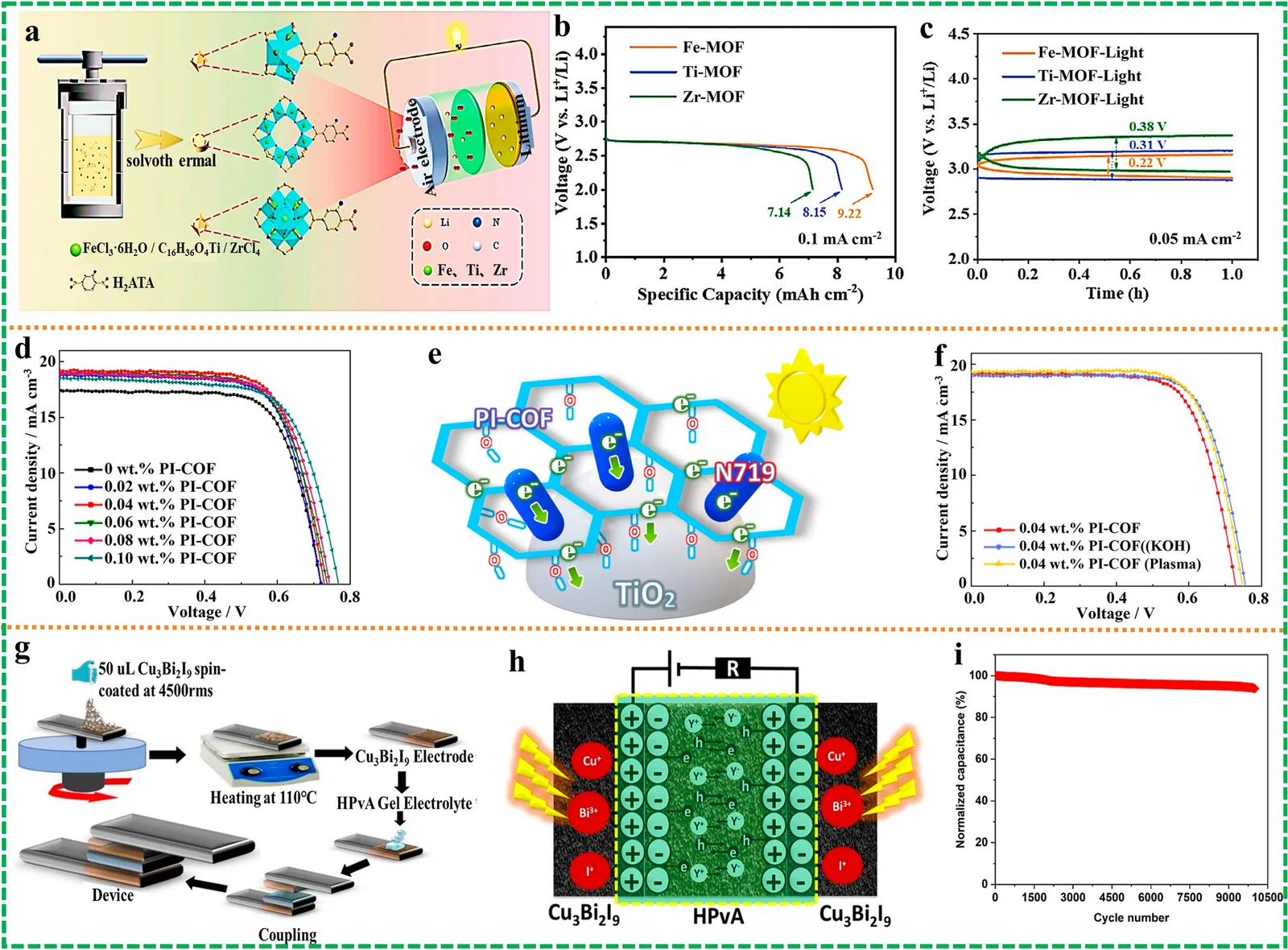
**a** Synthesis process and structural diagram of Fe/Ti/Zr-MOF. **b** Constant current discharge curves of Fe/Ti/Zr-MOF cathodes. **c** Galvanostatic charge–discharge curves of Fe/Ti/Zr-MOF cathodes with illumination. Reproduced with permission [138]. Copyright 2024, Wiley–VCH Verlag. **d** J–V curves and for the solar devices employing the TiO_2_ photoelectrodes with various doping amounts of PI-COF. **e** Schematic of the host (PI-COF)-guest (N719) interaction and the bridging between PI-COF and TiO_2_ via hydroxyl interaction. **f** J–V curves for the solar devices employing the TiO_2_ photoelectrodes with the doping of PI-COF modified by KOH and plasma. Reproduced with permission [142]. Copyright 2022, American Chemical Society. **g** Schematic for fabrication of Cu-perovskite photo-assisted SC. **h** Photo-electrochemical energy storage mechanism of Cu-perovskite photo-assisted SC. **i** Normalized capacitance versus cycle number of 10,000 for Cu-perovskite photo-assisted SC. Reproduced with permission [143]. Copyright 2023, Elsevier B.V

MOFs have emerged as promising candidates for catalyzing lithium-oxygen battery (LOB) reactions due to their tunable structures and abundant active sites [136]. The distinctive architecture of MOFs, comprising metal centers and organic linkers, enables strong oxygen adsorption at metal sites, effectively reducing reaction barriers. Furthermore, certain MOFs exhibit semiconductor characteristics through visible light absorption, generating photocarriers that participate in ORR and OER, demonstrating exceptional performance across various photocatalytic applications [137]. Their high surface area and hierarchical porous structure provide abundant exposed active sites and transport channels, enhancing their potential as photoelectrodes in photo-assisted LOBs. Through a facile solvothermal method, MOFs with different metal clusters (Fe, Ti, Zr) were successfully synthesized and implemented as efficient photocathodes in photo-assisted LOBs (Fig. 9a) [138]. Notably, Fe-MOF-doped LOBs demonstrated superior electrochemical performance through a unique dual excitation pathway, where both -NH_2_ groups and Fe–O clusters undergo direct photoexcitation under illumination. The unsaturated metal sites serving as catalytic active centers, combined with this distinctive excitation mechanism, enable Fe-MOF to more effectively promote ORR/OER processes during discharge/charge cycles, thereby enhancing the kinetics of LOB reactions. Compared to conventional inorganic semiconductor crystals, Fe-MOF exhibits superior specific surface area and oxygen adsorption capabilities. Consequently, Fe-MOF-based LOBs achieved remarkable areal capacity (9.22 mAh cm^−2^), ultra-low charge–discharge overpotential (0.22 V) under illumination (Fig. 9b, c), and exceptional cycling stability exceeding 195 cycles, significantly outperforming Ti-MOF, Zr-MOF, and TiO_2_ cathodes. This research underscores the potential of MOFs in photo-assisted LOBs and provides crucial insights for their future development in photo-assisted metal-air batteries.

#### Covalent Organic Frameworks

COFs have garnered significant attention in energy and photoelectronic applications due to their exceptional characteristics, including high porosity, superior thermal stability, and highly ordered conjugated structures that facilitate charge migration, separation, and light harvesting. These materials demonstrate extensive potential across photoelectric devices, gas/energy storage, and heterogeneous catalysis [139–141]. Polyimide (PI)-based COFs, synthesized through imide ring formation, exhibit remarkable thermal and chemical stability alongside superior mechanical strength. The high porosity and large surface area of PI-COF materials promote efficient light collection and charge injection. Through the condensation reaction between pyromellitic dianhydride (PMDA) and tris(4-aminophenyl)amine (TAPA), polyimide COFs (PI-COFs) were synthesized and utilized to modify TiO_2_ photoelectrodes, establishing a novel strategy for photovoltaic enhancement in dye-sensitized solar cells (DSSCs) [142]. Notably, DSSCs incorporating 0.04 wt.% PI-COF-doped TiO_2_ photoelectrodes achieved optimal solar efficiency of 9.93%, accompanied by an enhanced short-circuit current density (J_SC_) of 19.03 mA cm^−2^ (Fig. 9d). The improved J_SC_ performance is attributed to PI-COFs’ dual functionality, enhancing charge transfer/injection through host (PI-COF)-guest (N719 dye) interactions while suppressing charge recombination (Fig. 9e). Additionally, PI-COFs serve as co-sensitizers, facilitating the generation of photogenerated electrons under solar illumination. Surface modification of PI-COFs via oxygen plasma treatment further enhanced their hydrophilicity and connectivity with TiO_2_ nanoparticles, thereby achieving an improved solar efficiency of 10.46% and a J_SC_ of 19.43 mA cm^−2^, as shown in Fig. 9f. This approach of modifying photoelectrodes through PI-COF incorporation in TiO_2_ nanoparticles presents significant potential in DSSC development.

#### Perovskite

Metal halide perovskite materials with ABX_3_ structure have attracted significant attention due to their exceptional electronic and ionic conduction properties, where A sites are occupied by MA^+^, Cs^+^, or Ag^+^, B sites by Pb^2+^ or Sn^2+^, and X sites by I^−^, Br^−^, or Cl^−^. These materials demonstrate remarkable advantages in photoelectric conversion and photo-ionic applications due to their broad-spectrum light absorption capabilities spanning ultraviolet, visible, and near-infrared regions [144, 145]. Through the innovative integration of novel composite materials with traditional perovskite structures, researchers [143] successfully developed a copper-based photo-assisted SC utilizing Cu_3_Bi_2_I_9_ inorganic perovskite materials (Fig. 9g), thereby achieving both efficient energy harvesting and electrochemical storage capabilities. Under illumination, the photoactive copper-based perovskite electrode generates photogenerated carriers (electron–hole pairs) that undergo effective separation and migration under applied voltage, reducing recombination losses and promoting charge accumulation at the electrode surface (Fig. 9h). Experimental results indicate a significant 127% increase in energy density at a scan rate of 0.01 V s^−1^, along with outstanding cycling stability, retaining 93.8% of its capacity after 10,000 charge–discharge cycles (Fig. 9i). These findings highlight the significant potential of such devices in future wearable and portable flexible electronic applications.

In summary, perovskite materials represent a transformative class of photoactive components for flexible photo-assisted energy storage systems, offering unique advantages through their tunable crystal structures, exceptional optoelectronic properties, and solution-processable nature that facilitates flexible device fabrication. The ability to modify perovskite compositions and structures provides unprecedented opportunities for optimizing both light absorption characteristics and mechanical flexibility, making them particularly suitable for next-generation wearable electronics and conformable energy storage devices. However, the practical implementation of perovskite-based flexible energy storage systems requires addressing key challenges including moisture sensitivity, thermal stability, and mechanical robustness under repeated deformation. Future research directions should focus on developing stable perovskite formulations, protective encapsulation strategies, and flexible substrate integration techniques to fully realize the potential of these materials in commercial flexible photo-assisted energy storage applications.

## Design Strategies and Principles for Performance Enhancement in Flexible Photo-Assisted Systems

The development of high-performance flexible photo-assisted energy storage systems requires systematic implementation of advanced materials design strategies that simultaneously optimize photoelectrochemical efficiency and mechanical resilience. These systems must address unique challenges inherent to flexible applications, including maintaining electrical conductivity under mechanical deformation, preserving interfacial integrity during repeated flexing cycles, and ensuring long-term stability in mechanically demanding environments. The fundamental design principle involves creating integrated material architectures that synergistically combine optimized band structures for enhanced light absorption, superior electrical conductivity for reliable charge transport, enhanced photoelectric conversion efficiency, tunable bandgap architectures for broad spectral response, exceptional photochemical stability, and high theoretical storage capacity—all while maintaining mechanical robustness under bending, stretching, or twisting conditions. To achieve these multifaceted requirements, five core optimization strategies have been systematically developed and validated for flexible photo-assisted energy storage applications.

The performance enhancement of flexible photo-assisted energy storage systems relies on the systematic implementation of five interconnected design strategies: (1) Doping modification strategies that incorporate strategic elemental additions to enhance photoelectric properties while maintaining mechanical compatibility with flexible substrates, enabling band structure engineering and spectral response optimization without compromising structural integrity; (2) Surface modification and interface engineering approaches that utilize controlled surface treatments to optimize electrode–electrolyte interfaces, which prove particularly suitable for flexible configurations as they minimally impact bulk mechanical properties while significantly enhancing charge transfer efficiency; (3) Heterostructure engineering that strategically combines materials with complementary electronic and optical properties to improve carrier separation efficiency, with critical focus on maintaining interfacial integrity under mechanical stress through compatible material selection and optimized interface design; (4) Morphology control and nanostructure engineering that develops hierarchical architectures maximizing light absorption efficiency while inherently providing enhanced strain tolerance through stress-distributing geometries, making them ideally suited for flexible electrode applications; and (5) Composite material strategies that integrate photoactive materials with mechanically flexible and electrically conductive components (including carbon nanotubes, graphene, and flexible current collectors) to enhance both light-harvesting capabilities and overall device durability under mechanical deformation.

The practical implementation of these design strategies requires careful consideration of substrate and current collector selection, which serve as the mechanical foundation for flexible photo-assisted energy storage systems. State-of-the-art flexible current collectors, including polyethylene terephthalate (PET) substrates, three-dimensional graphene foams, and carbon cloths, provide the essential mechanical framework while maintaining electrical connectivity [146–148]. Carbon cloth substrates demonstrate particular effectiveness due to their cost-effectiveness, chemical inertness, and interconnected three-dimensional porous architectures that facilitate efficient electron transport and electrolyte infiltration while enabling robust mechanical compliance under various deformation modes [149]. The successful integration of cerium dioxide/manganese dioxide nanoparticles on carbon cloth substrates exemplifies effective materials integration, achieving energy densities of 0.024 mWh cm^−3^ at power densities of 0.306 mW cm^−3^ with 83% capacity retention after 10,000 cycles and stable performance under 180° bending conditions [31]. This systematic approach to materials integration demonstrates how strategic substrate selection enables the practical implementation of advanced design strategies while maintaining the mechanical flexibility required for wearable and portable applications. The comprehensive framework presented provides a robust foundation for developing next-generation flexible photo-assisted energy storage systems that achieve enhanced performance metrics through rational materials design while ensuring practical applicability in mechanically demanding operational environments.

### Doping Modification in Flexible Electrodes

Doping modification constitutes a fundamental design strategy for optimizing photoelectrochemical performance in flexible energy storage systems through precise control of electronic and optical properties. This approach involves the strategic introduction of foreign elements to engineer band structures, expand spectral response ranges, and enhance charge transport characteristics while maintaining the mechanical flexibility required for deformable applications. The core design principle leverages atomic-level modifications to address key performance limitations in photo-assisted systems: narrow light absorption windows, rapid charge recombination, and insufficient electrical conductivity. For flexible applications, doping strategies must simultaneously optimize photoelectrochemical efficiency and preserve mechanical integrity, ensuring that enhanced performance persists under repeated deformation cycles. This dual optimization requires careful selection of dopant elements and concentrations that improve both optical and electrical properties without compromising the host material’s structural flexibility.

Doping modification enhances flexible photo-assisted system performance through several interconnected mechanisms that directly address fundamental limitations in energy storage applications. Band structure engineering through strategic elemental incorporation induces spectral red-shifts and broadening, enabling electrode materials to capture visible and near-infrared photons that would otherwise remain unutilized. This spectral expansion proves particularly valuable for flexible devices operating under diverse illumination conditions, where maximizing light-harvesting efficiency across broad wavelength ranges becomes critical. The Ni-doped BiOBr nanosheets exemplify effective doping strategy implementation, demonstrating significantly expanded light absorption capabilities and enhanced photogenerated carrier separation efficiency. Under UV illumination, these modified photoelectrodes achieve maximum specific capacitances of 362.73 F g^−1^—representing a 1.45-fold improvement compared to dark conditions. Meanwhile, the energy density of Ni–BiOBr//reduced graphene oxide asymmetric supercapacitors can reach 39.95 Wh kg^−1^, and after 1,000 cycles under light conditions, they still maintain a capacitance retention rate of 68%, demonstrating excellent cycle stability. This also verifies the effectiveness of strategic doping in enhancing performance [150]. Simultaneously, conductivity enhancement through doping addresses charge transport limitations that become exacerbated in flexible configurations where mechanical stress can disrupt conductive pathways. Nitrogen-doped graphene films demonstrate this principle, providing inherent flexibility while maintaining high conductivity that suppresses carrier recombination and reduces charging overpotentials in lithium-oxygen batteries [151].

Effective doping strategies for flexible photo-assisted energy storage systems require systematic consideration of multiple design parameters that influence both performance and mechanical reliability. Primary design guidelines include: dopant selection based on electronic compatibility with host materials to ensure stable incorporation without inducing structural defects, concentration optimization to maximize performance benefits while preserving mechanical properties, and processing compatibility with flexible substrate manufacturing techniques. The spatial distribution of dopants critically influences performance, with uniform dispersion promoting consistent photoelectrochemical behavior while gradient doping can create built-in electric fields that enhance charge separation. For practical implementation, doping strategies should prioritize thermodynamically stable dopant incorporation to prevent segregation during operation, electrochemical compatibility to avoid unwanted side reactions during cycling, and scalable synthesis methods suitable for large-area flexible device production. This systematic approach to doping modification provides a robust framework for developing high-performance flexible photo-assisted energy storage systems that achieve enhanced photoelectrochemical efficiency while maintaining the mechanical flexibility required for practical applications.

### Surface Modification and Interface Engineering for Flexible Devices

Surface modification and interface engineering represent critical design strategies for optimizing charge transport and photoelectrochemical performance in flexible photo-assisted energy storage systems. These approaches focus on creating functional interfaces that facilitate efficient photogenerated carrier separation, transport, and utilization while maintaining structural integrity under mechanical deformation. The fundamental design principle involves strategically modifying electrode surfaces to enhance light–matter interactions, suppress charge recombination, and accelerate interfacial charge transfer processes. For flexible applications, surface modifications must simultaneously improve photoelectrochemical efficiency and preserve mechanical connectivity, ensuring that enhanced performance persists during repeated bending cycles. This dual optimization requires careful selection of surface modification materials and architectures that provide both electronic enhancement and mechanical resilience.

Quantum dot integration on electrode surfaces exemplifies sophisticated surface modification strategies that leverage size-dependent quantum effects to enhance photoresponsive performance across broad spectral ranges. The systematic incorporation of black phosphorus quantum dots (BPQDs) onto Co_2_V_2_O_7_/CNT macroporous membranes demonstrates effective multi-interface engineering where photogenerated carrier separation creates abundant active sites for electronic structure modulation, significantly improving conductivity and charge accumulation capabilities. Under illumination, photoexcited electrons participate directly in energy storage processes while BPQDs stabilize charges to facilitate substantial surface charge accumulation, achieving exceptional specific capacities of 138.4 mAh g^−1^ (197.9 mAh cm^−3^) under illuminated conditions. At the same time, the CNT@Co_2_V_2_O_7_/BPQD supercapacitor exhibits a maximum energy density of 44.4 Wh kg^−1^ (60.0 Wh L^−1^) at a power density of 800 W kg^−1^ (960 W L^−1^) and maintains excellent cycle stability of 104.8% after 13,000 charge–discharge cycles [152]. This performance enhancement illustrates the synergistic effects achievable through strategic surface modification design. Similarly, conductive nanomaterial integration, particularly carbon nanotube (CNT) and graphene modifications, provides dual functionality by enhancing both carrier transport efficiency and mechanical resilience of flexible electrode layers. CNT-modified flexible electrodes enable rapid electron migration through efficient transport channels that maintain connectivity during mechanical deformation, reducing recombination losses while preserving electrochemical performance [153].

Effective surface modification strategies for flexible photo-assisted energy storage systems require systematic consideration of multiple design parameters that influence both photoelectrochemical performance and mechanical reliability. Key design principles include: interface compatibility to ensure stable adhesion between modification layers and substrate materials during mechanical stress, electronic coupling optimization to facilitate efficient charge transfer across modified interfaces, and processing scalability for practical flexible device manufacturing. The spatial arrangement and coverage of surface modifications critically influence performance, with uniform distribution promoting consistent photoelectrochemical behavior while selective modification can create gradient interfaces that enhance charge separation. For practical implementation, surface modification strategies should prioritize materials with comparable mechanical properties to prevent stress concentration during flexing, electrochemical stability to avoid interfacial degradation during cycling, and synthesis methods compatible with roll-to-roll processing techniques. This systematic approach to surface modification and interface engineering provides a robust framework for developing flexible photo-assisted energy storage systems that achieve enhanced charge transport efficiency while maintaining mechanical flexibility required for practical deformable applications.

### Heterostructure Engineering

Heterostructure engineering represents a fundamental design strategy for enhancing performance in flexible photo-assisted energy storage systems by creating interfaces between materials with complementary electronic and optical properties. The formation of heterojunctions generates built-in electric fields that facilitate efficient charge separation and suppress carrier recombination—critical factors for achieving high photoelectrochemical performance in deformable architectures [154, 155]. For flexible applications, the core design principle involves strategic material selection to create synergistic interfaces that maintain interfacial integrity under mechanical stress while preserving photoelectrochemical functionality. Key design considerations include band alignment optimization for efficient charge transfer, mechanical compatibility between components for flexible device integration, and interface engineering to minimize defect-induced recombination centers.

**Fig. 10:**
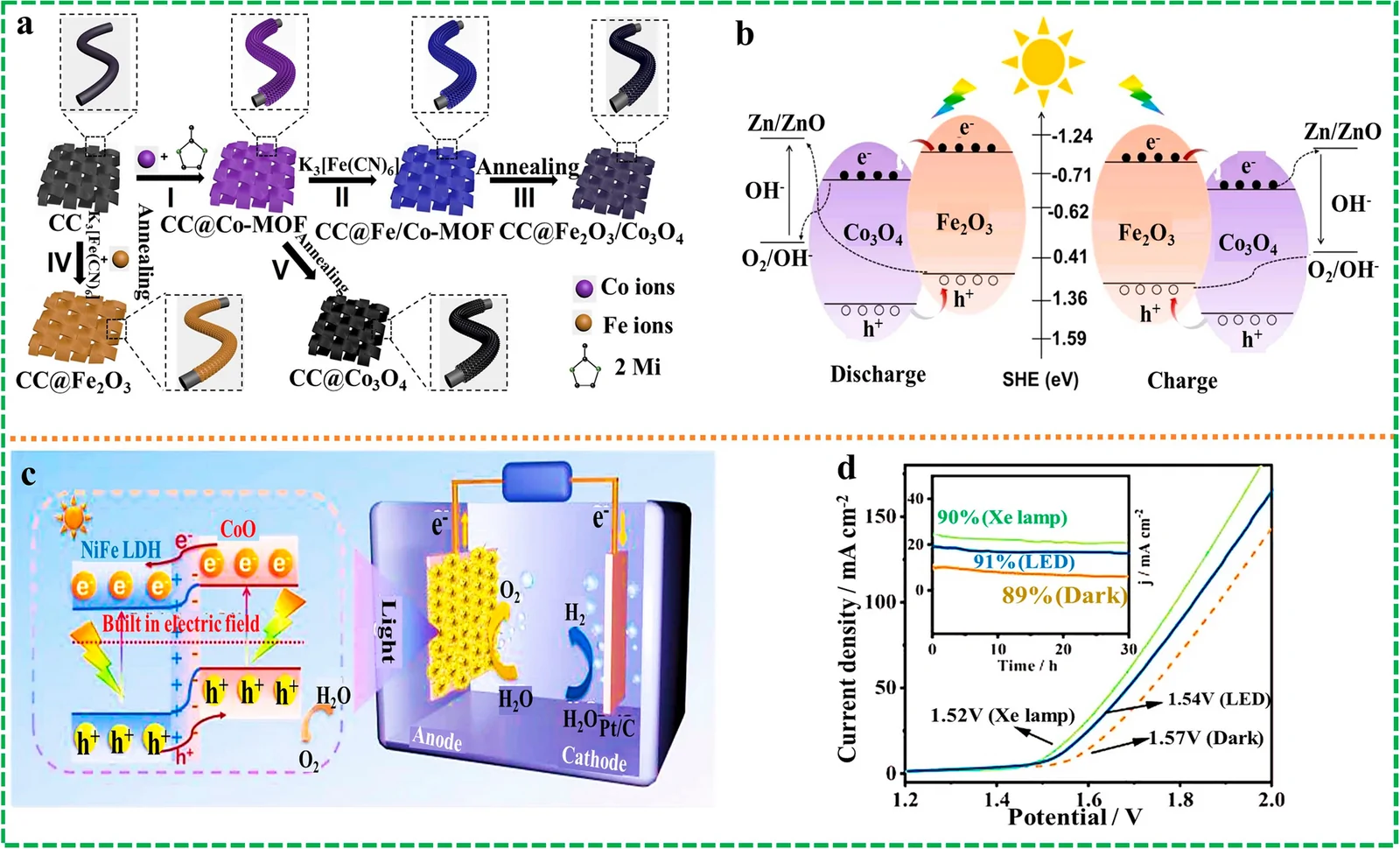
**a** Schematic formation process of the Fe_2_O_3_/Co_3_O_4_(I–III), Fe_2_O_3_ (IV), and Co_3_O_4_ (I and V), respectively. **b** Schematic mechanism of the charge and discharge process under light irradiation. Reproduced with permission [156]. Copyright 2024, Elsevier. **c** Schematic diagram of photo-assisted electrocatalytic water splitting. **d** LSV curves of overall water splitting performance by using CoO/NiFe LDH as the anode and 20% Pt/C as the cathode. (Inset: The overall water splitting stability test with and without irradiation. Reproduced with permission [157]. Copyright 2024, Elsevier Ltd

Strategic implementation of heterostructure design has demonstrated remarkable success in various flexible photo-assisted systems [158]. The Fe_2_O_3_/Co_3_O_4_ heterojunction exemplifies effective design for flexible zinc-air batteries, where oxygen vacancy-rich interfaces facilitate charge separation, while carbon cloth substrates provide mechanical flexibility (Fig. 10a). Under illumination, engineered band alignment enables photogenerated holes to transfer from Co_3_O_4_ to Fe_2_O_3,_ while electrons move oppositely (Fig. 10b), creating spatial charge separation that improves energy efficiency from 64% to 78% [156]. Similarly, the CoO/NiFe LDH system demonstrates complementary material selection where CoO provides enhanced light absorption, while NiFe LDH offers abundant electrocatalytic active sites (Fig. 10c). The strong electronic coupling creates synergistic effects that reduce oxygen evolution overpotentials by 60 mV under illumination (Fig. 10d), showcasing how heterostructure design addresses multiple performance limitations simultaneously [157].

The performance enhancement mechanisms in heterostructured flexible photo-assisted systems operate through enhanced light harvesting via complementary absorption ranges, improved charge separation through built-in electric fields, reduced recombination losses via spatial carrier separation, and accelerated reaction kinetics through optimized active site distribution. For practical implementation in flexible devices, heterostructure design should prioritize interfacial stability under mechanical deformation, scalable synthesis methods compatible with flexible substrate processing, material compatibility to prevent degradation during cycling, and optimized thickness to balance performance with mechanical flexibility. This systematic engineering approach provides a robust framework for developing high-performance flexible photo-assisted energy storage systems through rational interface design and strategic material integration.

### Morphology Control and Nanostructure Engineering for Optimized Photoelectrochemical Performance

Morphology control and nanostructure engineering represent fundamental design strategies for achieving optimized photoelectrochemical performance in flexible photo-assisted energy storage systems through precise control of structural architectures at multiple length scales. The rational design of electrode morphologies must simultaneously address three critical requirements: maximizing photoelectrochemical efficiency through enhanced light–matter interactions, ensuring mechanical flexibility through stress-tolerant structural designs, and maintaining electrochemical performance through optimized mass transport pathways [159, 160]. Unlike conventional rigid systems where structural optimization focuses primarily on electrochemical performance, flexible photo-assisted devices require nanostructures that preserve both photoelectrochemical functionality and structural integrity under repeated mechanical deformation [161]. The fundamental design principle involves creating hierarchical architectures that synergistically combine high surface area for enhanced light absorption, optimized pore structures for efficient electrolyte infiltration and ion transport, and mechanical robustness through stress-distributing geometries that prevent structural failure during flexing cycles.

The morphological design requirements for flexible photo-assisted energy storage systems vary systematically based on the underlying energy storage mechanism and operational performance targets. Battery electrodes often adopt particle-based or thin-film morphologies, prioritizing compact packing for high volumetric energy density while ensuring sufficient electronic conductivity. In contrast, SC electrodes favor highly porous or fibrous architectures, designed to maximize specific surface area and minimize ion diffusion pathways, which are critical for achieving high power density. This morphological divergence directly aligns with their distinct energy storage mechanisms. For SC applications, the optimal design strategy prioritizes highly porous or fibrous architectures that maximize electrochemically active surface area while minimizing ion diffusion pathways—critical factors for achieving high power density in mechanically deformable configurations. Nanoflower and nanosheet morphologies demonstrate exceptional effectiveness in this context, as exemplified by ZnCo_2_O_4_ nanoflower structures where needle-like “petals” facilitate one-dimensional photoelectric charge transfer while enabling radial light propagation through multiple absorption and scattering events within the hierarchical structure. This sophisticated morphological design achieves significantly enhanced specific capacitance (563 F g^−1^) under illumination compared to dark conditions. Meanwhile, hollow spherical structures of CuCo_2_S_4_ (CCS HS) were prepared as the anode. Its specific capacity under illumination was 305 F g^−1^, approximately twice that under non-illuminated conditions. More importantly, the energy density of the assembled photosensitive ZCO NF//CCS HS ASC reaches 60.9 Wh kg^−1^ under illumination, whereas it is only 46.5 Wh kg^−1^ under non-illumination conditions. Therefore, this study provides quantitative validation for the principles of structural performance optimization [162]. In contrast, battery electrode design emphasizes achieving optimal balance between volumetric energy density and photocatalytic efficiency through strategic nanostructure engineering. Nanoparticle and nanowire array configurations demonstrate superior light energy absorption and enhanced photogenerated carrier generation while simultaneously reducing lithium-ion diffusion pathways through shortened transport distances. The strategic integration of customized crystal facets, particularly (002) orientations in nanorod array/microsphere composite structures, creates synergistic morphological effects where optimized spacing reduces local current density, while high surface area microspheres provide adequate buffer space for lithium deposition, ultimately reducing electrochemical overpotentials by 8.3 mV during photo-assisted charging processes [163].

The performance enhancement achieved through morphology-controlled flexible photo-assisted systems operates through multiple interconnected mechanisms that collectively optimize photoelectrochemical efficiency: enhanced light harvesting through increased surface area and optimized light-trapping geometries that maximize photon absorption across broad spectral ranges, improved charge separation through reduced carrier diffusion distances that minimize recombination losses, accelerated ion transport via shortened diffusion pathways that enhance power density, and maintained mechanical stability through stress-distributing hierarchical structures that preserve functionality during deformation. For practical implementation in flexible device architectures, morphological design strategies should systematically prioritize several critical considerations: scalable synthesis methods compatible with roll-to-roll processing and flexible substrate manufacturing requirements, structural resilience under repetitive mechanical stress to prevent performance degradation during operational cycling, optimized thickness-to-flexibility ratios that achieve optimal balance between energy density and mechanical bendability, and interface compatibility between nanostructured active materials and flexible current collectors to ensure robust electrical connectivity. Additionally, the morphological design must consider thermal stability during processing and operation, electrochemical compatibility to prevent structural degradation during cycling, and cost-effective manufacturing scalability for practical commercialization. This comprehensive approach to morphology control and nanostructure engineering provides a systematic framework for developing high-performance flexible photo-assisted energy storage systems that maintain optimal functionality under mechanical deformation while maximizing photoelectrochemical efficiency through rational structural design.

### Composite Material Strategies for Enhanced Spectral Response and Mechanical Robustness

Composite material strategies represent a sophisticated design approach for developing high-performance flexible photo-assisted energy storage systems that address the inherent limitations of individual components while achieving synergistic performance enhancement. The fundamental design principle involves strategically combining materials with complementary properties—optical, electrical, and mechanical—to create integrated systems that exceed the capabilities of their constituent components. For flexible photo-assisted applications, composite strategies must simultaneously optimize light-harvesting efficiency across broad spectral ranges, maintain electrochemical performance under mechanical deformation, and ensure long-term stability during repeated flexing cycles. This approach enables the development of robust flexible devices that can effectively utilize diverse light sources while preserving functionality under real-world operating conditions.

The strategic integration of materials with complementary light absorption characteristics represents a core composite design strategy for expanding photosensitive response ranges in flexible energy storage systems. Individual electrode materials typically exhibit limited spectral absorption windows that constrain their photoelectrochemical efficiency under practical illumination conditions. The TiO_2_/CdS composite system exemplifies effective spectral complementarity design, where TiO_2_’s ultraviolet absorption properties combine with CdS’s visible light capabilities to create broad-spectrum photoresponse, yielding a potential gain of over 2 V during charging [164]. This synergistic integration enables electrode materials to capture photons across extended wavelength ranges, significantly enhancing solar utilization efficiency and increasing photogenerated carrier density for energy storage processes. Beyond binary composites, multi-component systems can be engineered to achieve near-complete solar spectrum coverage by incorporating narrow-bandgap semiconductors for near-infrared absorption, intermediate-bandgap materials for visible light harvesting, and wide bandgap components for ultraviolet capture. The design challenge lies in optimizing component ratios and spatial arrangements to minimize charge recombination at interfaces while maximizing collective light absorption efficiency.

Effective composite design for flexible photo-assisted energy storage systems requires careful consideration of multiple interdependent factors that influence both performance and mechanical reliability. Key design principles include: component compatibility to ensure stable interfaces under mechanical stress, morphological optimization to maintain electrical connectivity during flexing, processing compatibility for scalable manufacturing on flexible substrates, and thermal stability to prevent degradation during operation. The spatial arrangement of composite components critically influences performance, with core–shell architectures providing enhanced charge separation, while layered configurations enable sequential light absorption and charge transfer. For practical implementation, composite strategies should prioritize materials with similar thermal expansion coefficients to minimize stress concentration during temperature cycling, electrochemically stable interfaces to prevent degradation during repeated charge–discharge cycles, and processable synthesis routes compatible with roll-to-roll manufacturing techniques. This systematic approach to composite design provides a robust framework for developing flexible photo-assisted energy storage systems that achieve enhanced performance through strategic material integration while maintaining mechanical integrity under operational conditions.

In the field of light-assisted flexible energy storage, the choice of design strategy affects light absorption capacity, conductivity, structure, and electrochemical performance. Therefore, selecting an appropriate design strategy is crucial for achieving high-performance light-assisted flexible energy storage materials. Table 5 summarizes the advantages and disadvantages of different design strategies and their application ranges in light-assisted flexible energy storage materials. The five strategies each have their own focus in photonic-assisted flexible energy storage devices: Doping modification and morphology control are more focused on optimizing the intrinsic properties of the material, while heterostructures and surface modification are focused on charge dynamics regulation. The composite material strategy is the core means of balancing photonic performance and mechanical flexibility. In practical applications, performance breakthroughs are often achieved through the synergistic use of multiple strategies.

**Table 5:** The advantages and disadvantages of different design strategies and their scope of application in light-assisted flexible energy storage materials

Strategy type	Advantages	Disadvantages	Core application scenarios
Doping Modification	Precise adjustment of bandgap and conductivity to improve photogenerated carrier separation efficiency Mature process that is easily compatible with flexible substrates Low cost, suitable for large-scale production	Doping concentration must be strictly controlled; excessive doping may lead to lattice defects Limited improvement in mechanical flexibility Doping with precious metals may increase costs	Regulate the electronic structure of electrode materials to optimize light absorption and conductivity
Surface Modification and Interface Engineering	Reduce interfacial charge transfer resistance and improve rate performance Enhance the compatibility between electrodes and gel electrolytes to reduce leakage Introduce light-responsive sites to indirectly improve photovoltaic efficiency	Modified layers tend to peel off during bending, affecting stability Complex modification processes increase costs Excessive modification may block electrode pores	Optimize electrode surface characteristics and electrode–electrolyte interface to reduce charge transfer resistance
Heterostructure Engineering	Heterojunction built-in electric field efficiently separates charge carriers, improving photovoltaic performance Integrates the advantages of different materials Synergistically improves performance stability under bending conditions	Interface lattice mismatch can easily lead to an increase in defects Multi-material composite processes are complex, and uniformity is difficult to guarantee Long-term cycling may result in interface diffusion	Constructing semiconductor heterojunctions to promote photogenerated charge separation
Morphology Control and Nanostructure Engineering	Nanostructures increase specific surface area and increase active sites Ordered structures promote ion/electron transport and reduce resistance Adapt to the bending deformation of flexible substrates and improve mechanical stability	Complex nanostructure preparation processes are difficult to scale up Excessively high aspect ratios may increase brittleness Nanoparticles tend to agglomerate, affecting performance consistency	Regulate the microscopic morphology of electrodes to optimize light absorption and ion transport
Composite Material Strategies	Synergistic enhancement of light absorption and mechanical strength Flexible substrate enables device adaptation to bending/folding Easy performance balancing through component ratio adjustment	Poor compatibility between multiple components increases charge transfer resistance Uniformity of composite materials is difficult to guarantee Some flexible substrates are costly, and their conductivity decreases after bending	Composite light-responsive materials and flexible substrates, balancing spectral response and mechanical robustness

## Photo-Assisted Flexible Energy Storage Devices

This section presents a comprehensive examination of photo-assisted flexible energy storage devices, focusing on the fundamental integration principles that combine photoelectrochemical energy conversion with mechanical deformability to create next-generation portable energy systems. The analysis systematically addresses the critical technological challenges arising from the intersection of photoelectrochemical functionality and mechanical flexibility, providing detailed insights into how light-harvesting capabilities can be effectively integrated with the conformability and resilience requirements essential for wearable and portable applications. The discussion encompasses the historical evolution of this emerging field, recent technological breakthroughs, and systematic evaluation of device architectures specifically designed for flexible photo-assisted energy storage applications.

The core focus centers on understanding how strategic materials design and device engineering approaches enable the development of flexible energy storage systems that maintain photoelectrochemical efficiency under mechanical deformation. This analysis evaluates the advantages and inherent limitations of different device configurations when adapted for flexible formats, with particular emphasis on the strategic methodologies employed to overcome fundamental challenges including: maintaining electrical connectivity during deformation, preserving photoelectrochemical interfaces under mechanical stress, optimizing light utilization in curved or twisted configurations, and ensuring long-term stability in mechanically demanding environments. The investigation systematically examines accelerated reaction kinetics and enhanced electrochemical performance achieved through photo-assistance in flexible battery and SC configurations, providing quantitative assessment of performance improvements compared to conventional dark operation.

A systematic comparative analysis, presented in Table 6, categorizes representative photo-assisted flexible energy storage devices based on photoelectrode material classifications, device architectures, and quantified performance metrics including energy density, power density, cycling stability, and mechanical flexibility parameters. This comprehensive evaluation framework enables systematic assessment of technological progress and identification of key performance bottlenecks limiting practical implementation. The analysis aims to establish fundamental understanding of photo-assisted flexible energy storage technology development trajectories while providing strategic insights for future research directions in portable electronics, smart textiles, biomedical implants, and emerging wearable technologies. Through this systematic approach, the section establishes a robust foundation for advancing photo-assisted flexible energy storage systems toward practical commercial applications that leverage both photoelectrochemical enhancement and mechanical adaptability for next-generation portable energy solutions.

**Table 6:** Structures and performances of typical light-assisted energy storage devices

Type	Photo electrode	Light source	Current density	Capacitance or capacity	Energy density	SC/PE/RTE	Mechanical flexibility	Cycle	References
SC	Se–V_2_O_5_–PPy		0.5 A g^−1^	108 F g^−1^	59.47 Wh Kg^−1^		Bend to any angle	91% (10,000 cycles)	[165]
	CuxO/np-NiCu@NiCuO/MG, *x* = 1, 2	Xe lamp	1.0 A cm^−3^	1182.2 F cm^−3^	44.9 mWh cm^−3^	118% (PE)	~ 100% after 1000 bends	93% (2000 cycles)	[166]
	Co_3_O_4_ nanospheres	100 mW cm^−2^	1 A g^−1^	523 F g^−1^	97.6 Wh kg^−1^	116.7% (PE)		42.5% (5000 cycles)	[94]
	MnO_2_–V_2_O_5_/WTNTS	1 Sun	6.0 A g^−1^	237.6 F g^−1^				94% (5000 cycles)	[95]
	CoMnS/TNT	100 W m^−2^ Xe light	0.70 mA cm^−2^	71.7 mF cm^−2^		170% (PE)		94% (5000 cycles)	[167]
	CeO_2_–MnO_2_		1.3 mA cm^−2^	110.57 mF cm^−2^	0.024 mWh cm^−2^		Bend to 180°	95% (4000 cycles)	[31]
	NCDs@Ti_3_C_2_T_x_	150 mW cm^−2^	10 A cm^−3^	1445 F cm^−3^	18.75 mWh cm^−3^	135.9% (PE)			[117]
Li–O_2_	Ov–TiO_2_	1 Sun	100 mA g^−1^	9390 mAh g^−1^					[78]
	TiO_2_–Fe_2_O_3_	500 W Xe lamp	0.05 mA cm^−2^	0.1 mAh cm^−2^		86% (RTE)		86% (100 cycles)	[168]
	CVO@CNT	Xe lamp	0.1 mA cm^−2^	6.14 mAh cm^−2^		166% (PE)			[169]
	FeNi-TCPP	100 mW cm^−2^	0.1 mA cm^−2^			92% (RTE)			[170]
Li–CO_2_	CoPc–Mn–O@rGO	1 Sun	0.02 mA cm^−2^			98.5% (RTE)			[171]
Li–S	PHK	500W Xe lamp	5 C	679 mAh g^−1^				67% (1500 cycles)	[172]
	3DHG/NS/CPANI		0.5 C	1082 mAh g^−1^				80% (500 cycles)	[173]
	CdS–TiO_2_/CC	0.5 Sun	0.2 mA cm^−2^	1225 mAh g^−1^		2.3%(SC)			[174]
	Ti-BPDC	30 mW cm^−2^	0.2 C	1314 mAh g^−1^		4.3%(SC)		82.9% (150 cycles)	[175]
Li–N_2_	Au–N_v_–C_3_N_4_	500 W Xe lamp	100 mA g^−1^	600 mAh g^−1^		59.1% (RTE)			[176]
Zn-Air	MoS_2_-ONT	30 W LED UV	0.5 mA cm^−2^	799.5 mAhg^−1^					[101]
	pTTh	100 mW cm^−2^	0.1 mA cm^−2^	553.3 mAh g^−1^	681.71 Wh kg^−1^	156.25% (PE)	Restore the original state after deformation		[177]
	CC@Fe_2_O_3_/Co_3_O_4_	300W Xe lamp	0.1 mA cm^−2^	712.3 mAh g^−1^		14.4% (PE)			[156]
Zn-Ion	V_2_O_5_	100 mW cm^−2^	0.1 A g^−1^	473 mAh g^−1^		5.2%(SC)		89.3% (4000 cycles)	[178]
	V_2_O_3_@CSs	100 mW∙cm^−2^	2.0 A g^−1^	463 mAh g^−1^		0.354% (SC)		61% (3000 cycles)	[179]
Sn-Air	Fe_2_O_3_@TiO_2_/Ti	300 mW cm^−2^	0.10 mA cm^−2^						[180]

RTE represents the round-trip efficiency; PE represents the photo-conversion efficiency; SC represents solar conversion

### Photo-Assisted Flexible SCs

Photo-assisted flexible SCs represent a paradigmatic convergence of photoelectrochemical energy conversion and mechanical deformability, addressing critical limitations in conventional energy storage systems while enabling novel applications in wearable electronics and distributed energy systems. These devices leverage the inherent advantages of SCs—including high power density, extended cycle life, and rapid charge–discharge capabilities—while integrating photoelectrochemical enhancement mechanisms to address energy density limitations and mechanical flexibility requirements for portable applications [3, 12, 126, 181–185]. The fundamental design challenge involves creating electrode architectures that simultaneously maintain efficient charge storage under mechanical deformation, preserve photoelectrochemical interfaces during flexing cycles, and optimize light utilization across various geometric configurations. This integration requires systematic consideration of materials selection, interfacial engineering, and device architecture optimization to achieve synergistic performance enhancement between photoelectrochemical functionality and mechanical adaptability.

The conceptual foundation for photo-assisted SCs, initially established through “photocapacitor” designs in 2004, introduced novel photoelectrode configurations comprising dye-sensitized semiconductor nanoparticles integrated with charge storage components [11]. While initial designs prioritized photoelectrochemical functionality over mechanical flexibility, the principles established the groundwork for subsequent integration strategies addressing flexible applications. Early photoelectrode architectures, featuring dye-sensitized semiconductor nanoparticles, hole-capturing layers, and activated carbon, enabled direct storage of photogenerated charges, laying the foundation for advanced flexible implementations. Subsequent developments in perovskite-type solar capacitors, despite their ~ 1 μm thickness offering potential for conformability, revealed the necessity for careful material selection and device engineering to achieve true flexibility, particularly in addressing mechanical fragility of photoactive layers and ensuring stable interfaces under strain [186].

**Fig. 11:**
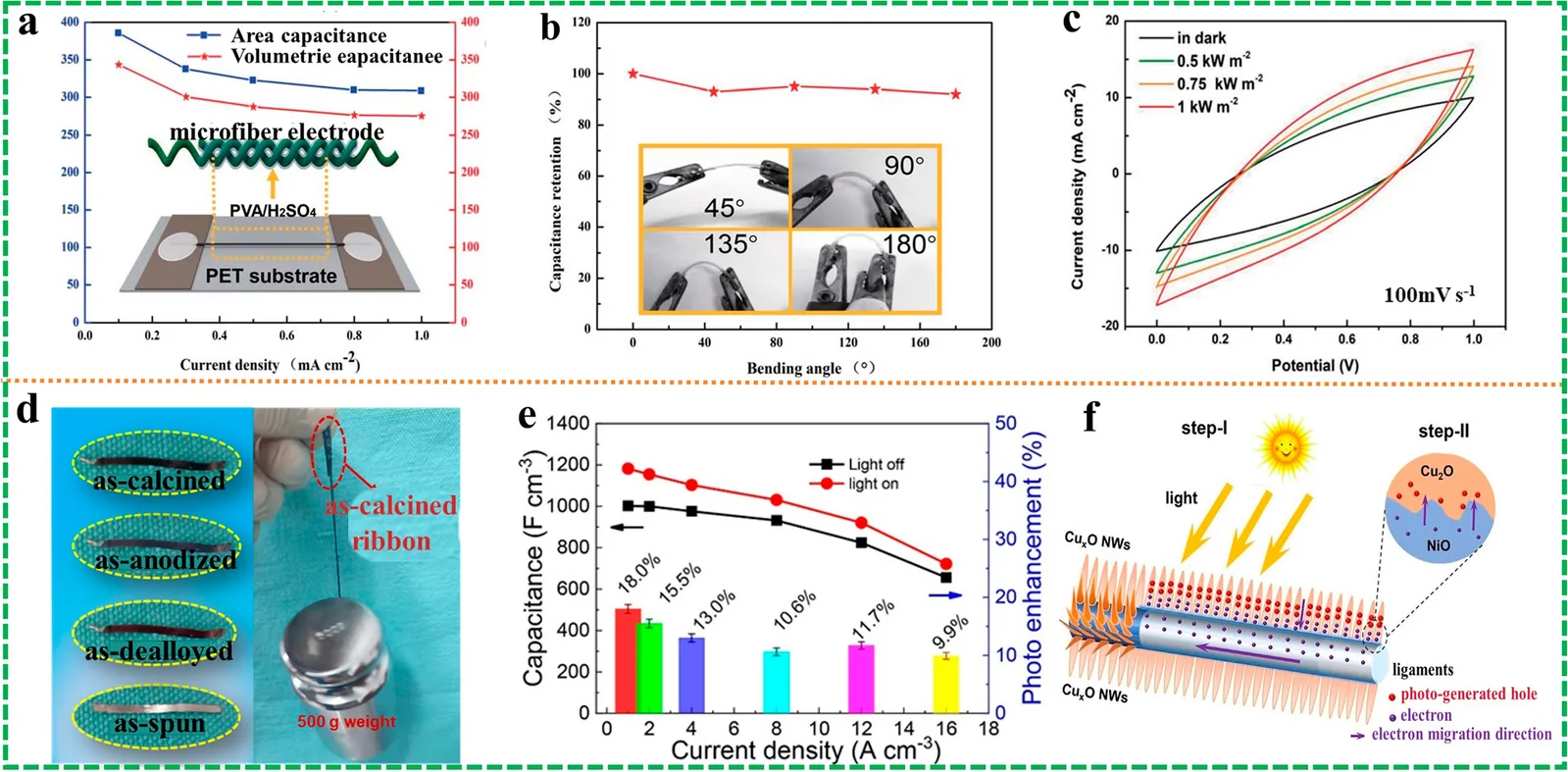
**a** Volume capacitance and area capacitance of a single CSMFE of the GE/CNFs@PANI ASSMFSC at different current densities. Schematic of the GE/CNFs@PANI ASSMFSC structure (inset). **b** Capacitance retentions of the GE/CNFs@PANI ASSMFSC with different bending angles at the current density of 0.5 mA cm^−2^ (the inset: bending test experiment diagram). **c** CV curves of the GE/ CNFs@PANI ASSMFSC under different solar power densities, respectively. Reproduced with permission [187]. Copyright 2020, Royal Society of Chemistry. **d** Digital photographs of the as-dealloyed, as-anodized, and as-calcined ribbons. **e** Specific capacitance as a function of current density. **f** Schematic illustration of enhanced charge storage mechanism by light irradiation for the Cu_x_O/np-NiCu@NiCuO/MG (x = 1, 2) hybrid photoelectrode. Reproduced with permission [166]. Copyright 2023, Elsevier

The evolution toward truly flexible architectures has necessitated addressing fundamental challenges including mechanical fragility of photoactive layers, interface stability under strain, and temperature-dependent performance variations critical for wearable applications where ambient temperature can vary significantly. Advanced multi-functional device development exemplifies successful integration strategies, such as all-solid-state microfiber SCs utilizing cellulose nanofiber-graphene conjugated polymers, demonstrating inherent suitability for flexible and even weavable electronics (Fig. 11a) [187]. These core-sheath microfiber electrode configurations achieve substantial volumetric capacitance and energy density while, critically, maintaining stable performance under severe bending deformation, with photothermal conversion-enhanced capacitance validating the feasibility of flexible photothermal energy conversion systems (Fig. 11b, c). This research establishes CSMFE-based microfiber SCs as promising candidates for flexible photothermal energy conversion systems, creating new paradigms for next-generation flexible energy-related devices.

Transition metal oxides, including TiO_2_, Cu_2_O, Bi_2_O_3_, ZnO, Co_3_O_4_, demonstrate exceptional potential for flexible photo-assisted SC applications through their combined pseudocapacitive characteristics and photosensitivity when processed appropriately as thin films or nanostructures on flexible supports [12, 188–194]. Strategic materials integration, exemplified by Cu_x_O/np-NiCu@NiCuO/MG hybrid photoelectrodes synthesized from Ni_35_Cu_15_Zr_15_Ti_35_ metallic glass precursors, achieves remarkable structural versatility including post-calcination flexibility capable of folding while supporting 500g loads, hierarchical porosity, and heterostructure configurations (Fig. 11d) [166]. Under illumination, these flexible photoelectrodes demonstrate maximum specific capacitances of 1182.2 F cm^−3^—representing 18% enhancement over dark conditions—through synergistic photogenerated carrier enhancement and heterostructure effects (Fig. 11e, f). Crucially, these photoelectrodes demonstrate excellent photocurrent response and flexibility, maintaining stable photocurrent even after 1000 bending cycles, with aqueous SC devices assembled using these flexible electrodes achieving good energy densities and retaining 93% capacitance after 2000 cycles under illumination.

**Fig. 12:**
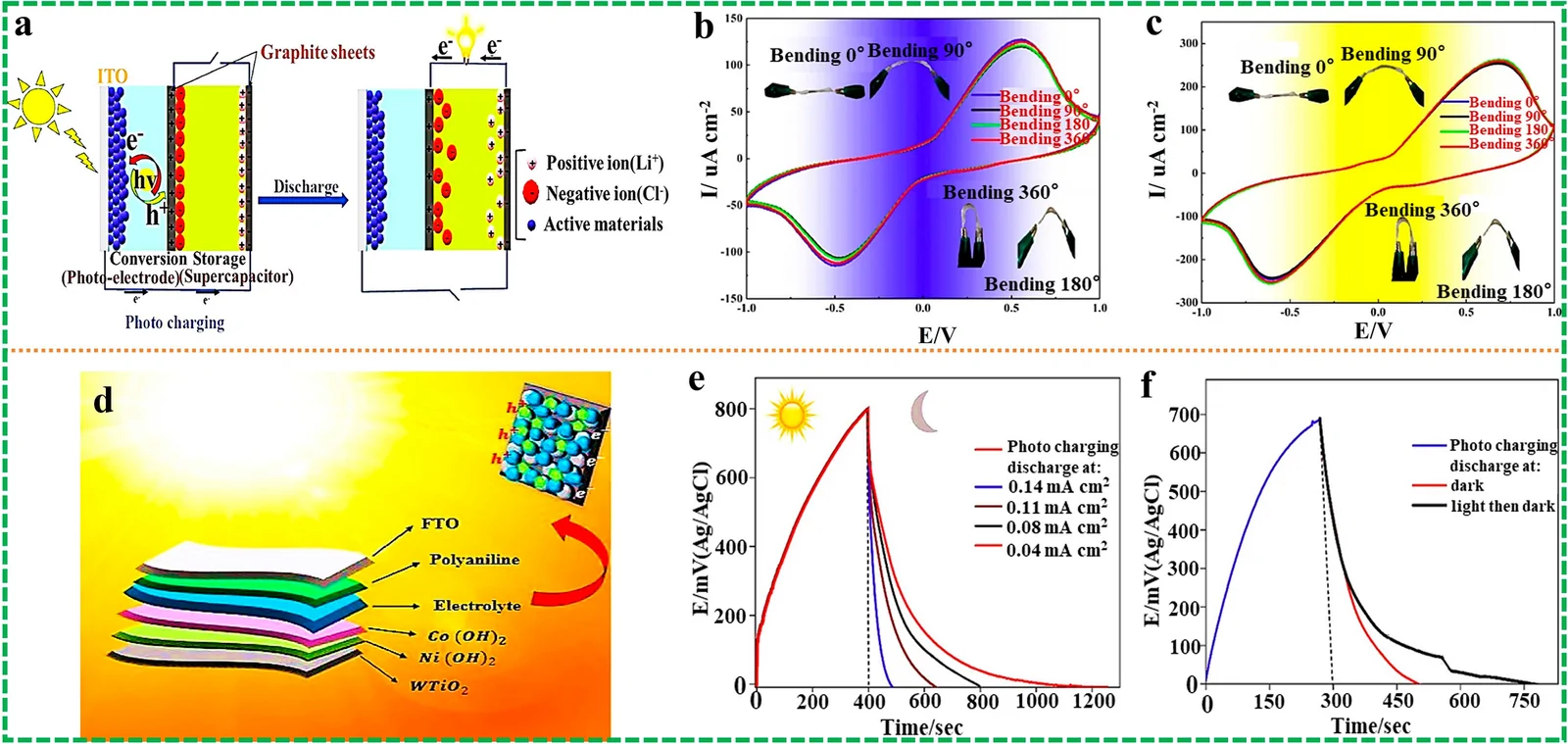
**a** A schematic illustration of the photo-charge and discharge mechanism in self-powered photo-SCs. Comparison of CV of assembled device in four bending states **b** in the dark and **c** under light illumination. Reproduced with permission [165]. Copyright 2023, Elsevier Ltd. **d** NiCo@WTs electrodes for photo-assisted rechargeable SCs are prepared. **e** Photo-charge for 400 s and galvanostatic discharge at different current densities. **f** Photo-charge and galvanostatic discharge at 0.13 mA cm^−2^ in dark and illuminated conditions. Reproduced with permission [195]. Copyright 2023, Elsevier B.V

Building upon these metallic approaches, metal-doped transition metal oxides integrated with conductive polymer composites represent promising avenues for enhancing both photoelectrochemical performance and mechanical flexibility. Momeni et al. developed innovative flexible lightweight SCs utilizing Se-V_2_O_5_-PPy composite electrodes through a simple, rapid, and economical process specifically targeting flexible applications [165]. The symmetric flexible all-solid-state SC exhibits enhanced energy storage under illumination due to increased photogenerated electron injection, with the device maintaining functionality under various bending conditions and demonstrating enhanced capacitive performance under illumination (Fig. 12a-c). Successfully powering LEDs and motor fans while flexed, these flexible SCs validate their superior electrode performance in a mechanically dynamic environment, with this scalable manufacturing approach holding significant potential for practical applications in flexible lightweight photo-assisted SCs.

Binary metal oxides and hydroxides demonstrate superior electrochemical properties compared to single-component systems through multiple oxidation states and enhanced conductivity pathways. NiCo-based oxide/hydroxide nanostructures have garnered particular attention due to their unique properties, including flexible ion exchange capabilities, excellent redox activity, cost-effectiveness, and environmental compatibility [196]. The co-existence of cobalt and nickel ions, operating within similar potential windows, provides multiple redox reaction sites, significantly enhancing electrochemical activity, while the formation of highly conductive CoOOH during electrochemical processes further improves overall material conductivity. Titanium dioxide nanotubes (TNTs) have emerged as ideal photosensitive materials for solar cell applications, owing to their exceptional optical and chemical stability, non-toxicity, corrosion resistance, and economic viability. Their functional characteristics and highly controllable morphology establish their crucial role in nanodevices [197]. The strategic integration of these materials with tungsten-doped TiO_2_ nanotube substrates through controlled electrodeposition creates breakthrough photorechargeable SC architectures, leveraging the exceptional optical and chemical stability, non-toxicity, corrosion resistance, and economic viability of titanium dioxide nanotubes (Fig. 12d) [195]. Optimized NiCo-10 electrodes exhibit superior capacitances of 252.4 mF cm^−2^, representing tenfold increases compared to bare substrates, with 68.3% capacitance enhancement under illumination achieving maximum photoelectric areal capacitances of 75.2 mF cm^−2^ at current densities of 0.04 mA cm^−2^ (Fig. 12e). This performance corresponds to power densities of 7.54 mW cm^−2^ and areal energy densities of 33.93 mWh cm^−2^, with notably slower discharge rates under illumination attributed to residual photocharges retained during the discharge process (Fig. 12f). This research presents the first demonstration of photorechargeable SCs utilizing NiCo@WTs as active materials, enabling direct light energy charging without external photovoltaic devices and establishing exceptional potential in photo-assisted rechargeable SC energy storage applications.

**Fig. 13:**
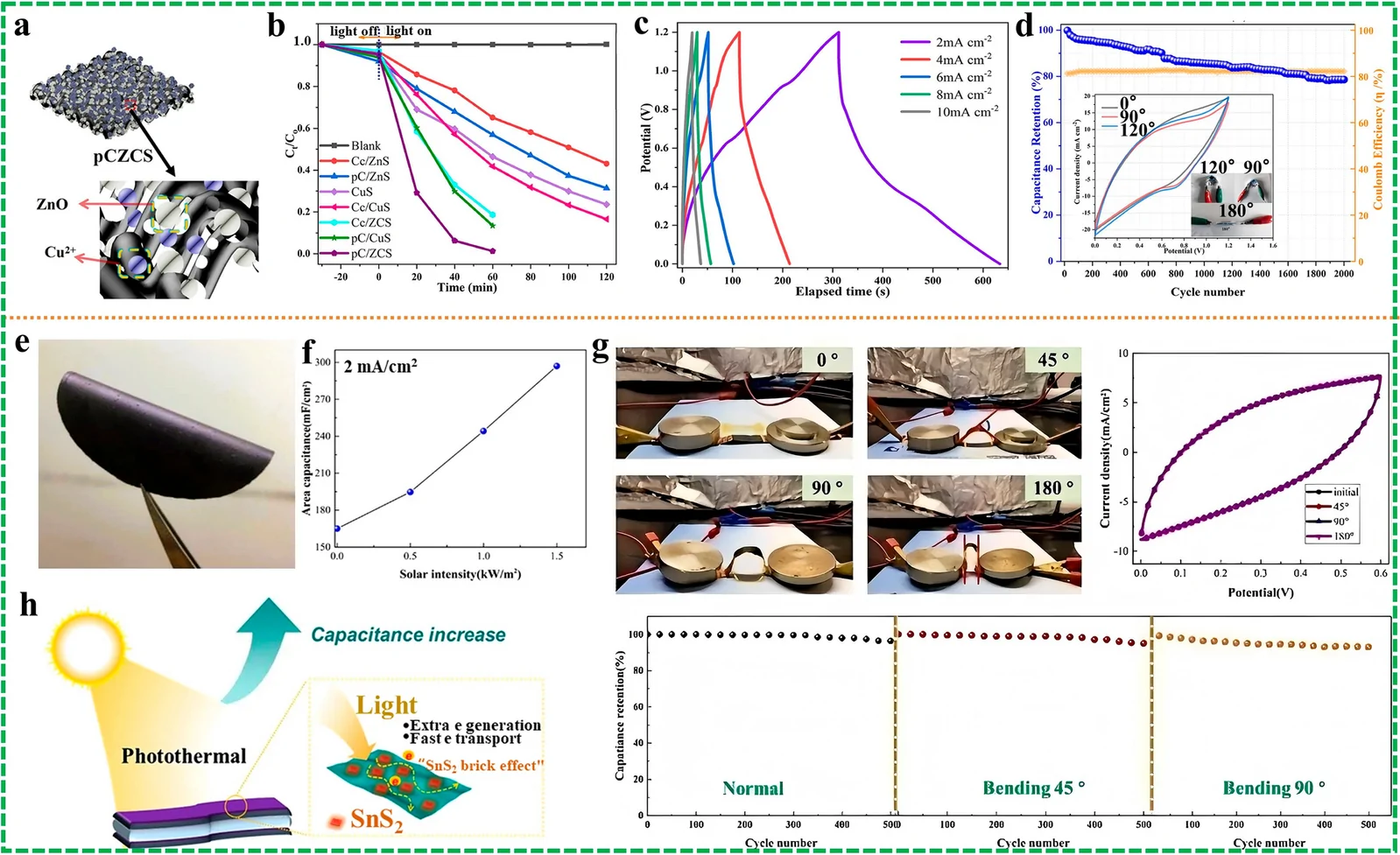
**a** Structure diagram of pCZCS. **b** Photocatalytic activities of the samples as prepared. **c** GCD curves of SC based on pCZCS electrode. **d** Capacitance retention of SC based on pCZCS electrode after 2000 cycles, the inset is the CV curves under various bending angles. Reproduced with permission [201]. Copyright 2020, Academic Press Inc. **e** Optical image of flexible MXene nacre. **f** Area capacitance of device at current density of 2 mA cm^−2^ under different solar intensity. **g** Electrochemical properties of flexible device. **h** Schematic illustration of the solar enhanced capacitance of the ASC. Reproduced with permission [202]. Copyright 2021, Elsevier

Recent advances in transition metal sulfide systems, attributed to their multiple valence states, controllable morphology, moderate band gaps, and natural abundance, demonstrate exceptional potential for flexible photo-assisted applications [198]. Copper sulfide and zinc sulfide have emerged as exemplary materials for high-performance electrode and photocatalyst design, demonstrating remarkable application potential [199, 200]. Novel heterostructured materials comprising porous carbonized cotton (an inherently flexible and conductive substrate), zinc sulfide, and copper sulfide (pCZCS) have been developed through specific methodologies, exhibiting both enhanced photocatalytic efficiency and high energy density (Fig. 13a) [201]. Research demonstrates that ZnS/CuS heterostructures effectively reduce photogenerated electron–hole pair recombination rates, while synergistic interactions among ZnS, CuS, and porous carbon facilitate efficient photogenerated carrier separation under illumination, significantly enhancing photocatalytic efficiency (Fig. 13b). In SC applications, the combination of ZnS/CuS exhibiting substantial pseudocapacitive characteristics with porous carbon materials demonstrating excellent double-layer capacitance properties endows the composite electrode with exceptional electrochemical performance. Experimental results reveal specific capacitances reaching 1925 mF cm^−2^ at current densities of 4 mA cm^−2^, with symmetric flexible SCs constructed using these composite electrodes achieving energy densities of 0.39 Wh cm^−2^ at power densities of 4.32 W cm^−2^ (Fig. 13c). Furthermore, the electrode demonstrates excellent flexibility and cycling stability (Fig. 13d), making it suitable for portable or wearable electronic device flexible energy storage systems. Benefiting from these synergistic effects, the fabricated pCZCS combines efficient photocatalytic performance with high-energy density storage characteristics, presenting significant implications for advancing interdisciplinary innovation and development.

MXenes demonstrate exceptional potential in high-performance SC development, attributed to their outstanding metallic conductivity and high specific capacitance [203, 204]. Inspired by the natural “brick-and-mortar” architecture of nacre, Fu et al. [202] presented an innovative yet straightforward approach to developing geometrically flexible and mechanically robust MXene (Ti_3_C_2_T_x_) films with superior energy storage capabilities and efficient photothermal conversion properties (Fig. 13e). The in-situ growth of two-dimensional SnS_2_ on MXene nanosheets not only enhances photothermal conversion efficiency but also effectively suppresses restacking phenomena. This biomimetic MXene “nacre-like” structure exhibits exceptional mechanical strength (78.3 MPa) and ultra-flexibility, maintaining 91.5% capacitance retention after 4000 cycles and demonstrating remarkable flexibility and cycling stability with > 90% capacitance retention after 500 folding/unfolding cycles (Fig. 13g). Under solar illumination of 1 kW m^−2^, the flexible MXene SC demonstrates a 60% increase in capacitance (Fig. 13f), attributed to photothermal effects enhancing ion/electron conduction through electron excitation from valence band to conduction band, generating electron–hole pairs and consequently reducing resistance (Fig. 13h). This research establishes novel pathways for developing flexible all-solid-state MXene photothermal SCs, showing promising applications in structured flexible energy storage devices and validating the potential for biomimetic architectural approaches in flexible photoelectrochemical energy storage systems.

The escalating demand for renewable energy has catalyzed significant advancements in photo-assisted flexible SC research through strategic optimization of photoelectrode materials suitable for flexible architectures and the integration of emerging materials as photoactive components. The continuous emergence of innovative flexible device architectures and fabrication methodologies has provided robust technical foundations for practical implementation. These systematic advances demonstrate the increasing maturity of photo-assisted flexible SC technology, collectively addressing fundamental challenges at the intersection of photoelectrochemical functionality and mechanical deformability while establishing robust foundations for next-generation portable energy systems. Future developments should focus on scalable manufacturing methodologies, enhanced environmental stability, and integrated multi-functional capabilities to fully realize the potential of photo-assisted flexible SCs in addressing contemporary energy storage challenges where mechanical adaptability and photoelectrochemical enhancement are critical performance requirements for wearable electronics, smart textiles, and distributed energy applications.

### Photo-Assisted Flexible Lithium Batteries

Photo-assisted flexible lithium batteries represent a transformative approach to addressing the dual challenges of energy density enhancement and mechanical adaptability in next-generation portable energy systems. These devices strategically integrate photoelectrochemical processes with lithium-based electrochemistry to achieve superior performance while maintaining mechanical flexibility essential for wearable electronics and conformable energy storage applications [205]. The fundamental design paradigm centers on leveraging photogenerated carriers to enhance reaction kinetics, reduce overpotentials, and improve energy conversion efficiencies within mechanically deformable device architectures. Critical integration challenges include preserving photoelectrochemical interfaces under mechanical stress, maintaining electrolyte stability during deformation cycles, and optimizing light utilization across various geometric configurations while ensuring long-term electrochemical performance [9, 206].

#### Photo-Assisted Flexible Lithium-Oxygen Batteries

Photo-assisted lithium-oxygen batteries represent an innovative convergence of photoelectrochemical energy conversion with high-energy density electrochemical storage, particularly promising for flexible applications where mechanical adaptability is essential [159]. The integration of photoactive materials within flexible cathode architectures—including coating on flexible carbon cloth substrates or embedding in flexible polymer binders—enables photogenerated carrier participation in oxygen reduction and evolution reactions while maintaining mechanical conformability [207]. However, implementation introduces unique challenges including high charge overpotentials exacerbated by mechanical stresses, potential interface degradation under deformation, and the fundamental issue of insulating Li_2_O_2_ formation leading to significant overpotentials that can compromise long-term stability in flexible cells [208].

**Fig. 14:**
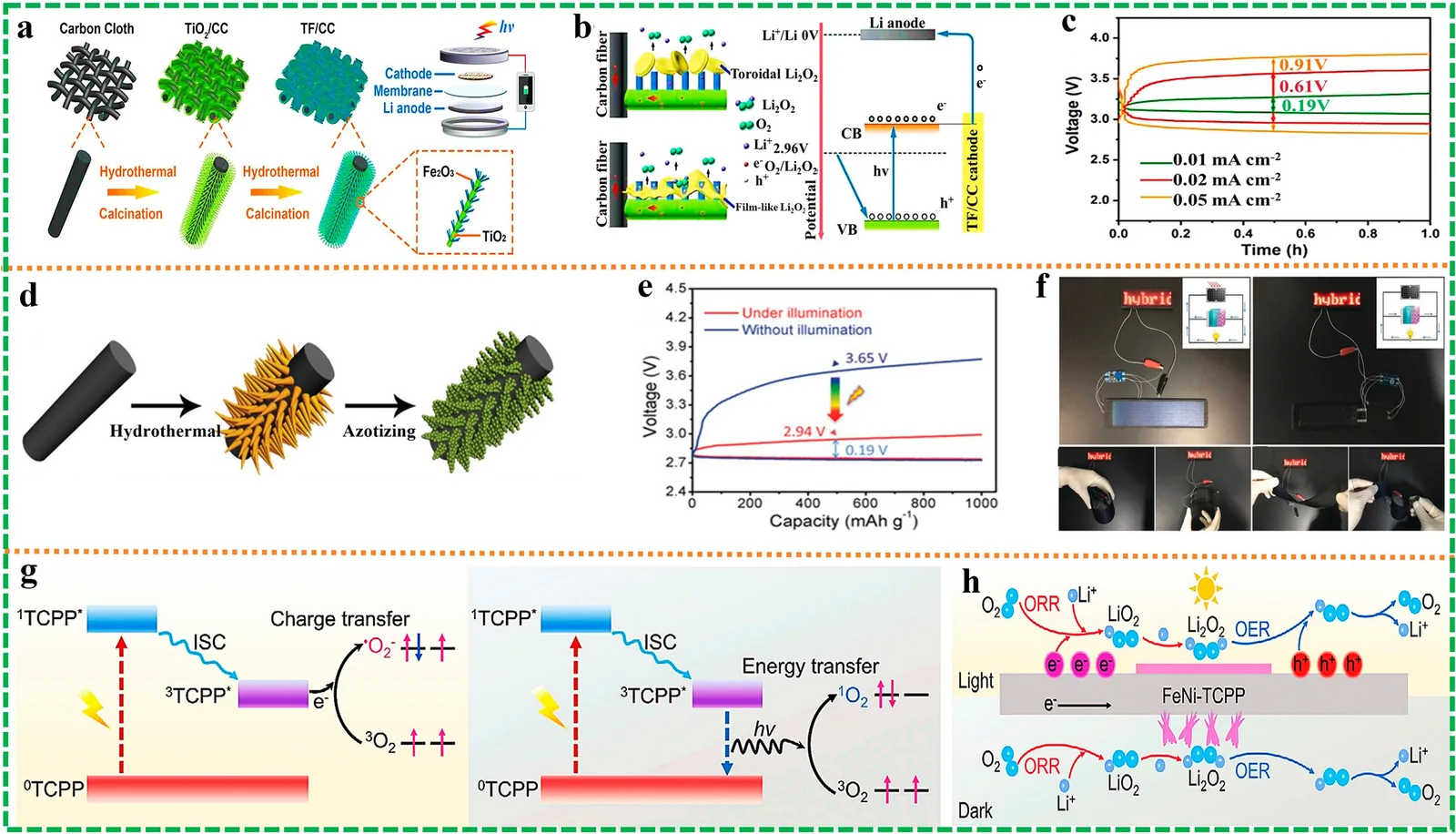
**a** Schematic illustration of the synthesis procedure for the TF/CC cathode and the structure of the photo-assisted Li–O_2_ battery. **b** Schematic illustration of the charging process with and without illumination and the proposed photo-electrochemical process in the TF/CC cathode during charging. **c** Galvanostatic charge–discharge profiles of the Li–O_2_ batteries at different current densities with illumination. Reproduced with permission [168]. Copyright 2020, Wiley-Blackwell. **d** Representations of the design and preparation of the TT@CC cathode. **e** Discharge–charge curves of the Li–O_2_ batteries with or without illumination. **f** Digital photograph of the assembled self-powered energy system working with or without the light: Inset shows the working circuit connection; the self-powered energy system powering a commercial red LED display screen at various bent and twisted conditions. Reproduced with permission [79]. Copyright 2019, John Wiley and Sons Ltd. **g** Schematic generation of ^•^O_2_^−^ and ^1^O_2_. **h** ORR and OER of FeNi-TCPP with and without irradiation. Reproduced with permission [210]. Copyright 2024, Wiley-Blackwell

TiO_2_ has emerged as a promising photocatalyst foundation due to its exceptional chemical stability, non-toxicity, and photocorrosion resistance, making it particularly suitable for flexible battery applications [208]. Its wide bandgap limitations are effectively addressed through heterojunction construction with semiconductors like n-type hematite (Fe_2_O_3_), enabling strategic bandgap engineering for enhanced visible light utilization [209]. A breakthrough bifunctional photo-assisted Li–O_2_ battery was developed through TiO_2_–Fe_2_O_3_ heterojunction structures on flexible carbon cloth substrates (Fig. 14a), where photogenerated electrons and holes reduce overpotentials during discharge and charge processes respectively [168]. Under illumination, uniform film-like Li_2_O_2_ structures develop between catalyst arrays, while photo-excited holes facilitate Li_2_O_2_ decomposition during charging (Fig. 14b), achieving ultra-low overpotentials of 0.19 V with 86% round-trip efficiency maintained after 100 cycles (Fig. 14c). Advanced TiN/TiO_2_ composite nanowires in situ grown on carbon cloth (TT@CC) further demonstrate flexible integration potential, achieving 94% energy conversion efficiency under solar illumination with charging overpotentials reduced to 0.19 V (Fig. 14d, e) [79]. Integration with commercial solar cells creates comprehensive flexible self-powered energy systems capable of continuously powering LEDs while maintaining consistent electrochemical performance under various bending conditions (Fig. 14f), validating practical viability for flexible and wearable electronic applications.

MOFs, characterized by periodic network structures formed through self-assembly of metal ions with bridging organic ligands, offer unique opportunities for flexible battery integration through their tunable molecular architectures and distinctive optical properties [170, 210, 211]. Porphyrin-based MOFs, constructed through coordination-driven self-assembly, exhibit exceptional photoelectrochemical properties where porphyrin ligands absorb photons, inducing electronic transitions that generate excited-state electrons for efficient charge separation through ligand-to-metal cluster charge transfer (LMCT) processes [212–214]. Groundbreaking research demonstrated porphyrin metal–organic frameworks containing (Fe_2_Ni)O(COO)_6_ clusters on flexible carbon cloth substrates as photocathodes, strategically designed to accelerate exciton dissociation and enhance carrier transport [210]. Under visible light irradiation, ground-state TCPP photosensitizer transitions through intersystem crossing to generate singlet oxygen while facilitating charge separation through enhanced LMCT effects (Fig. 14g). The photo-assisted lithium-oxygen battery achieves remarkably low total overpotentials (0.28 V) and 92% round-trip efficiency under illumination. The enhancement mechanism involves TCPP linker generation of electron–hole pairs, efficient charge separation at Ni sites, and subsequent carrier transfer to O_2_ molecules forming •O_2_^−^ radicals that combine with Li^+^ to form LiO_2_ (Fig. 14h). During charging, photogenerated holes actively participate in Li_2_O_2_ oxidation, promoting decomposition and significantly reducing charging overpotentials. The utilization of flexible carbon cloth substrates establishes promising approaches for flexible photo-assisted Li–O_2_ battery development.

Sustained research efforts in photo-assisted Li–O_2_ batteries have yielded remarkable advancements through strategic integration of innovative materials and structural designs compatible with flexible device requirements. Implementation of efficient photoelectrode materials, heterojunction architectures, bifunctional catalysts, and MOF systems has successfully reduced overpotentials from 2.96 V to below 2 V while achieving substantial enhancements in specific capacity, rate capability, and cycling stability, particularly impactful in flexible photo-assisted Li–O_2_ battery development utilizing flexible substrates like carbon cloth and nanostructured catalysts.

Future research directions should prioritize comprehensive optimization of energy storage and conversion efficiencies through advanced material design compatible with flexible manufacturing processes, robust interface engineering strategies to withstand repetitive mechanical stress, and innovative system integration approaches tailored for wearable and conformable device applications. Critical development areas include scalable synthesis of flexible photoelectrode materials, enhanced environmental stability under mechanical cycling, and integrated multi-functional capabilities that leverage both photoelectrochemical enhancement and mechanical adaptability for next-generation energy storage technologies in wearable electronics, smart textiles, and distributed energy applications.

#### Photo-Assisted Flexible Lithium-Carbon Dioxide Batteries

Rechargeable lithium-carbon dioxide batteries demonstrate remarkable theoretical energy density, presenting significant potential for flexible energy storage applications [215]. However, the implementation of flexible photo-assisted Li–CO_2_ batteries introduces complex integration challenges including requirements for mechanical stability of electrode and electrolyte components under deformation, preservation of photoelectrochemical interfaces during flexing cycles, and maintenance of gas permeability through mechanically stressed membranes. For flexible Li–CO_2_ battery configurations, fundamental electrochemical challenges are compounded by the need to maintain performance under mechanical deformation while ensuring interface stability [216–218]. Notable cathode catalysts such as SiC@RGO [18], CNT@C_3_N_4_ [219], and TiO_2_/CC [220] have demonstrated promising performance. The introduction of photo-assisted mechanisms represents a promising approach for flexible implementations, though several critical challenges persist: (1) limited photon utilization efficiency particularly problematic in flexible geometries with variable light exposure; (2) insufficient understanding of electron–hole separation/transfer mechanisms under mechanical strain in flexible cells; (3) limited active site utilization and precise structural control necessary for flexible device optimization. Consequently, there exists an urgent need for designing photosensitive cathode catalysts with mechanical flexibility to enhance photo-assisted processes while maintaining performance under deformation.

**Fig. 15:**
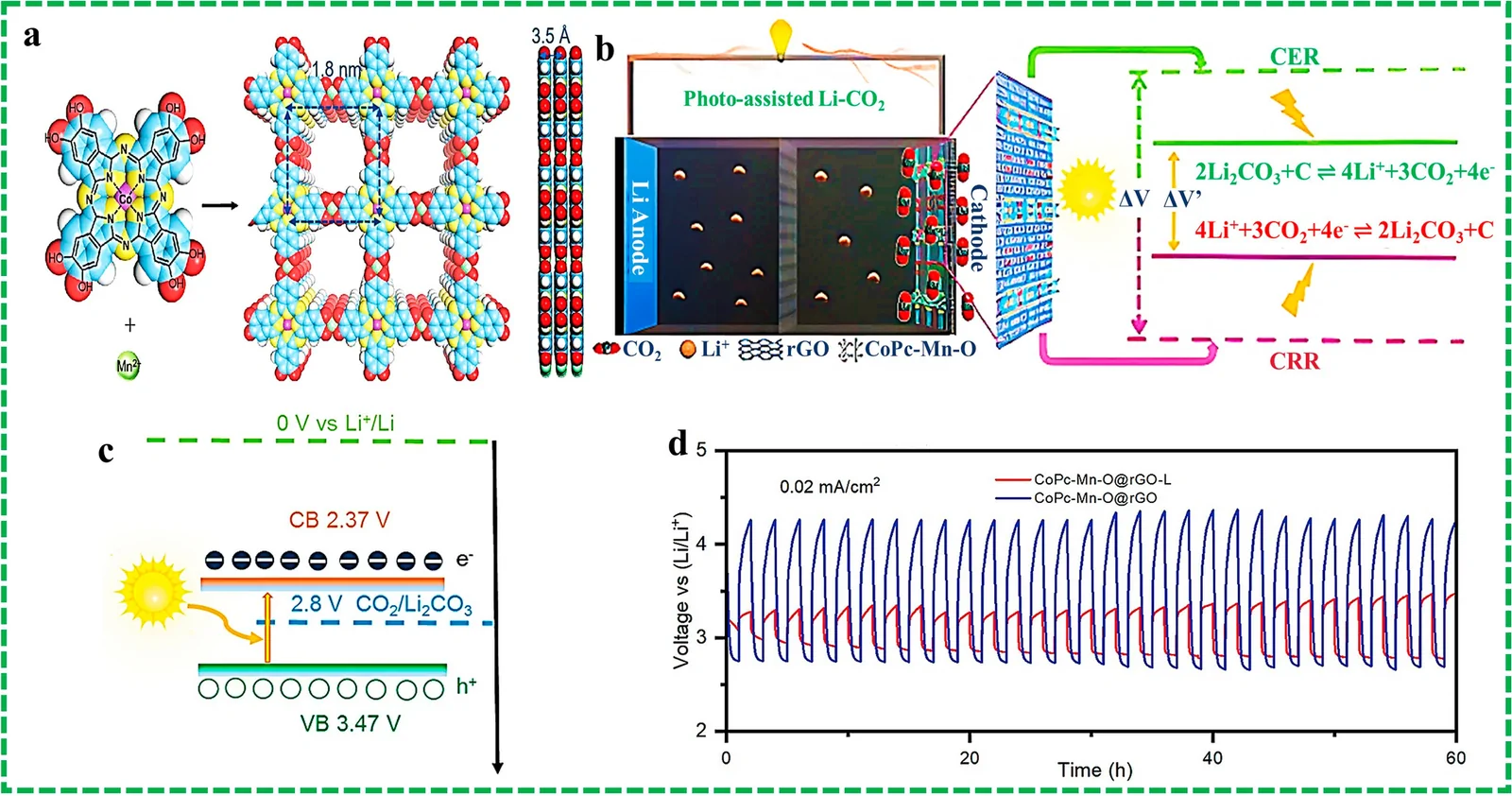
**a** Design and synthetic scheme for CoPc-Mn–O. **b** Structure and principle of light-assisted Li–CO_2_ lithium battery. **c** Energy diagram of CoPc-Mn–O and standard potential of CO_2_/Li_2_CO_3_ versus Li^+^/Li. **d** Voltage profiles of typical Li–CO_2_ battery cycled with 0.01 mAh cm^−2^ cutoff areal capacity with and without illumination for CoPc-Mn–O@rGO-L and CoPc-Mn–O@rGO. Reproduced with permission [171]. Copyright 2022, Wiley–VCH Verlag

MOFs demonstrate remarkable versatility for flexible energy device integration through their inherent porosity, tunable optical properties, and compatibility with flexible substrates [221–227]. In the context of photo-assisted flexible Li–CO_2_ batteries, MOF materials demonstrate immense potential through their adaptability to flexible device architectures. Innovative research demonstrated phthalocyanine-based metal–organic framework nanosheets (CoPc–Mn–O) as efficient photoelectrocatalysts, with the material supported on reduced graphene oxide to form conductive and flexible carbon films suitable for flexible battery assembly (Fig. 15a, b) [171]. The distinctive characteristics include nanosheet morphology (approximately 1 nm thickness optimal for flexible substrate integration), high conductivity essential for flexible device performance, and broad-spectrum photosensitivity. These properties collectively enhance battery efficiency while maintaining mechanical flexibility through the rGO support matrix that provides both electrical conductivity and mechanical resilience necessary for flexible battery applications. CoPc–Mn–O satisfies fundamental requirements for photo-assisted rechargeable Li–CO_2_ batteries, with the CO_2_/Li_2_CO_3_ redox potential (2.80 V vs Li^+^/Li) positioned between its CB and VB potentials (Fig. 15c). This band alignment enables efficient utilization of photoelectrons and holes for solar energy conversion and storage, facilitating enhanced photo-assisted battery performance through Li_2_CO_3_ oxidation. Experimental validation demonstrates performance metrics that translate effectively to flexible configurations: superior round-trip efficiency reaching 98.5%, ultra-low voltage hysteresis of 0.05 V, and excellent cycling stability maintaining 81.3% capacity retention over 60 h at 0.02 mA cm^−2^ current density (Fig. 15d). The inherent flexibility of the rGO-supported MOF nanosheet electrode architecture represents a significant advancement toward practical flexible Li–CO_2_ battery implementation, demonstrating that high-performance photoelectrochemical functionality can be maintained within mechanically adaptable device configurations.

This development establishes new paradigms for flexible photo-assisted battery technologies, with the successful integration of MOF nanosheets with flexible carbon supports demonstrating the feasibility of achieving high-performance photo-assisted Li–CO_2_ batteries while maintaining mechanical adaptability essential for wearable electronics and conformable energy systems. Future research directions should focus on advancing scalable synthesis methodologies for flexible MOF-based photoelectrodes, developing robust encapsulation strategies to maintain gas permeability under mechanical stress, and optimizing device architectures that maximize both photoelectrochemical efficiency and mechanical resilience. Critical development priorities include enhancing long-term stability under repetitive mechanical cycling and developing integrated manufacturing processes compatible with flexible substrate requirements for advanced portable and wearable energy systems.

#### Photo-Assisted Flexible Lithium–Sulfur Batteries

Lithium–sulfur batteries have emerged as promising alternatives for flexible energy storage application, offering exceptional theoretical specific capacity and high energy density, while presenting unique integration challenges for flexible device architectures [228]. The fundamental challenge of polysulfide shuttle effects is significantly compounded in flexible configurations, where mechanical deformation can exacerbate polysulfide dissolution, compromise electrical contacts, and destabilize electrode–electrolyte interfaces during flexing cycles. Recent advancements in integrating solar energy into flexible Li–S systems leverage photovoltaic, photothermal, and photocatalytic effects to address these challenges while enabling mechanically adaptable device architectures [229]. Solar energy integration effectively ameliorates sluggish kinetics and enhances battery performance, particularly beneficial for flexible systems where conventional rigid catalysts may not be suitable and where mechanical stress can further impact conductivity [229].

**Fig. 16:**
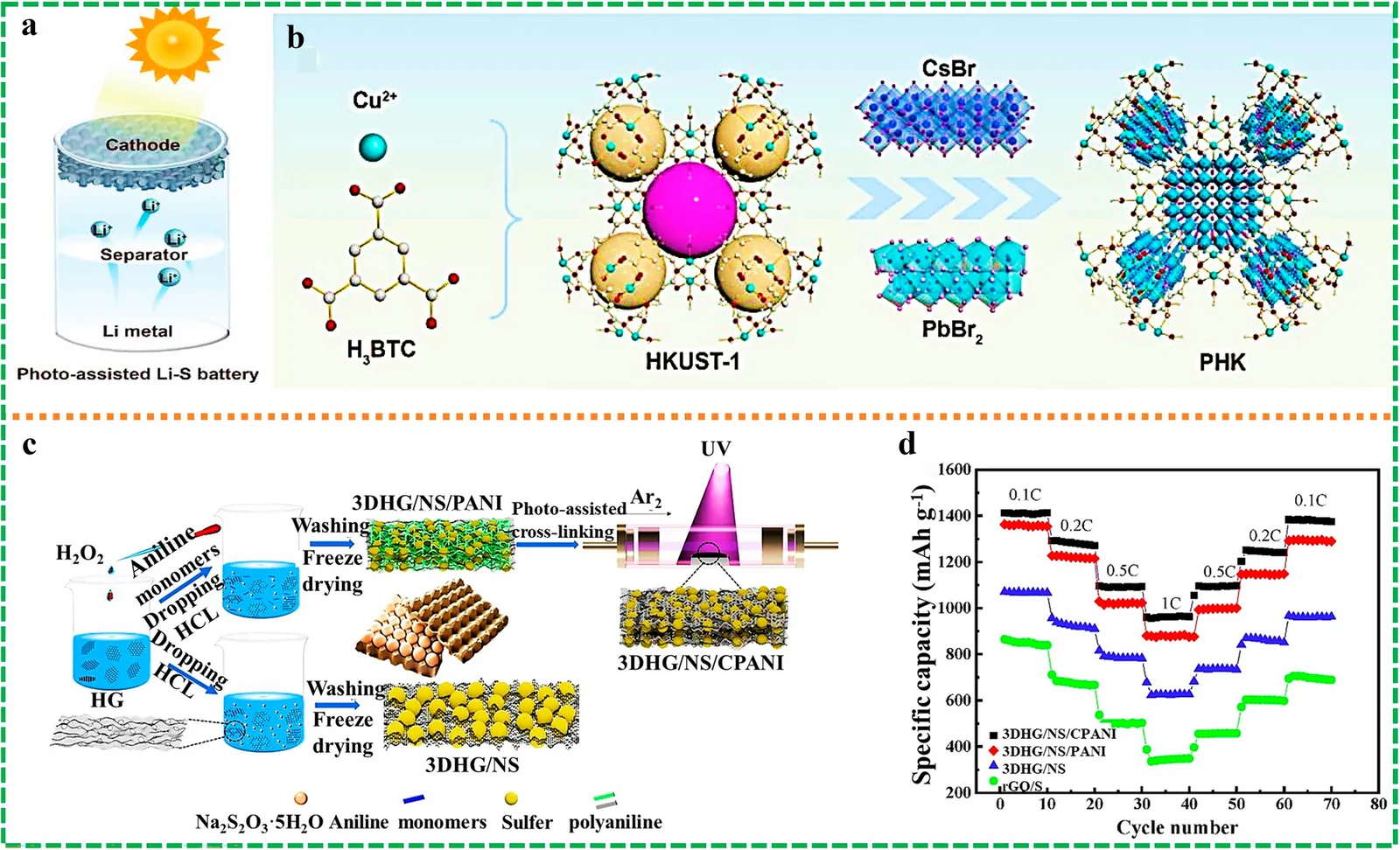
**a** Illustration of PALSB. **b** Schematic illustration of the synthesis of PHK. Reproduced with permission [172]. Copyright 2024, John Wiley and Sons Ltd. **c** Preparation diagram of 3DHG/NS/CPANI composite. **d** Rate performance of 3DHG/NS/CPANI,3DHG/NS/PANI, 3DHG/NS and RGO/S cathodes at 0.1, 0.2, 0.5, and 1 C. Reproduced with permission [173]. Copyright 2021, Elsevier Ltd

Strategic materials integration for flexible photo-assisted Li–S batteries (Fig. 16a) focuses on developing mechanically resilient photoactive catalysts that maintain performance under deformation. Mixed-phase CsPbBr_3_/Cs_4_PbBr_6_ perovskite quantum dots deposited on metal–organic framework HKUST-1 create structurally stable composite materials (PHK) particularly suitable for flexible applications when assembled with carbon paper electrodes (Fig. 16b) [172]. The precise ordered microstructure provides spatial confinement while generating built-in electric fields at composite interfaces, effectively promoting photogenerated electron–hole separation and extending carrier lifetime under mechanical stress. This flexible PHK catalyst system achieves precise polysulfide regulation, demonstrating excellent rate capabilities across broad current density ranges (0.2 to 5 C) with minimal capacity decay of 0.022% per cycle after 1500 stable cycles at 5 C. Under high sulfur loading conditions, the flexible Photo-assisted lithium–sulfur batteries (PALSB) achieves impressive areal capacity of 6.4 mAh cm^−2^, indicating substantial potential for flexible energy storage applications.

Three-dimensional porous graphene frameworks demonstrate exceptional promise for flexible photo-assisted Li–S batteries through their large surface area, superior conductive networks, and mechanical stability enabling effective accommodation of volume changes during flexing [230]. Advanced “egg-plate” layered structures incorporating nanosulfur uniformly distributed within polyaniline-crosslinked three-dimensional hierarchical graphene frameworks (3DHG/NS/CPANI) represent breakthrough developments for flexible high-loading LSB cathodes (Fig. 16c) [173]. The photo-assisted crosslinking methodology creates efficient polysulfide adsorbents that enable firm nanosulfur embedding within graphene pores through both physical and chemical interactions, effectively preventing polysulfide dissolution and shuttle effects even under mechanical deformation. The engineered flexible 3DHG/NS/CPANI electrodes exhibit exceptional cycling stability with discharge specific capacities reaching 1082 and 921 mAh g^−1^ at 0.5 and 1 C rates respectively (Fig. 16d), maintaining minimal capacity decay of 0.04% per cycle over 500 cycles.

**Fig. 17:**
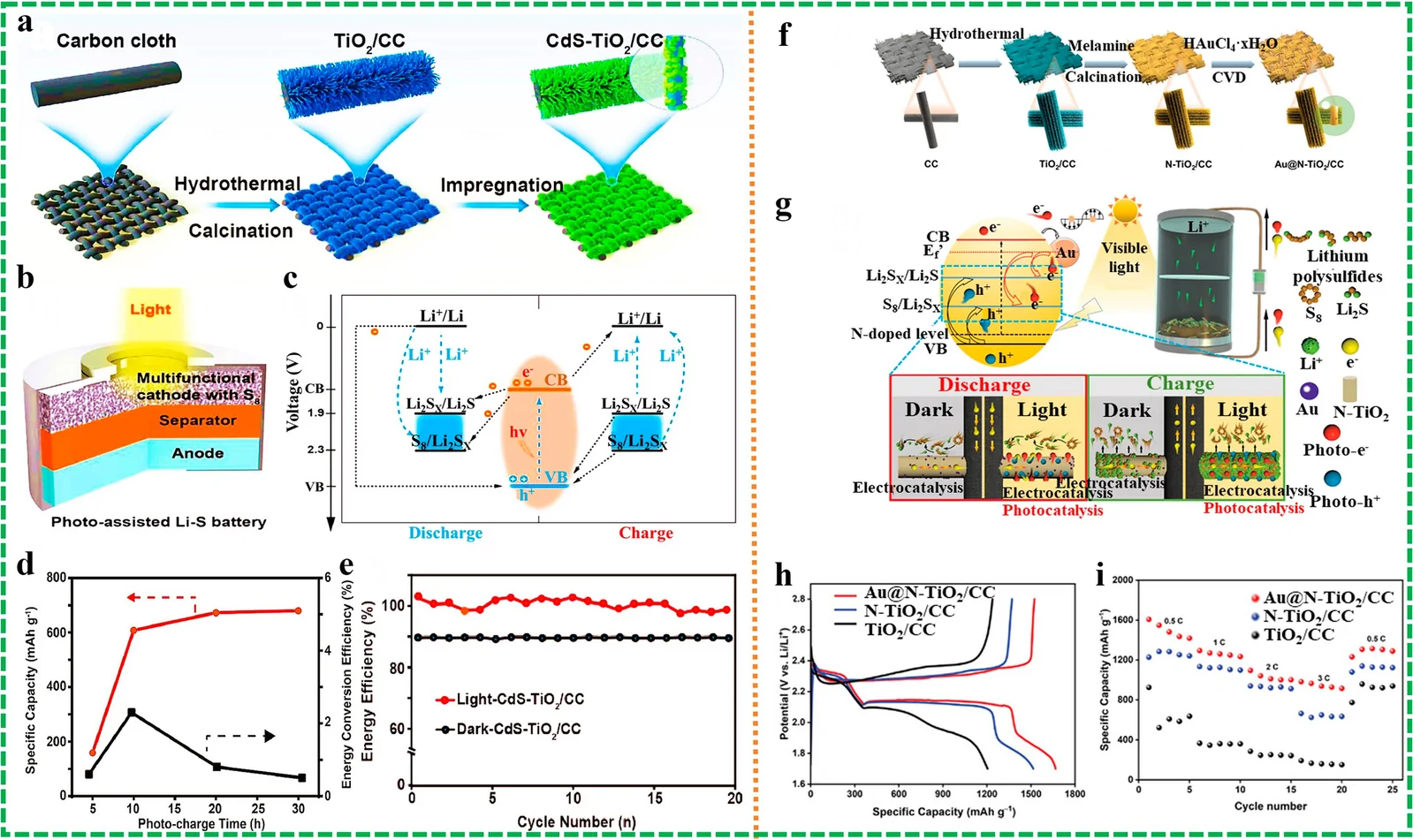
**a** Schematic illustrating the synthesis of CdS-TiO_2_/CC. **b** Illustration of photo-assisted Li–S battery. **c** Charge transfers of photo-generated electrons and holes in the photo-assisted Li–S battery under a solar light illumination. **d** Discharge capacities and energy conversation efficiencies with different illumination time. **e** Energy efficiencies of the CdS-TiO_2_/CC batteries with and without the illumination. Reproduced with permission [174]. Copyright 2022, Elsevier. **f** Schematic illustrating the fabrication progress of Au@N-TiO_2_/CC photoelectrode. **g** Catalytic mechanism of an Au@N-TiO_2_/CC photoelectrode in a PALSB. **h** GCD curves of PALSB with various photoelectrodes under illumination at 0.1 C. **i** Rate performance of PALSB with various photoelectrodes under illumination at 0.1 C. Reproduced with permission [231]. Copyright 2024, Wiley

Advanced heterostructure integration demonstrates significant potential for flexible photo-assisted Li–S battery development through CdS–TiO_2_ systems synthesized on flexible carbon cloth as multifunctional cathode collectors, accelerating both sulfur reduction and evolution reactions while maintaining mechanical adaptability (Fig. 17a–c) [174]. Under solar illumination, the flexible photo-assisted LSB system achieves direct photochemical charging capabilities with specific discharge specific capacity of 608 mAh g^−1^ and remarkable energy conversion efficiency of 2.3%, representing approximately 36.3% of theoretical capacity (Fig. 17d). The system demonstrates stable reversible specific capacity of about 1225 mAh g^−1^ with 100% energy efficiency, showing 10% improvement compared to non-illuminated conditions while maintaining performance under mechanical deformation (Fig. 17e).

Standalone photoelectrode development through Au@N–TiO_2_ heterojunction construction on flexible carbon cloth further validates flexible PALSB potential, demonstrating exceptional photon capture capabilities and efficient electron–hole separation rates (Fig. 17f, g) [231]. The flexible photoelectrode generates photoelectrons conducive to sulfur reduction during discharge while producing holes that accelerate sulfur evolution during charging, achieving remarkable active material utilization and exceptional specific capacity of 1667 mAh g^−1^ (Fig. 17h). Light-induced carrier generation increases free electron concentration within the flexible battery system, reducing resistance and improving electrochemical kinetics, resulting in superior rate performance (982 mAh g^−1^ at 3 C) (Fig. 17i).

The integration of advanced flexible materials including three-dimensional graphene frameworks and conformable MOF composites presents strategic opportunities for flexible photo-assisted Li–S battery optimization through: (1) flexible electron/ion transport pathways maintaining conductivity during deformation; (2) mechanically resilient polysulfide confinement; (3) enhanced photoelectrochemical conversion in bendable architectures; (4) strain-tolerant charge carrier separation; (5) stable flexible electrode architecture suitable for wearable and conformable applications. Future development priorities should focus on advancing scalable synthesis methodologies for flexible photoactive materials, developing robust encapsulation strategies to prevent polysulfide leakage under mechanical stress, and optimizing device architectures that maximize both photoelectrochemical efficiency and mechanical resilience for next-generation flexible energy storage systems where high capacity, rapid kinetics, and mechanical adaptability are critical requirements.

#### Photo-Assisted Flexible Lithium-Nitrogen Batteries

Photo-assisted lithium-nitrogen batteries represent an emerging frontier in flexible energy storage systems, leveraging the dual potential of energy storage and nitrogen utilization within mechanically adaptable device architectures [232]. The development of flexible Li–N_2_ batteries addresses unique integration challenges stemming from nitrogen’s exceptional chemical inertness, which results in substantial overpotentials exceeding 2 V and limited energy utilization efficiency—challenges further compounded by requirements for mechanical resilience and interface stability under deformation. While various catalysts including graphene, Mo_2_C/NC, and NCNTs have been developed to improve cathode reaction kinetics, overpotentials remain significantly high, presenting particular challenges for flexible implementations where mechanical stress can further impact electrochemical performance [233–235].

The integration of renewable solar energy into flexible Li–N_2_ battery systems emerges as an effective strategy to overcome high overpotential challenges while enabling mechanical adaptability essential for wearable and conformable energy applications. However, the higher chemical inertness of nitrogen compared to oxygen or carbon dioxide imposes more stringent requirements on flexible photoelectric cathode design, necessitating advanced materials engineering approaches that simultaneously address electrochemical performance, photoelectrochemical enhancement, and mechanical flexibility requirements. The development of flexible photo-assisted Li–N_2_ batteries requires systematic consideration of materials selection, interfacial engineering, and device architecture optimization to achieve synergistic performance enhancement between photoelectrochemical functionality and mechanical resilience.

**Fig. 18:**
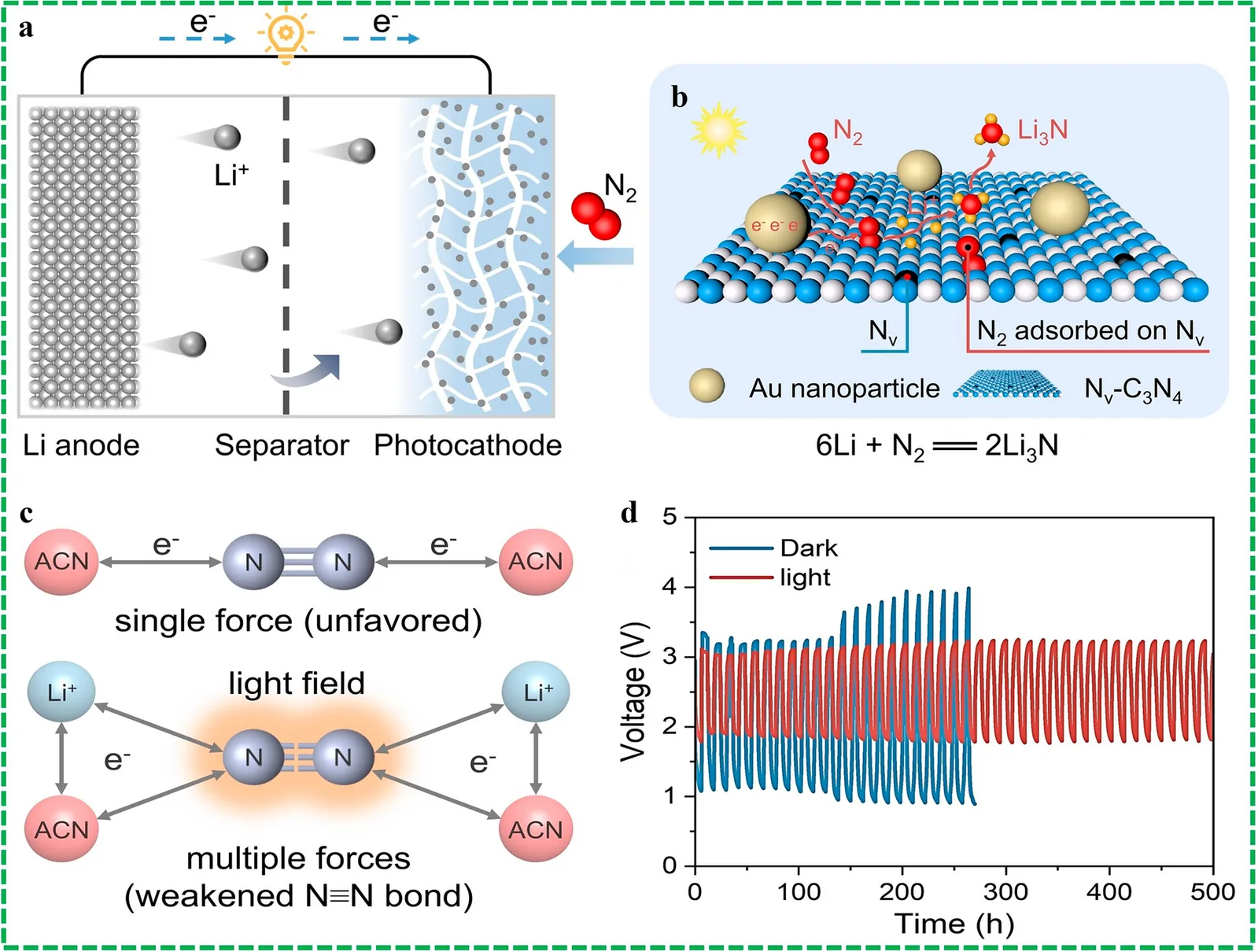
**a** Schematic of a photo-assisted Li–N_2_ battery with a Li anode, ether-based electrolyte, and photocathode. **b** Illustrations of the working mechanism of the photo-assisted Li–N_2_ battery with the Au–N_v_–C_3_N_4_ photocathode. **c** Schematic depiction of Li^+^, Au-N_v_-C_3_N_4_, light, and N_2_ interactions. ACN denotes the Au-N_v_-C_3_N_4_ catalyst. **d** Cycle performance of the Li–N_2_ battery under dark and light at a curtailing specific capacity of 600 mAh g^−1^ and a current density of 100 mAg^−1^. Reproduced with permission [176]. Copyright 2024, John Wiley and Sons Ltd

Carbon nitride (C_3_N_4_) demonstrates exceptional potential for flexible photo-assisted Li–N_2_ battery applications due to its unique electronic band structure, outstanding physicochemical stability, and superior functionalization capabilities suitable for flexible substrate integration [236]. However, inherent limitations including severe photogenerated carrier recombination and insufficient visible light absorption due to limited active sites result in relatively low photocatalytic activity, particularly challenging for flexible device configurations where light exposure may vary with mechanical deformation. Defect engineering techniques, particularly nitrogen vacancy (N_v_) introduction, offer effective strategies for enhancing C_3_N_4_’s photocatalytic selectivity and activity while maintaining structural integrity necessary for flexible applications [237]. Breakthrough developments in flexible photo-assisted Li–N_2_ battery systems utilize carbon cloth-supported, plasmonic gold nanoparticle modified defect-engineered carbon nitride (Au–Nv–C_3_N_4_) as photoelectric cathodes, demonstrating excellent compatibility with flexible device architectures (Fig. 18a, b) [176]. The Au-N_v_-C_3_N_4_ system achieves remarkable capabilities including enhanced photon capture, efficient N_2_ adsorption and activation, and accelerated discharge–charge reaction kinetics through photogenerated and hot electrons (Fig. 18c). These advantages enable flexible photo-assisted Li–N_2_ batteries to achieve unprecedented low overpotentials of 1.32 V—the lowest reported to date—while maintaining exceptional rate performance and cycling stability over 500 h under various mechanical configurations (Fig. 18d). The carbon cloth substrate provides inherent flexibility while maintaining electrical conductivity and structural integrity necessary for wearable energy storage applications. The Au–Nv–C_3_N_4_ flexible photoelectric cathode system demonstrates high reversibility and successfully addresses overpotential bottlenecks in Li–N_2_ battery systems while maintaining mechanical adaptability. This breakthrough significantly expands the application scope of photo-assisted batteries in flexible configurations and provides effective solutions to sluggish kinetics challenges specific to mechanically deformable Li–N_2_ battery systems. The integration of plasmonic enhancement with defect engineering creates synergistic effects that improve both photoelectrochemical performance and mechanical resilience, essential for practical flexible energy storage implementations.

Future research directions for photo-assisted flexible lithium batteries, particularly Li–N_2_ systems, should prioritize optimizing energy storage efficiency and energy density through strategic incorporation of photo-assisted designs within mechanically resilient architectures. Critical development areas include: (1) advanced photoelectric materials suitable for flexible substrates and manufacturing processes that maintain performance under mechanical stress; (2) novel flexible device architecture development that optimizes light utilization across various deformation states; (3) improved light-matter interaction mechanisms within deformable structures that accommodate varying geometric configurations; (4) enhanced charge carrier dynamics under mechanical stress that preserve photoelectrochemical efficiency; (5) optimized energy conversion pathways in flexible and wearable energy systems that integrate seamlessly with conformable electronics. These systematic developments are essential for realizing practical photo-assisted flexible Li–N_2_ batteries capable of addressing contemporary energy storage challenges where high performance, environmental sustainability, and mechanical adaptability are fundamental requirements for next-generation portable and wearable energy systems.

### Photo-Assisted Flexible Zinc Batteries

Photo-assisted flexible zinc batteries represent a promising approach to addressing the dual challenges of energy density enhancement and mechanical adaptability in next-generation portable energy systems. Zinc-based batteries offer impressive theoretical specific energy, substantially surpassing lithium-ion batteries, while providing abundant resources, cost-effectiveness, and superior safety characteristics essential for flexible applications [238, 239]. However, the implementation of flexible photo-assisted zinc batteries introduces unique integration challenges including high charging potentials typically exceeding 2 V, cathode degradation under elevated potential conditions, and the need to maintain photoelectrochemical interfaces under mechanical stress. For flexible zinc battery configurations, these fundamental electrochemical challenges are compounded by requirements for mechanical stability of electrode and electrolyte components under deformation, preservation of gas permeability in zinc-air systems during flexing cycles, and maintenance of ionic conductivity in zinc-ion systems across various mechanical states [240, 241].

Photo-assisted zinc batteries present effective solutions through solar energy integration as supplementary energy sources, enhancing energy conversion efficiency while enabling mechanical adaptability. This approach offers several advantages particularly beneficial for flexible applications: reduced charging potentials, enhanced energy conversion efficiency, improved cycling stability under mechanical stress, minimized side reactions during deformation, and superior operational durability in varying geometric configurations [8, 242]. The development of flexible photo-assisted zinc battery systems requires systematic consideration of materials selection, interfacial engineering, and device architecture optimization to achieve synergistic performance enhancement between photoelectrochemical functionality and mechanical resilience essential for wearable and conformable energy storage applications.

**Fig. 19:**
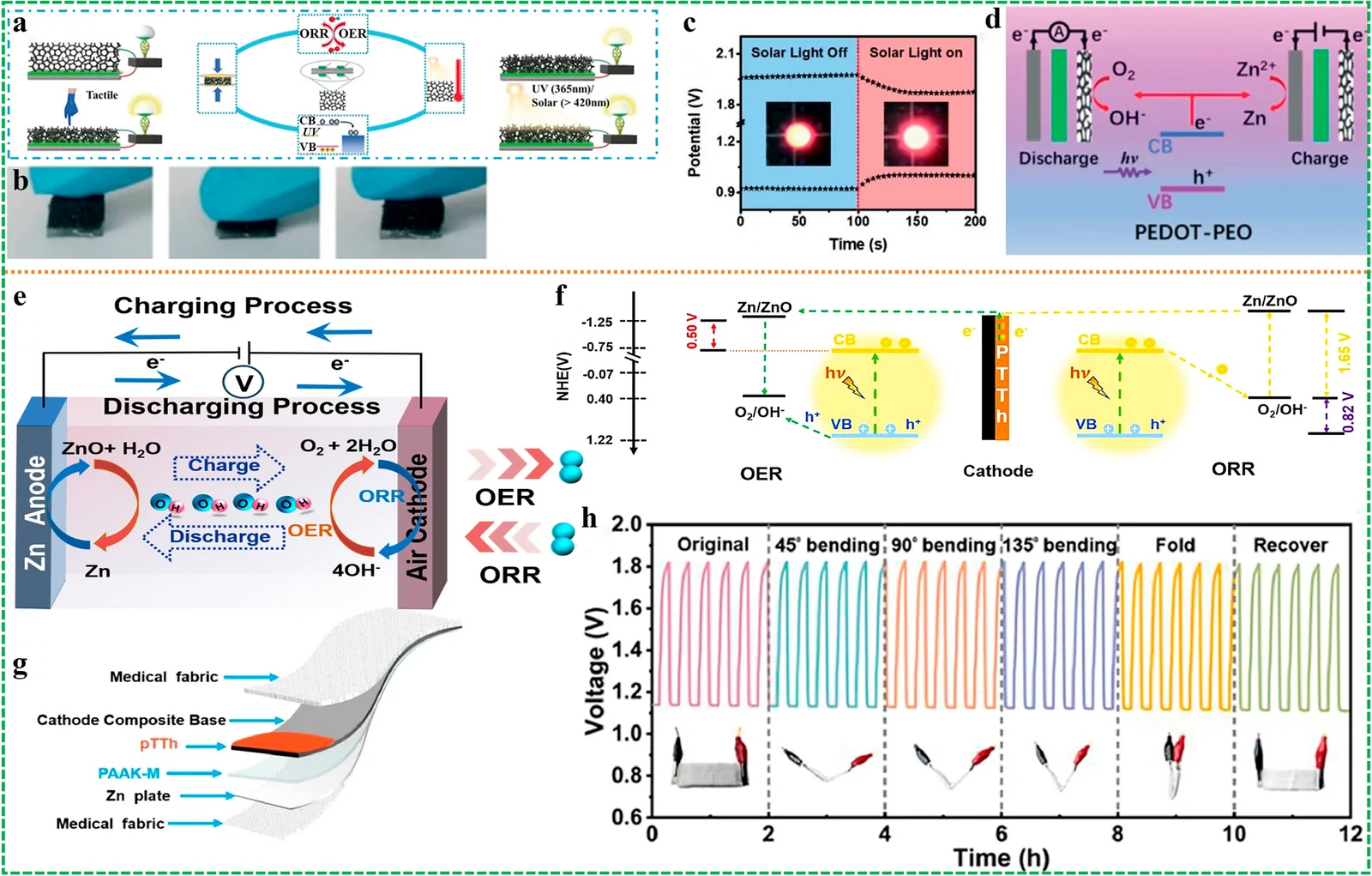
**a** SRZAB schematic diagram. **b** Compression-recovery process of SRZAB. **c** Galvanostatic charge–discharge curves of SRZAB in the dark and under sunlight (300 mW cm^−2^,1 mA cm^−2^). Inset: the enhanced brightness of red LED under sunlight. **d** Working principle of the UV light-responsive SRZAB. Reproduced with permission [243]. Copyright 2019, John Wiley and Sons Ltd. **e** Structure diagram of rechargeable ZABs. **f** ORR/OER mechanism diagram of photo-assisted RZABs. **g** Structure diagram of FZAPBs. **h** Charge–discharge cycling stability at different bending angles and at 2-h bending interval times at 0.1 mA cm^−2^. Reproduced with permission [177]. Copyright 2024, Elsevier

The development of efficient multifunctional air electrodes for flexible solar-rechargeable zinc-air batteries present unique challenges in integrating ORR and OER bifunctional catalytic capabilities within mechanically adaptable platforms. Breakthrough developments in all-solid-state flexible solar-rechargeable zinc-air batteries feature multi-stimuli responsive metal-free air electrodes fabricated using PEDOT-PEO and carbon nanotubes integrated into macroscopic polyurethane foam substrates (Fig. 19a) [243]. This innovative design endows the metal-free multi-sensing air electrode (MSAE) with mechanical compressibility allowing repeated compression with full recovery, enhanced mass transfer capabilities, and improved thermal effects essential for flexible energy storage applications (Fig. 19b). This outstanding structural toughness is achieved thanks to MSAE's polyurethane foam (PUF)-based 3D macroporous skeleton, which can withstand 80% compression strain and fully recover, while maintaining virtually unchanged resistance after 500 repeated compressions (80% strain). Through multi-scale engineering design, the MSAE achieves integrated ORR/OER bifunctional catalytic activity, pressure sensitivity, and photothermal and photoelectric conversion effects while maintaining structural integrity under mechanical deformation. The flexible solar-rechargeable zinc-air battery demonstrates impressive performance metrics including energy efficiency up to 69% and energy density of 1272 Wh L^−1^ with excellent cycling stability under various mechanical configurations. Under illuminated conditions, the charging potential decreased from 1.96 to 1.88 V while discharging potential increased from 0.92 to 1.00 V, improving energy efficiency from 46.9% in dark conditions to 53.2% (Fig. 19c). These improvements stem from the MSAE’s photoelectric conversion effect, which effectively modulates electrode redox reactions during both charging and discharging processes while maintaining performance under mechanical stress (Fig. 19d).

Polythiophene (pTTh) demonstrates exceptional potential for flexible photo-assisted zinc-air battery applications as a p-type semiconductor polymer with broad absorption spectrum and efficient solar energy utilization capabilities [16, 244]. The material exhibits exceptional photostability and direct applicability to flexible substrates, facilitating assembly of flexible batteries with bifunctional characteristics suitable for portable electronics and wearable devices [245]. Advanced implementation of pTTh as bifunctional catalysts in flexible rechargeable zinc-air batteries successfully addresses high polarization and low round-trip efficiency challenges while maintaining mechanical adaptability (Fig. 19e, f) [177]. Flexible zinc-air batteries feature a lightweight, thin design combined with PAAK-M double cross-linked gel electrolyte, making them small, lightweight, and adaptable to bending, folding, and other deformations. Under illumination, pTTh cathode-based flexible zinc-air batteries demonstrate 56.25% increase in power density, achieving discharge and charge voltages of 1.2 and 1.8 V respectively at 0.1 mA cm^−2^ current density. The practical applications of flexible zinc-air pouch batteries range from LED illumination to smartphone charging through series-connected configurations, validating their potential for wearable energy systems (Fig. 19g). The pTTh/CCB cathode-based flexible zinc-air pouch batteries exhibit excellent flexibility under various bending angles and intervals, representing significant advancement in mechanically adaptable rechargeable zinc-air battery technology with substantial practical application potential (Fig. 19h).

Zinc batteries demonstrate multiple significant advantages in flexible applications, with their core strength lying in the efficient integration of safety and functionality: the use of aqueous or gel electrolytes fundamentally eliminates the flammability risks associated with traditional batteries, and zinc's abundant reserves and low cost make it ideal for the large-scale deployment of flexible electronic devices; Additionally, by combining flexible substrates (such as titanium foil or carbon cloth) with gel electrolytes (such as PAAK-M), zinc batteries can withstand complex deformations like bending, folding, and compression, making them suitable for wearable devices, portable sensors, and other applications. They can even integrate light-responsive materials to enable light-assisted charging, thereby enhancing energy utilization efficiency. In terms of mechanical stability, zinc batteries have achieved breakthrough progress through multi-dimensional optimization: gel electrolytes, with their dual cross-linked structure, combine high elasticity and water retention, and can quickly recover and maintain ion conductivity even after repeated folding and twisting; electrodes utilize 3D porous structures (such as polyurethane foam frameworks) to effectively distribute stress; additionally, the device demonstrates excellent performance stability during long-term cycling. These characteristics collectively establish zinc batteries as a core competitive force in the field of flexible energy storage.

While photo-assisted flexible zinc batteries have achieved significant progress through advanced photoelectric materials, heterojunction structures, and bifunctional catalysts, developing stable and highly active photoelectrodes for flexible applications remains challenging. Key development priorities for flexible systems include: (1) long-term stability under continuous illumination and mechanical stress; (2) optimizing synergistic effects between photo and electrochemical processes in deformable architectures; (3) maintaining high catalytic activity for both ORR and OER under mechanical deformation; (4) ensuring mechanical robustness of photoelectrode materials during repeated bending and stretching. Furthermore, advancing flexible photo-assisted zinc batteries requires in-depth investigation of ORR and OER mechanisms within mechanically dynamic environments, understanding how mechanical stress affects photogenerated carrier behavior and reaction kinetics. This fundamental understanding will enable rational design of more efficient flexible photoelectrode materials and optimize overall performance of photo-assisted flexible zinc battery systems for next-generation wearable and conformable energy storage applications.

### Other Photo-Assisted Flexible Energy Storage Devices

Beyond lithium and zinc-based systems, photo-assisted flexible energy storage devices encompass diverse metal chemistries including magnesium, tin, and other metal-air configurations that offer unique advantages for flexible applications through strategic integration of photoelectrochemical enhancement with mechanical adaptability. These alternative metal systems present distinct integration challenges and opportunities for flexible energy storage, requiring specialized materials engineering approaches and device architecture optimization to achieve synergistic performance enhancement between photoelectrochemical functionality and mechanical resilience. The development of flexible photo-assisted multi-metal energy storage systems necessitates comprehensive understanding of metal-specific electrochemical behaviors, photoelectrochemical enhancement mechanisms, and their interactions under mechanical stress to enable rational design of next-generation wearable and conformable energy devices.

**Fig. 20:**
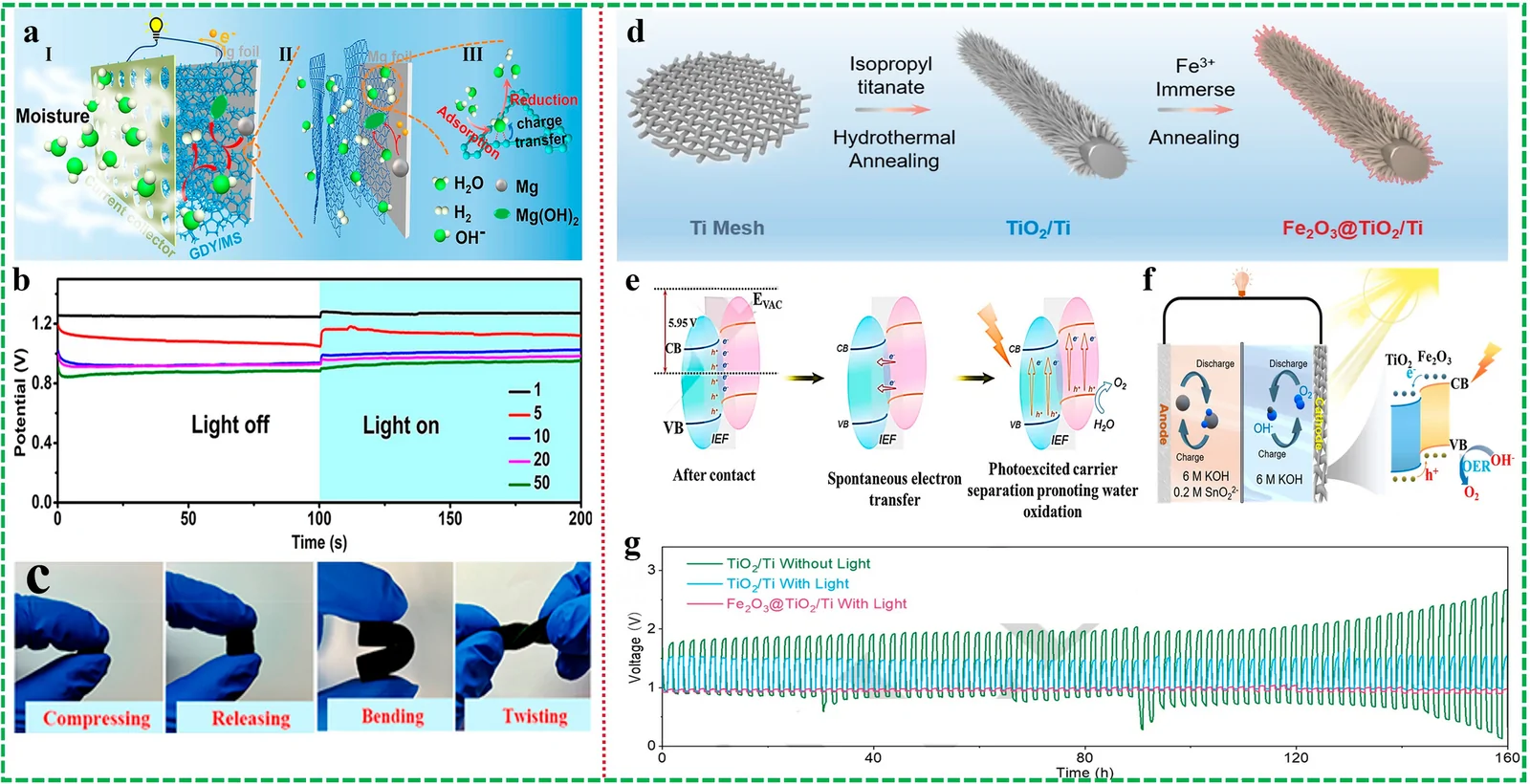
**a** Schematic illustration of the working mechanism and interfacial process of the GSMB. **b** Discharge curves of GSMB at various current densities (1–50 mA g^−1^) with and without illumination. **c** Photograph of the flexible GDY/ MS. Reproduced with permission [247]. Copyright 2023, American Chemical Society. **d** Schematic illustration for the synthesis of Fe_2_O_3_@TiO_2_/Ti. **e** Schematic illustration of the built-in electric field and photoexcited reaction on Fe_2_O_3_@TiO_2_/Ti. **f** Schematic charge and discharge processes of the light-assisted Sn-air battery. **g** Cycling performances of the rechargeable with Fe_2_O_3_@TiO_2_/Ti or TiO_2_/Ti-based Sn-air batteries at a current density of 0.1 mA cm^−2^ with or without light. Reproduced with permission [180]. Copyright 2024, John Wiley and Sons Ltd

Magnesium-based energy storage systems demonstrate significant potential for flexible applications due to magnesium’s lightweight characteristics [246], abundant resources, and high theoretical capacity, though implementation faces unique challenges in aqueous environments where hydrogen evolution reaction kinetics at cathodes create performance limitations. Advanced integration strategies employ hybrid structural materials combining graphdiyne (GDY) nanosheet arrays with three-dimensional melamine sponge scaffolds (GDY/MS) to create multifunctional cathodes for flexible solid magnesium-water batteries (Fig. 20a) [247]. The GDY/MS architecture functions simultaneously as humidity-sensitive unit, catalyst, and photoelectrode, enabling continuous performance modulation through both environmental humidity and solar illumination while maintaining structural integrity essential for flexible device applications. The naturally low bandgap of GDY (1.98 eV) facilitates efficient light capture and photoelectron generation, enhancing output current in flexible configurations where light exposure may vary with mechanical deformation. The smart magnesium-water battery system demonstrates exceptional rate capability and stability across various current densities (1–50 mA g^−1^) while maintaining mechanical flexibility through the sponge-based architecture that accommodates deformation without performance degradation (Fig. 20b, c). Under illumination, constant current curves reveal increased discharge potentials, improving battery performance and energy density with discharge potential increases of 0.13 V at 5 mA g^−1^, indicating effective photo-to-electrochemical energy conversion suitable for flexible and wearable applications. This innovative integration strategy opens pathways for developing self-powered flexible devices, smart wearable electronics, and intelligent battery systems that respond to environmental stimuli.

Tin-based energy storage systems offer unique advantages for flexible applications including prevention of dendritic growth during cycling, large reversible capacity, excellent hydrogen evolution resistance, and superior corrosion resistance—characteristics particularly valuable for flexible devices where mechanical stress might otherwise exacerbate performance degradation. However, implementation in flexible tin-air battery configurations faces kinetic limitations in ORR and OER at air cathodes, resulting in high overpotentials that substantially impact cycling efficiency and reversibility, particularly challenging for flexible systems requiring consistent performance across various mechanical states. Strategic integration of photoelectrochemical enhancement through Fe_2_O_3_-modified TiO_2_ nanorods with heterogeneous structure represents breakthrough developments for flexible tin-air battery applications (Fig. 20d) [180]. The core–shell heterojunction structure of Fe_2_O_3_@TiO_2_ effectively promotes electrochemical intermediate conversion and separation of photo-excited electrons and holes while maintaining structural integrity necessary for flexible substrate integration. The heterojunction creates strong internal electric fields through photogenerated electron transfer from Fe_2_O_3_ to TiO_2_, achieving Fermi level equilibrium that enhances both electrocatalytic and photocatalytic activities essential for flexible energy storage performance (Fig. 20e). The flexible tin-air battery architecture incorporating Fe_2_O_3_@TiO_2_ cathode catalysts demonstrates ultra-low overpotentials of approximately 40 mV, exceptional rate capability, and excellent photocycling stability under various mechanical configurations (Fig. 20f, g). This represents the first successful application of photoelectric effect catalysts in flexible tin battery systems, achieving ultra-low overpotential performance while maintaining mechanical adaptability essential for wearable and conformable energy storage applications.

The integration of advanced two-dimensional materials including NiFe layered double hydroxides (LDH) with tin metal demonstrates remarkable promise for flexible battery applications through their inherent compatibility with flexible substrate architectures. NiFe LDH’s two-dimensional nature supports facile flexible film formation while providing high surface area and abundant active sites particularly effective for OER catalysis in flexible electrode configurations. Tin metal's unique advantages including dendrite-free growth, large capacity, and corrosion resistance prove particularly valuable for flexible anodes where mechanical stress might otherwise exacerbate performance degradation. Strategic integration with photoelectrode materials and optimization of flexible battery architectures enable photo-assisted multi-metal batteries to achieve remarkable performance enhancement suitable for wearable and conformable applications. When combined with semiconductors like TiO_2_ or Fe_2_O_3_ to form heterojunction structures on flexible substrates, these systems achieve enhanced charge separation and broader light absorption while maintaining mechanical resilience. The synergistic effects between photoelectrode and electrocatalyst materials significantly reduce charging overpotentials and improve energy conversion efficiency in mechanically dynamic environments.

Future development priorities for photo-assisted flexible multi-metal energy storage systems should focus on advancing scalable synthesis methodologies for flexible photoelectrode materials, developing robust integration strategies that maintain performance under repetitive mechanical cycling, and optimizing device architectures that maximize both photoelectrochemical efficiency and mechanical resilience. Critical research directions include expanding the range of compatible metal chemistries, enhancing long-term stability under combined optical and mechanical stress, and developing integrated manufacturing processes compatible with flexible substrate requirements. These systematic developments are essential for realizing high-performance, cost-effective flexible energy storage systems suitable for next-generation wearable electronics, smart textiles, and conformable devices where mechanical adaptability and energy density are fundamental requirements.

## Challenges and Future Prospects for Photo-Assisted Flexible Energy Storage Devices

Photo-assisted flexible energy storage devices, comprising photoelectrodes or photosensitive materials integrated with mechanically adaptable electrochemical energy storage units including SCs and various battery systems, have garnered significant attention for their direct solar-to-chemical energy conversion and storage capabilities within conformable architectures. While these integrated flexible systems demonstrate unique advantages in energy conversion efficiency, multifunctionality, mechanical adaptability, portability, and operational durability, several critical challenges specific to flexible implementations impede their widespread commercialization and practical deployment in wearable and conformable energy applications. A comparison of different light-assisted flexible energy storage systems is shown in Table 7.

**Table 7:** Comparison of different light-assisted flexible energy storage systems

Light-assisted flexible energy storage system types	Advantages	Disadvantages	Application scenarios
SC	Extremely high power density and fast charging–discharging speed Extremely long cycle life and excellent bending resistance of flexible substrates (such as carbon cloth and polymers) Light-assisted enhancement of the double layer/pseudocapacitive effect through photo-generated charges, improving specific capacitance Simple structure and low cost	Low energy density, far below that of battery systems Long-term exposure to light may cause photo-corrosion of electrode materials, affecting service life Energy storage mechanism relies on surface reactions, limiting capacity improvement potential	Flexible wearable sensors (such as heart rate and body temperature monitoring devices), high-frequency charging and discharging devices (foldable keyboards, flexible displays)
Li–O_2_ battery	Extremely high theoretical energy density, with light assistance reducing overpotential during charging and discharging Photocatalytic enhancement of ORR/OER activity, improving energy conversion efficiency Compatible with flexible substrates, with excellent bending performance	Poor cycle stability Lithium dendrite issues have not been completely resolved, posing safety hazards Flexible substrates (such as polyimide) may be swollen by the electrolyte, affecting mechanical stability	High-energy demand flexible devices (such as flexible drone power supplies and foldable solar power banks)
Li–CO_2_ battery	High theoretical energy density, light assistance can promote CO_2_ reduction reaction kinetics, suitable for CO_2_ recycling in enclosed spaces Flexible structure and good compatibility with solid electrolytes, excellent bend resistance	CO₂ diffusion is greatly affected by deformation, and the bending angle is limited The cycle life is extremely short, CO_2_ adsorption/desorption efficiency is low, and by-products (Li_2_C_3_) are difficult to decompose Photocatalytic materials are easily eroded by Li^+^, resulting in poor long-term stability	Flexible energy storage in closed environments (such as flexible sensor power supplies for space stations and flexible equipment for deep-sea exploration)
Li–S battery	High theoretical energy density Photocatalytic inhibition of polysulfide shuttling, greatly extending cycle life Abundant sulfur resources, good compatibility between flexible substrates and sulfur electrodes	High volume expansion rate, flexible structures are prone to cracking due to expansion Limited light response wavelength, weak enhancement effect under visible light Some light-responsive materials are toxic, limiting biocompatibility	Medium- to high-energy flexible wearable devices (such as smart bracelets and flexible electronic skin power supplies)
Li–N_2_ battery	Raw material (N_2_) is inexhaustible (air source) and theoretically suitable for long-term energy storage Light assistance can activate the inert bond of N₂ (lowering the activation energy) and preliminarily achieve the N_2_ reduction reaction	The reaction kinetics are extremely poor, and the energy conversion efficiency is low The cycle life is extremely short	Specialized energy storage applications (e.g., flexible backup power sources for aerospace and deep-sea exploration)
Zn battery	Water-based electrolyte (high safety, no risk of combustion or explosion), abundant zinc resources (low cost) Light assistance can increase the oxygen evolution overpotential, inhibit water decomposition, and improve cycle life Excellent mechanical flexibility	Low energy density and limited low-temperature performance ORR/OER kinetics still need to be improved	Everyday flexible electronics (such as flexible watches and smart clothing power sources)
Mg battery	Rich in magnesium resources (low cost), high volumetric energy density Better safety than lithium-based batteries Excellent flexibility and structural stability	Limited choice of cathode materials, limited improvement with light assistance Low energy density, insufficient competitiveness Poor electrolyte compatibility	Low-cost flexible electronics (flexible remote controls, electronic tags, foldable card-type devices)
Sn battery	The theoretical capacity of tin negative electrode is relatively high, and light assistance can suppress tin volume expansion and improve cycle life Tin resources are abundant and the cost is moderate No dendrite risk, good safety Good compatibility with flexible substrates	Low energy density Limited bending angle, average deformation tolerance	Medium–low endurance flexible devices (flexible keyboards, electronic skin, smart curtain sensors)

### Current Limitations and Flexible Integration Challenges

**Fig. 21:**
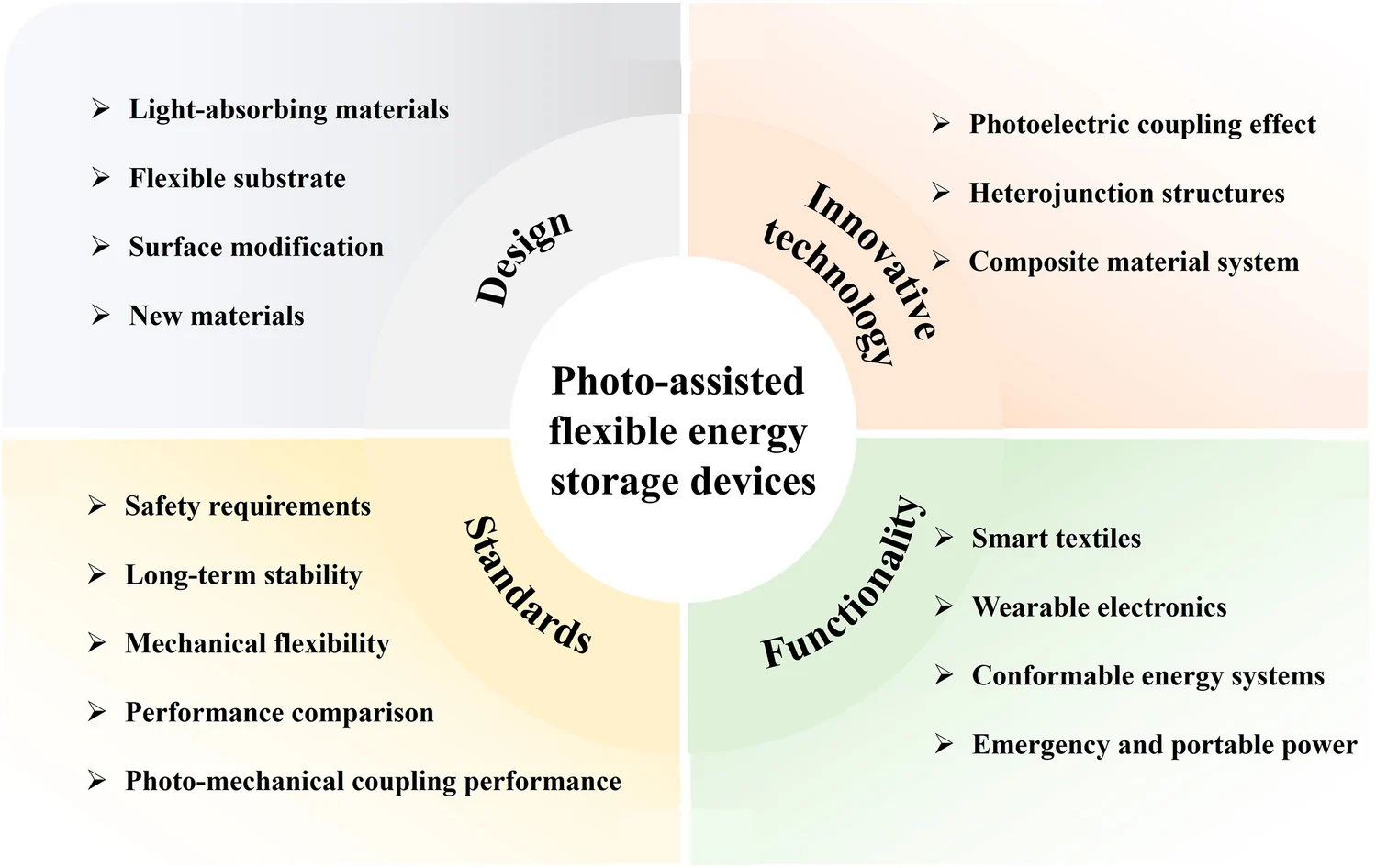
Future perspectives for the design and development of photo-assisted flexible energy storage devices

Despite significant advances across various flexible photo-assisted energy storage systems including SCs, lithium batteries, zinc batteries, and alternative metal systems, the field remains in early developmental stages with numerous technological hurdles specific to flexible device implementation requiring systematic resolution (Fig. 21). The integration of photoelectrochemical functionality with mechanical flexibility introduces complex design challenges that extend beyond conventional rigid energy storage systems, necessitating comprehensive understanding and innovative solutions to achieve optimal performance in mechanically dynamic environments.

Primary limitations include: (1) Materials and manufacturing constraints for flexible systems: High production costs stemming from expensive photoactive electrode materials compatible with flexible substrates, specialized flexible electrolytes, and complex manufacturing processes required for maintaining both optical and mechanical functionality. Limited material selection and compatibility issues between photoelectrochemical components and flexible substrates result in suboptimal photovoltaic conversion efficiencies and increased interfacial resistivity, particularly problematic where mechanical stress exacerbates contact resistance; (2) Energy conversion efficiency under mechanical deformation: The fundamental challenge of maintaining solar-to-electrical/chemical energy conversion efficiency across various mechanical states while preserving device stability, considering potential adverse reactions between photosensitive materials and flexible electrodes or electrolytes under mechanical stress; (3) Long-term stability in flexible configurations: The necessity to maintain consistent performance under varying illumination conditions, repeated mechanical cycling, and material degradation, especially with flexible electrolyte systems prone to redistribution, leakage, or degradation during deformation; (4) Mechanistic understanding of flexible systems: Insufficient comprehension of photoelectrochemical conversion processes under mechanical stress and structural evolution during combined solar energy conversion and mechanical deformation; and (5) Standardization for flexible devices: The absence of standardized testing protocols and long-term viability assessments specifically designed for flexible energy storage systems. These challenges necessitate systematic research approaches focusing on flexible materials design, strain-tolerant interface engineering, mechano-electrochemical mechanism elucidation, and specialized standardization protocols for flexible energy storage applications.

### Future Research Directions for Flexible Integration

#### Design and Development of Mechanically Resilient Light-Absorbing Materials

The enhancement of photo-assisted flexible energy storage systems requires strategic design, synthesis, and integration of novel materials that maintain photoelectrochemical functionality under mechanical deformation. Traditional rigid photoactive materials like TiO_2_, with their wide bandgaps and brittle nature, prove inadequate for flexible applications due to limited light absorption ranges and mechanical brittleness that compromises device integrity during flexing [164]. Advanced development focuses on mechanically resilient narrower bandgap semiconductor materials through strategic doping and flexible substrate integration, including titanium oxide composites and cadmium sulfide systems that demonstrate broader light absorption ranges and improved mechanical tolerance essential for flexible energy storage applications [248].

The integration of light-absorbing materials with flexible conductive substrates significantly enhances photogenerated carrier separation efficiency and photoelectrocatalytic performance while maintaining mechanical adaptability. Strategic doping with non-precious metals including Mo, Co, and Ni enables adjustment of band structures and photoelectric properties while reducing costs and improving stability in flexible configurations [150]. Surface modification techniques specifically developed for flexible substrates, including conformal thin-layer deposition and strain-tolerant coating treatments, enhance surface properties and improve both light absorption and photoelectrocatalytic efficiency under mechanical stress. For instance, depositing flexible photosensitizer layers on semiconductor surfaces effectively expands light absorption ranges while maintaining mechanical integrity during device deformation [249].

Advanced flexible architectures incorporating porous MOFs and COFs demonstrate significant potential due to their abundant active sites and inherent flexibility when properly engineered. Their strategic integration into flexible photo-assisted energy storage device electrodes significantly improves rate capability, cycling stability, and mechanical resilience essential for wearable applications [138, 250]. This comprehensive approach to flexible materials design represents crucial advancement in developing next-generation mechanically adaptable photo-assisted energy storage systems.

#### Innovative Technologies for Enhanced Charge Storage in Flexible Architectures

The advancement of photo-assisted flexible energy storage devices relies on synergistic integration of photoelectric effects with flexible energy storage mechanisms that maintain performance across various mechanical states. Under illumination, photosensitive materials integrated within flexible architectures absorb light energy to generate photoexcited electrons and holes, which undergo efficient separation and transport through strain-tolerant pathways to participate in energy storage processes including battery charging–discharging cycles and capacitor charge accumulation while accommodating mechanical deformation.

Optimization of solar energy utilization in flexible systems employs materials with superior light absorption properties including narrow-bandgap semiconductors and flexible perovskite materials engineered to achieve broader spectral absorption ranges while maintaining mechanical flexibility [251]. Surface microstructure engineering and strategic doping modifications specifically designed for flexible applications enhance light absorption capabilities and photoelectric conversion efficiency under mechanical stress [151]. The design of strain-tolerant photoelectric coupling effects ensures efficient separation and transport of photogenerated carriers while minimizing recombination losses during mechanical deformation.

Advanced flexible device architectures incorporating multilayer heterojunction structures or composite material systems leverage band alignment differences and interfacial effects to promote directional migration of photogenerated carriers and energy conversion while maintaining structural integrity under mechanical stress [252]. Integration of intelligent control systems enables automatic adjustment of flexible device operation based on illumination intensity, mechanical state, and energy storage requirements, achieving smart matching between photonic and electrical energy in mechanically dynamic environments. Computational approaches including density functional theory (DFT) and molecular dynamics (MD) simulations specifically address flexible system behavior, revealing interactions between photoelectric effects and mechanical deformation at atomic and mesoscopic scales, providing theoretical foundations for optimizing flexible device architectures and predicting next-generation flexible photoactive materials.

#### Expanding Applications in Wearable and Conformable Systems

Photo-assisted flexible energy storage devices demonstrate extensive application potential across wearable electronics, smart textiles, and conformable energy systems where mechanical adaptability is essential. In wearable technology applications, these flexible systems integrate seamlessly into clothing, accessories, and body-worn devices to provide continuous energy harvesting and storage capabilities that adapt to user movement and environmental conditions. Smart textile implementations incorporate flexible photo-assisted energy storage directly into fabric structures, enabling self-powered wearable electronics including health monitoring devices, communication systems, and environmental sensors.

Conformable energy systems for curved and irregular surfaces demonstrate significant potential in applications including flexible displays, rollable electronics, and adaptive building-integrated energy systems where traditional rigid energy storage proves inadequate. The integration of flexible photo-assisted energy storage into Internet-of-Things (IoT) devices enables autonomous operation in remote or mobile applications where conventional power sources are impractical. Medical device applications including flexible biosensors, implantable energy harvesters, and wearable therapeutic devices benefit from the combined energy harvesting and storage capabilities while maintaining biocompatibility and mechanical conformability essential for medical applications.

Emergency and portable power applications leverage the mechanical adaptability and energy autonomy of flexible photo-assisted systems for disaster response, military applications, and outdoor activities where robust, lightweight, and mechanically resilient energy storage is critical. As flexible electronics and wearable technology markets continue expanding, photo-assisted flexible energy storage devices are positioned to enable new application paradigms that were previously impossible with rigid energy storage systems.

#### Establishment of Specialized Standards for Flexible Energy Storage Systems

The development of comprehensive industry standards specifically designed for photo-assisted flexible energy storage devices represents a critical imperative for ensuring product quality, safety, and performance consistency in mechanically dynamic applications. Unlike rigid energy storage systems, flexible implementations require specialized testing protocols that address the complex interactions between photoelectrochemical performance, mechanical flexibility, and long-term stability under combined optical and mechanical stress conditions.

Essential standardization areas include: (1) Mechanical flexibility assessment protocols that evaluate device performance across various bending radii, stretching ratios, and twisting angles while maintaining photoelectrochemical functionality; (2) Photo-mechanical coupling performance metrics that quantify energy conversion efficiency under different illumination conditions and mechanical states; (3) Long-term stability evaluation methodologies specifically designed for flexible systems including accelerated aging tests under combined mechanical cycling and optical exposure; (4) Safety requirements for wearable applications addressing biocompatibility, electrical safety, and mechanical integrity standards essential for body-worn energy storage devices; (5) Performance comparison frameworks enabling systematic evaluation and comparison of different flexible photo-assisted energy storage technologies and architectures.

Specifically, the standardized testing scheme for flexible light-assisted devices should be structured around mechanical performance, optoelectronic performance, and their synergistic effects to ensure the objectivity of performance evaluation and the comparability of data. In terms of mechanical testing, the bending radius (recommended to be set within a gradient range of 5–10 mm to cover typical scenarios such as wearable devices and curved surfaces), deformation modes (static bending, dynamic repetitive folding, torsion, etc.), and cycle counts (recommended ≥ 1000 cycles to simulate fatigue effects from long-term use). Deformation levels should be precisely quantified using strain sensors or optical micrometer methods to avoid subjective judgment errors. Photovoltaic performance testing requires standardized lighting conditions, including simulation of solar intensity, control of illumination duration and spot uniformity, and standardized testing procedures for core parameters such as photocurrent density, photovoltaic conversion efficiency, and open-circuit voltage, while simultaneously recording performance differences between dark and light states. The key lies in establishing a light-mechanical coordination testing system, i.e., simultaneously applying light illumination under predefined bending/folding conditions while real-time monitoring the dynamic changes in photoresponse performance (such as photogenerated carrier migration rate and charge separation efficiency), simulating the “light-force” coupling conditions in actual applications to avoid performance misjudgment caused by single-condition testing. Additionally, it is necessary to define device failure thresholds (e.g., capacity retention rate below 80% or photovoltaic efficiency degradation exceeding 30%) and stability assessment cycles (e.g., performance degradation rate after 100 h of continuous illumination). This standardized testing scheme addresses the current issues of inconsistent testing parameters and difficulty in cross-comparing data, providing a unified benchmark for material optimization, device design, and industrialization, thereby advancing flexible light-assisted technology from the laboratory to practical applications.

The establishment of these specialized standards will facilitate meaningful technology comparisons, accelerate innovation through clear development targets, ensure consumer safety and confidence, and support regulatory compliance essential for commercialization. Standardization efforts must address the unique challenges of flexible energy storage including mechanical durability requirements, environmental tolerance specifications, and integration compatibility with flexible electronic systems. This comprehensive standardization framework will provide robust foundation for sustainable development and widespread adoption of photo-assisted flexible energy storage technologies in next-generation wearable and conformable energy applications.

## Conclusions

The development of photo-assisted flexible energy storage devices marks a transformative advancement in sustainable energy technology, providing innovative solutions to meet the rising demand for mechanically adaptable, self-powered energy storage systems. A comprehensive analysis of recent research across diverse device architectures—including flexible SCs, lithium-based batteries, zinc batteries, and alternative metal systems—has identified key achievements and technological breakthroughs, which highlight the significant progress and potential of this rapidly evolving field.

**Material Innovation and Integration Achievements**: The systematic development of advanced photoactive materials, specifically designed for flexible applications, has yielded remarkable breakthroughs in retaining both photovoltaic conversion efficiency and mechanical resilience. Notable advancements include MOF-based composite electrodes with exceptional cycling stability, defect-engineered carbon nitride systems with enhanced photocatalytic activity, and strain-tolerant semiconductor heterostructures that retain photoelectrochemical functionality under mechanical deformation. Advanced composite material systems have successfully tackled the fundamental challenge of preserving optical and electrochemical properties across diverse mechanical states, paving viable pathways for the implementation of mechanically adaptable energy storage devices.

**Performance Milestones and Technological Validation**: Notable performance gains have been observed across various device categories, confirming the practical feasibility of photo-assisted flexible energy storage systems. Flexible SCs have exhibited significant capacity improvements under illumination while retaining excellent mechanical durability; some systems maintain over 90% capacity after thousands of bending cycles. Photo-assisted flexible lithium batteries have demonstrated notable improvements in energy density and cycling stability; photo-assisted lithium-based flexible batteries (PALSBs) exhibit ultra-low overpotentials and exceptional rate capabilities. Flexible zinc-based systems have shown impressive energy efficiencies exceeding 60%, with successful integration of multi-stimuli responsive functionalities, whereas alternative metal systems have achieved breakthroughs in overpotential reduction and direct photo-charging capabilities.

**System Integration and Architectural Innovation**: The successful integration of photoelectrochemical conversion and flexible energy storage functionalities has been realized via innovative device architectures enabling autonomous operation. Multifunctional electrode designs integrating catalytic, photoactive, and mechanical properties simultaneously have enabled seamless energy harvesting and storage in single device platforms. Advanced electrolyte formulations and separator technologies, specifically developed for flexible configurations, have tackled key stability and performance challenges, whereas innovative interconnection strategies have preserved electrical performance under diverse deformation states.

**Technological Maturity and Application Readiness**: The field has advanced from proof-of-concept demonstrations to practical device implementations with real-world application potential. Successful demonstrations include wearable energy systems that power electronic devices, flexible energy storage integrated into textiles and conformable surfaces, and autonomous energy-harvesting systems suitable for IoT applications. These achievements confirm the transition from laboratory research to potentially viable commercial technologies, positioning photo-assisted flexible energy storage as a promising solution for next-generation portable and wearable energy applications.

**Impact and Significance**: The advancement of photo-assisted flexible energy storage technology marks a paradigm shift toward autonomous, mechanically adaptable energy systems that integrate renewable energy harvesting with high-performance energy storage. These advancements have unlocked new opportunities for wearable electronics, smart textiles, conformable IoT devices, and distributed energy systems—where traditional rigid energy storage solutions are inadequate. The successful integration of photoelectrochemical enhancement and mechanical flexibility has shown that high-performance energy storage and mechanical adaptability are not mutually exclusive, enabling new application paradigms once deemed technically unfeasible.

The collective advancements in photo-assisted flexible energy storage devices lay a solid foundation for ongoing technological progress and eventual commercialization. As this technology matures, it is poised to play increasingly critical roles in sustainable energy infrastructure, portable electronics, and emerging applications requiring both high performance and mechanical flexibility—contributing significantly to the broader transition toward sustainable, adaptable energy systems.
